# Electrochemical α-C(sp^3^)–H/N–H Cross-Coupling of Isochromans and Azoles

**DOI:** 10.3390/molecules30010004

**Published:** 2024-12-24

**Authors:** Guoping Li, Bing Yan, Liangliang Wu, Yabo Li, Xinqi Hao, Ming Gong, Yangjie Wu

**Affiliations:** College of Chemistry, Henan Key Laboratory of Chemical Biology and Organic Chemistry, Key Laboratory of Applied Chemistry of Henan Universities, Zhengzhou University, Zhengzhou 450052, China; liguoping@zzu.edu.cn (G.L.); yb1151869573@163.com (B.Y.); wuliangliang@gs.zzu.edu.cn (L.W.); ybli@zzu.edu.cn (Y.L.); xqhao@zzu.edu.cn (X.H.); wyj@zzu.edu.cn (Y.W.)

**Keywords:** electro-oxidation, cross-coupling, isochroman, azole, C–N bond formation

## Abstract

Isochroman and azole moieties are both present in a wide variety of biologically active molecules. Their efficient combination under mild reaction conditions is beneficial for obtaining small-molecule drug candidates. In this paper, we describe electrochemical α-C(sp^3^)–H/N–H cross-coupling reactions between isochromans and azoles, yielding products in moderate to excellent yields. This protocol does not require any catalysts or exogenous oxidants and can be performed at room temperature under air. Control experiments and cyclic voltammetry showed that the reaction may proceed through both radical coupling and nucleophilic addition processes.

## 1. Introduction

The cross-dehydrogenative coupling (CDC) reaction has attracted significant interest in recent years due to its ability to directly construct carbon–carbon (C–C)/carbon–heteroatom (C–X) bonds through the in situ activation of C–H/X–H bonds [1,2,3]. The traditional synthetic strategy usually employs stoichiometric amounts of oxidants to clean up surplus electrons from substrates, which unavoidably generate byproducts and limit the applicability of substrates [4,5,6]. With the evolution of more sustainable chemistry, the direct electro-oxidation method is considered an environmentally friendly and powerful synthetic method under exogenous-oxidant-free conditions [7,8,9,10,11]. In recent years, the electrochemically promoted direct dehydrogenative coupling of the C(sp^3^)–H bond has been applied to construct C–C [12,13,14], C–N [15,16,17,18,19,20,21], C–O [22,23,24], C–S [25,26], C–P [27] and C–X bonds [28,29,30]. To date, the construction of hybrid molecules through the direct coupling of non-functionalized heterocycles using the electrochemical CDC reaction under mild conditions remains a challenging task.

**Scheme 1:**
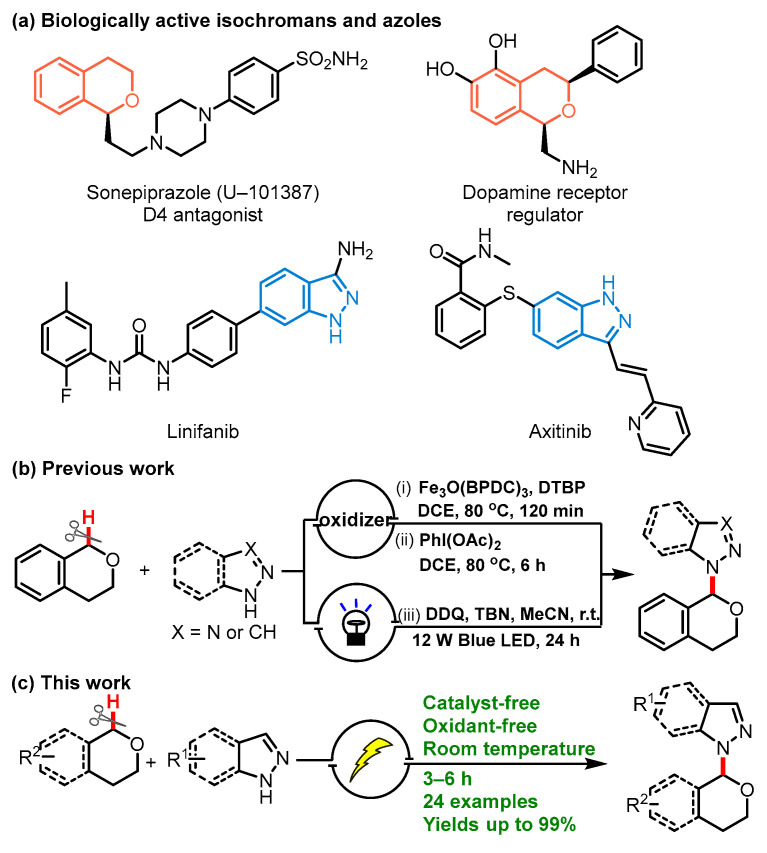
Cross-dehydrogenative coupling of isochromans and azoles.

Isochroman and azole moieties are present in a wide variety of biologically active molecules (Scheme 1a) [31,32]. More recently, the construction strategy of molecular hybridization with two or more natural product fragments has been applied to solve the problem of drug resistance [33]. The combination of isochromans and azoles will produce a novel hybrid molecule with potential biological activity. Currently, CDC reactions between isochromans α-C(sp^3^)–H and azoles N–H have been achieved using transition metal catalysts or/and excess oxidants or photo-oxidants, which results in an increase in chemical waste and economic costs (Scheme 1b) [34,35,36]. Therefore, it is necessary to explore an environmentally friendly and mild method of constructing such hybrid molecules. As a continuation of our efforts to develop sustainable organic synthesis methods in electrochemistry [17,37], herein, we report an electrocatalytic CDC reaction under catalyst- and oxidant-free conditions to synthesize C–N coupling products in a green and efficient manner (Scheme 1c). The possible reaction mechanism was explored experimentally.

## 2. Results and Discussion

We started our investigation by employing isochroman (**1a**) and indazole (**2a**) as template substrates (Table 1). The reaction showed minimal conversion in an undivided RVC/Ni cell at 10 mA without any additives (entry 1). Pleasingly, the introduction of trifluoromethanesulfonic acid (TfOH) significantly enhanced the reaction, yielding the target product **3a** with a 39% yield (entry 2). An excess amount of isochroman (**1a**) improved the yield of product **3a** (entry 4). Among the electrolytes tested, *n*-Bu_4_NPF_6_ proved to be the most effective (entries 5–7). The product yield could not be effectively improved using other electrodes or solvents (entries 8–10). Additionally, the yield of compound **3a** was also affected by both the current intensity and reaction time (entries 11–14). TfOH could promote electrosynthesis (entry 15). And the reaction afforded the product in 50% yield under an Ar atmosphere (entry 16).

**Scheme 2:**
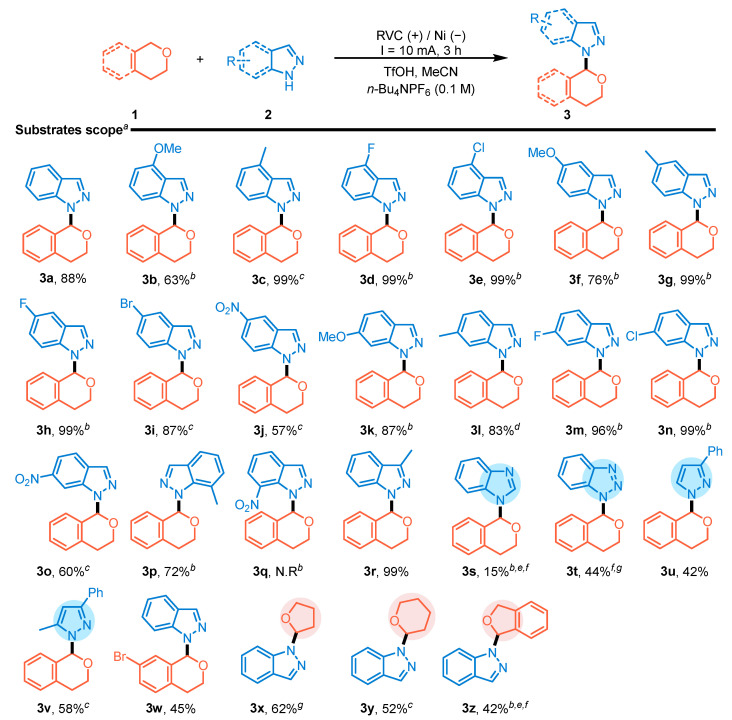
Substrate scope. ^a^ Standard conditions: **1** (1.2 mmol), **2** (0.3 mmol), I = 10 mA, *n*-Bu_4_NPF_6_ (0.1 M), TfOH (0.45 mmol), MeCN (6 mL), at room temperature for 3 h. ^b^ 6 h. ^c^ 5 h. ^d^ 4.5 h. ^e^ *n*-Bu_4_NBF_4_ instead of *n*-Bu_4_NPF_6_. ^f^ **1** (3.0 mmol). ^g^ 4 h.

**Scheme 3:**
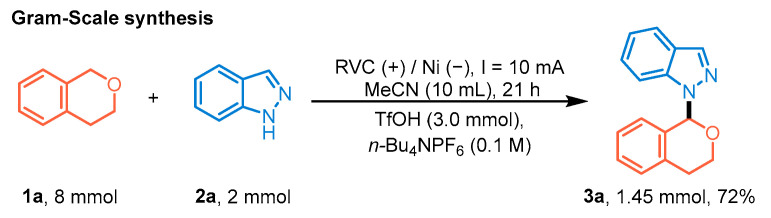
Gram-scale experiment.

With the optimal conditions in hand, the substrates’ scope was explored (Scheme 2). In order to achieve higher product yields, the reaction conditions were slightly adjusted for several substrates. Indazoles **2** with both electron-donating and weak electron-withdrawing groups were transformed into their corresponding products in good to excellent yields (**3a**–**i**, **3k**–**n** and **3r**). Due to the strong electron-donating nature of the methoxy group, it might have become more difficult for N–H bond cleavage in compounds **2b**, **2f** and **2k** to generate the active intermediates. This resulted in lower product yields (**3b**, **3f** and **3k**). Surprisingly, moderate yields were observed for substrates **2j** and **2o**, which contain strong electron-withdrawing groups. The product yield was highly susceptible to both steric hindrance (**3p**) and intramolecular hydrogen bonding (**3q**) [38]. In addition, other azoles, substituted isochroman (7-bromoisochroman) and cyclic ethers were also suitable for the reaction system (**3s**–**3z**). A gram-scale reaction afforded **3a** in a 72% yield, demonstrating the potential of this electrochemical method for industrial applications (Scheme 3).

**Scheme 4:**
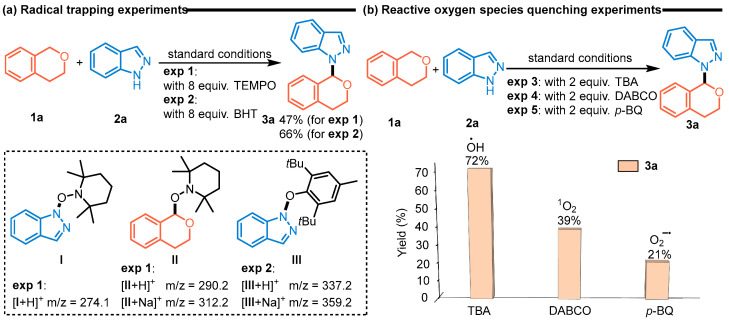
Control experiments.

**Figure 1:**
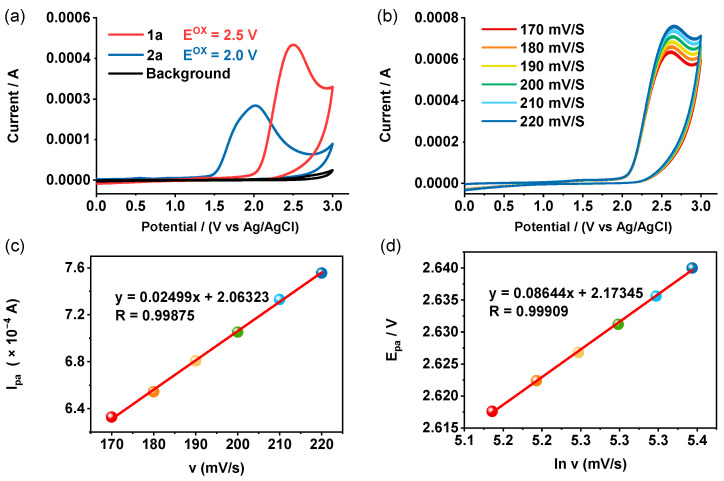
(**a**) The cyclic voltammetry of isochroman (**1a**) and indazole (**2a**) was measured in 0.05 M *n*-Bu_4_NPF_6_/MeCN using a glassy carbon (GC) disk as the working electrode, a Pt wire as the counter electrode and Ag/AgCl as the reference electrode at a 100 mV/s scan rate. (**b**) Cyclic voltammograms of isochroman (**1a**) at different scan rates. Curves were recorded at scan rates of 170, 180, 190, 200, 210 and 220 mV/s. (**c**) The plot of peak current vs. scan rate for isochroman (**1a**). (**d**) The relationship between E_pa_ and lnν for isochroman (**1a**).

To determine the detailed mechanism of this transformation, several control experiments were carried out (Scheme 4). Initially, an excess of 2,2,6,6-tetramethylpiperidine 1-oxide (TEMPO) or butylated hydroxytoluene (BHT) could not completely inhibit this reaction (exp **1** and **2**). Meanwhile, five strong molecular ion peaks were detected by LC-MS: [**I** + H]^+^ (exact mass: 274.1); [**II** + H]^+^ (exact mass: 290.2); [**II** + Na]^+^ (exact mass: 312.2); [**III** + H]^+^ (exact mass: 337.2); and [**III** + Na]^+^ (exact mass: 359.2). Triethylenediamine (DABCO) and 1,4-benzoquinone (*p*-BQ) as reactive oxygen species quenchers effectively inhibited the reaction (exp **3**–**5**). The oxidation peaks of isochroman (**1a**) and indazole (**2a**) were observed, respectively, at 2.5 V and 2.0 V vs. Ag/AgCl by cyclic voltammetry experiments. It was seen that the oxidation peak currents for isochroman (**1a**) increased linearly with scan rates (Figure 1b,c). The number of electrons for isochroman (**1a**) was calculated to be one by Laviron’s method [39] (Figure 1d). These results indicated the following: (1) the reaction might have involved both a radical process and an ionic pathway; (2) the oxidation of **1a** was an absorption-controlled process, and the electro-oxidation step of **1a** involved a one-electron process; and (3) the singlet oxygen (^1^O_2_) and superoxide anion (O_2_^−•^) might serve as the active species for this transformation.

**Scheme 5:**
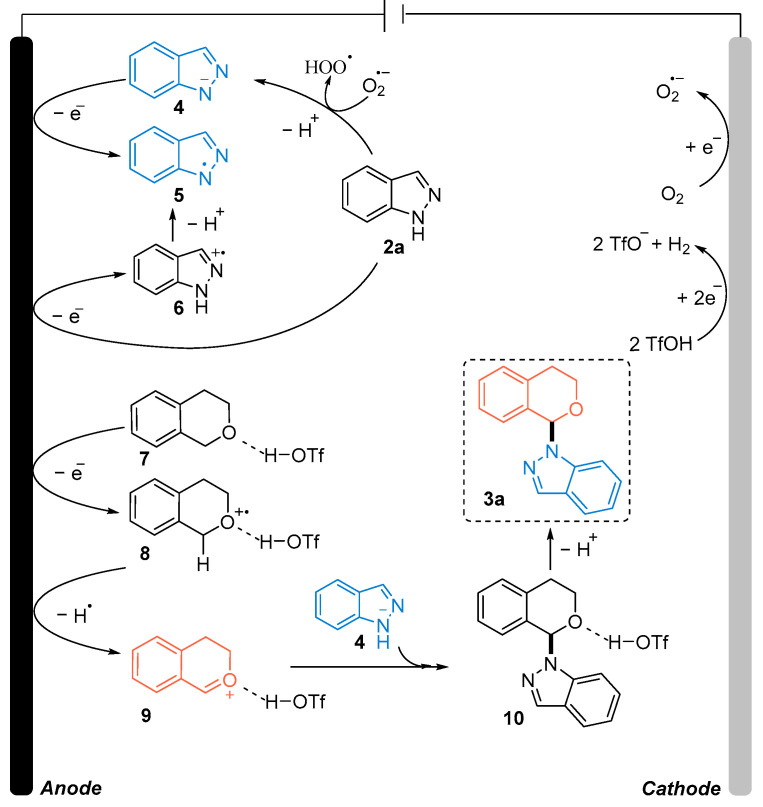
Plausible mechanism.

Based on the above results and the relevant literature [24], a possible reaction mechanism for this electrocatalytic CDC reaction was proposed (Scheme 5). Initially, the acid and oxygen could be reduced to hydrogen and superoxide anion (O_2_¯^•^) on the cathode. Superoxide anion (O_2_¯^•^) could facilitate the deprotonation of indazole (**2a**) to form anion **4**. Subsequently, the anion **4** underwent anodic oxidation to form radical **5**, which could also be generated from the direct oxidation of **2a**. Meanwhile, intermediate **7** was generated by the formation of a hydrogen bond between the O atom of isochroman and the hydrogen atom of TfOH. Intermediate **9** was formed on the anode through the stepwise oxidation of intermediate **7**. And **10** could be formed though the ion process between cation **9** and anion **4**. Finally, deprotonation generated the cross-coupling product **3a**.

## 3. Materials and Methods

### 3.1. Materials and Instruments

All reagents were obtained from commercial sources without further purification unless otherwise stated. Solvents were dried and degassed by standard methods before they were used. Analytical thin-layer chromatography (TLC) was performed on Merck silica gel aluminum plates with an F-254 indicator, visualized by irradiation with UV light. Flash chromatography columns were packed with 200–300 mesh silica gel, which was purchased from Qing Dao Hai Yang Chemical Industry. The Digital Single-Channel Adjustable Automatic Electronic Pipette Micropipette dPettee+ was purchased from Dragon Laboratory Instruments Limited (DLAB Scientific Co., Ltd, Beijing, China). ^1^H NMR and ^13^C NMR spectra were recorded on a Bruker DPX-400 spectrometer (Brooke Technologies Co., Ltd., Zurich, Switzerland) in CDCl_3_. All chemical shifts (*δ*) are reported in ppm and coupling constants (*J*) in Hz relative to tetramethylsilane as an internal standard (*δ* = 0 ppm). For the ^19^F spectra, *α*-trifluorotoluene served as an external standard (*δ* = −63.9 ppm). High-resolution mass spectra (HRMS) were obtained on a Waters UPLC G2-XS Qtof spectrometer (Waters World Science & Technology Co., Ltd., Wilmslow, UK) using electrospray ionization (ESI). Low-resolution mass spectrometry (LRMS) was performed using an Agilent 1260–6120 (Agilent Technologies Co., Ltd, California, USA) single quadrupole LC-MS using electrospray ionization (ESI). Cyclic voltammetry (CV) was recorded in MeCN by CHI1040C (Shanghai, China). Electrolysis was conducted using an IKA ElectraSyn 2.0 (Germany) in the constant current mode. The X-ray single-crystal structure was determined by a GeminiE X-ray single-crystal diffractometer (Oxford Diffraction Limited, Oxford, UK).

### 3.2. Cyclic Voltammetry Experiments

Cyclic voltammetry (CV) was measured in a glass cell with a CHI1040C electrochemical workstation under an Ar atmosphere with a conventional three-electrode system. The working electrode was a steady glassy carbon disk electrode, and the counter electrode was a platinum wire. The reference was an Ag/AgCl electrode submerged in a saturated aqueous KCl solution. A total of 6 mL of CH_3_CN containing 0.05 M *n*-Bu_4_NPF_6_ was poured into the electrochemical cell in all experiments. The CV of substrates (**1a** and **2a**) was measured at a concentration of 10 mM. The scan rate was 0.1 V/s, ranging from 0 V to 3 V.

### 3.3. General Procedure for the Electrochemical Synthesis of 1-(Isochroman-1-yl)-1H-Indazole

Compounds **1** and **2**, acid, electrolyte and solvent were added to the ElectraSyn reaction vial equipped with an anode and a cathode. The doses of all reagents, additives and solvents are given in the manuscript. The reaction mixture was electrolyzed under the indicated constant current. After the reaction, the electrodes were rinsed with ethyl acetate (15 mL), which was combined with the reaction mixture. The reaction mixture was washed with 20 mL saturated saline water and then extracted with ethyl acetate (3 × 15 mL). The organic layer was dried by Na_2_SO_4_ and concentrated under reduced pressure. The residue was purified by silica gel column chromatography (ethyl acetate/petroleum ether = 1:25 to 1:40, *v/v*) to produce the desired products **3**.

*1-(isochroman-1-yl)-1H-indazole* (**3a**). Colorless oil (66.2 mg, 88%). ^1^H NMR (400 MHz, CDCl_3_): *δ* 8.04 (s, 1H), 7.73 (d, *J* = 8.1 Hz, 1H), 7.36–7.24 (m, 3H), 7.23–7.07 (m, 4H), 6.97 (d, *J* = 7.8 Hz, 1H), 4.20–3.93 (m, 2H), 3.13–2.90 (m, 2H). ^13^C NMR (100 MHz, CDCl_3_ & MeOD (v:v = 30:1)): *δ* 139.9, 134.8, 134.7, 131.7, 128.9, 128.5, 126.9, 126.9, 126.7, 124.9, 121.4, 121.1, 110.4, 83.5, 81.8, 28.0. HRMS (ESI), calcd. for C_16_H_14_N_2_NaO (M + Na)^+^: 273.0998, found: 273.0992 [36,40].

*1-(isochroman-1-yl)-4-methoxy-1H-indazole* (**3b**). Colorless oil (52.9 mg, 63%). ^1^H NMR (400 MHz, CDCl_3_): *δ* 8.11 (s, 1H), 7.35–7.26 (m, 2H), 7.24–7.14 (m, 2H), 7.07 (s, 1H), 6.96 (d, *J* = 7.7 Hz, 1H), 6.78 (d, *J* = 8.4, 1H), 6.49 (d, *J* = 7.7 Hz, 1H), 4.19–3.97 (m, 2H), 3.97 (s, 3H), 3.13–2.91 (m, 2H). ^13^C NMR (100 MHz, CDCl_3_): *δ* 153.8, 141.9, 134.8, 132.5, 132.0, 129.0, 128.5, 128.0, 127.0, 126.6, 116.8, 103.1, 100.3, 83.4, 61.6, 55.4, 28.1. HRMS (ESI), calcd. for C_17_H_16_N_2_NaO_2_ (M + Na)^+^: 303.1104, found: 303.1108.

*1-(isochroman-1-yl)-4-methyl-1H-indazole* (**3c**). Colorless oil (78.4 mg, 99%). ^1^H NMR (400 MHz, CDCl_3_): *δ* 8.06 (s, 1H), 7.35–7.26 (m, 2H), 7.23–7.14 (m, 2H), 7.10 (s, 1H), 7.02 (d, *J* = 8.4 Hz, 1H), 6.99–6.91 (m, 2H), 4.22–3.92 (m, 2H), 3.17–2.87 (m, 2H), 2.59 (s, 3H). ^13^C NMR (100 MHz, CDCl_3_): *δ* 140.0, 134.9, 133.5, 132.0, 131.6, 129.0, 128.5, 127.0, 126.8, 126.7, 125.4, 121.3, 107.8, 83.4, 61.6, 28.1, 18.6. HRMS (ESI), calcd. for C_17_H_16_N_2_NaO (M + Na)^+^: 287.1155, found: 287.1161.

*4-fluoro-1-(isochroman-1-yl)-1H-indazole* (**3d**). Colorless oil (79.6 mg, 99%). ^1^H NMR (400 MHz, CDCl_3_): *δ* 8.11 (s, 1H), 7.41–7.14 (m, 4H), 7.10 (s, 1H), 7.01–6.91 (m, 2H), 6.85–6.75 (d, *J* = 8.4 Hz, 1H), 4.20–3.97 (m, 2H), 3.16–2.90 (m, 2H). ^13^C NMR (100 MHz, CDCl_3_): *δ* 155.9 (d, *J* = 252.4 Hz), 142.6 (d, *J* = 8.1 Hz), 134.8, 131.6, 131.0 (d, *J* = 2.2 Hz), 129.0, 128.7, 127.7 (d, *J* = 7.3 Hz), 127.0, 126.7, 115.3 (d, *J* = 22.7 Hz), 106.5 (d, *J* = 4.4 Hz), 105.7 (d, *J* = 18.3 Hz), 83.7, 61.7, 28.0. ^19^F NMR (376 MHz, CDCl_3_): *δ* −118.0. HRMS (ESI), calcd. for C_16_H_13_FN_2_NaO (M + Na)^+^: 291.0904, found: 291.0907.

*4-chloro-1-(isochroman-1-yl)-1H-indazole* (**3e**). Colorless oil (84.4 mg, 99%). ^1^H NMR (400 MHz, CDCl_3_): *δ* 8.12 (s, 1H), 7.36–7.25 (m, 2H), 7.23–7.05 (m, 5H), 6.94 (d, *J* = 7.7 Hz, 1H), 4.17–3.94 (m, 2H), 3.14–2.88 (m, 2H). ^13^C NMR (100 MHz, CDCl_3_): *δ* 141.0, 134.8, 133.2, 131.6, 129.1, 128.7, 127.3, 127.0, 126.8, 126.7, 124.3, 121.0, 109.1, 83.8, 61.7, 28.0. HRMS (ESI), calcd. for C_16_H_13_ClN_2_NaO (M + Na)^+^: 307.0609, found: 307.0619.

*1-(isochroman-1-yl)-5-methoxy-1H-indazole* (**3f**). Colorless oil (63.8 mg, 76%). ^1^H NMR (400 MHz, CDCl_3_): *δ* 7.95 (s, 1H), 7.35–7.26 (m, 2H), 7.21–7.15 (m, 1H), 7.09–6.93 (m, 5H), 4.17–3.95 (m, 2H), 3.83 (s, 3H), 3.13–2.92 (m, 2H). ^13^C NMR (100 MHz, CDCl_3_): *δ* 155.0, 135.8, 134.9, 134.0, 132.0, 129.0, 128.5, 127.0, 126.7, 125.5, 118.7, 111.4, 100.4, 83.7, 61.7, 55.7, 28.1. HRMS (ESI), calcd. for C_17_H_16_N_2_NaO_2_ (M + Na)^+^: 303.1104, found: 303.1095.

*1-(isochroman-1-yl)-5-methyl-1H-indazole* (**3g**). Colorless oil (78.4 mg, 99%). ^1^H NMR (400 MHz, CDCl_3_): *δ* 7.95 (s, 1H), 7.49 (s, 1H), 7.35–7.26 (m, 2H), 7.21–7.14 (m, 1H), 7.14–7.01 (m, 3H), 6.98 (d, *J* = 7.7 Hz, 1H), 4.21–3.92 (m, 2H), 3.14–2.91 (m, 2H), 2.43 (s, 3H). ^13^C NMR (100 MHz, CDCl_3_): *δ* 138.7, 134.9, 134.2, 132.0, 130.8, 129.0, 128.7, 128.5, 127.0, 126.7, 125.5, 120.2, 110.0, 83.5, 61.6, 28.1, 21.3. HRMS (ESI), calcd. for C_17_H_16_N_2_NaO (M + Na)^+^: 287.1155, found: 287.1159.

*5-fluoro-1-(isochroman-1-yl)-1H-indazole* (**3h**). Colorless oil (79.6 mg, 99%). ^1^H NMR (400 MHz, CDCl_3_): *δ* 8.00 (s, 1H), 7.39–7.26 (m, 3H), 2.23–7.14 (m, 1H), 7.14–7.01 (m, 3H), 6.96 (d, *J* = 7.7 Hz, 1H), 4.20–3.91 (m, 2H), 3.15–2.90 (m, 2H). ^13^C NMR (100 MHz, CDCl_3_): *δ* 158.1 (d, *J* = 239.2 Hz), 136.8, 134.8, 134.3 (d, *J* = 5.1 Hz), 131.7, 129.1, 128.7, 126.9, 126.7, 125.2 (d, *J* = 9.5 Hz), 116.2 (d, *J* = 27.1 Hz), 111.6 (d, *J* = 9.5 Hz), 105.2 (d, *J* = 23.5 Hz), 83.9, 61.8, 28.1. ^19^F NMR (376 MHz, CDCl_3_): *δ* -122.4. HRMS (ESI), calcd. for C_16_H_13_FN_2_NaO (M + Na)^+^: 291.0904, found: 291.0911.

*5-bromo-1-(isochroman-1-yl)-1H-indazole* (**3i**). Colorless oil (85.3 mg, 87%). ^1^H NMR (400 MHz, CDCl_3_): *δ* 7.98 (s, 1H), 7.87 (s, 1H), 7.40–7.24 (m, 3H), 7.23–7.14 (m, 1H), 7.11 (s, 1H), 7.03 (d, *J* = 8.9 Hz, 1H), 6.95 (d, *J* = 7.7 Hz, 1H), 4.16–3.95 (s, 2H), 3.15–2.88 (m, 2H). ^13^C NMR (100 MHz, CDCl_3_): *δ* 138.6, 134.8, 133.8, 131.5, 129.8, 129.1, 128.7, 126.9, 126.8, 126.6, 123.6, 114.4, 111.9, 83.8, 61.8, 28.0. HRMS (ESI), calcd. for C_16_H_13_BrN_2_NaO (M + Na)^+^: 351.0103, found: 351.0110.

*1-(isochroman-1-yl)-5-nitro-1H-indazole* (**3j**). Colorless oil (50.3 mg, 57%). ^1^H NMR (400 MHz, CDCl_3_): *δ* 8.72 (s, 1H), 8.24 (s, 1H), 8.21–8.14 (m, 1H), 7.42–7.28 (m, 2H), 7.25–7.17 (m, 2H), 7.14 (s, 1H), 6.94 (d, *J* = 7.8 Hz, 1H), 4.22–3.98 (m, 2H), 3.17–2.94 (m, 2H). ^13^C NMR (100 MHz, CDCl_3_): *δ* 142.8, 141.7, 136.7, 134.8, 131.0, 129.2, 129.0, 126.9, 126.8, 124.3, 121.7, 118.7, 110.9, 84.2, 61.9, 27.9. HRMS (ESI), calcd. for C_16_H_13_N_3_NaO_3_ (M + Na)^+^: 318.0849, found: 318.0855 [36].

*1-(isochroman-1-yl)-6-methoxy-1H-indazole* (**3k**). Colorless oil (73.4 mg, 87%). ^1^H NMR (400 MHz, CDCl_3_): *δ* 7.93 (s, 1H), 7.57 (d, *J* = 8.8 Hz, 1H), 7.36–7.27 (m, 2H), 7.20 (m, 1H), 7.05 (s, 1H), 7.01 (d, *J* = 7.7 Hz, 1H), 6.84–6.78 (m, 1H), 6.53 (s, 1H), 4.19–3.97 (m, 2H), 3.73 (s, 3H), 3.15–2.91 (m, 2H). ^13^C NMR (100 MHz, CDCl_3_): *δ* 159.4, 141.4, 134.9, 134.7, 131.9, 129.0, 128.5, 127.2, 126.7, 121.8, 119.6, 113.3, 91.7, 83.2, 61.4, 55.4, 28.1. HRMS (ESI), calcd. for C_17_H_16_N_2_NaO_2_ (M + Na)^+^: 303.1104, found: 303.1113.

*1-(isochroman-1-yl)-6-methyl-1H-indazole* (**3l**). Colorless oil (65.9 mg, 83%). ^1^H NMR (400 MHz, CDCl_3_): *δ* 7.96 (s, 1H), 7.60 (d, *J* = 8.2 Hz, 1H). 7.36–7.26 (m, 2H), 7.22–7.14 (m, 1H), 7.07 (s, 2H), 7.04–6.93 (m, 2H), 4.21–3.93 (m, 2H), 3.19–2.90 (m, 2H), 2.43 (s, 3H). ^13^C NMR (100 MHz, CDCl_3_): *δ* 140.7, 137.0, 134.8, 134.5, 132.1, 129.0, 128.4, 127.0, 126.6, 123.6, 123.2, 120.7, 109.7, 82.9, 61.5, 28.1, 22.1. HRMS (ESI), calcd. for C_17_H_16_N_2_NaO (M + Na)^+^: 287.1155, found: 287.1157.

*6-fluoro-1-(isochroman-1-yl)-1H-indazole* (**3m**). Colorless oil (77.6 mg, 96%). ^1^H NMR (400 MHz, CDCl_3_): *δ* 8.01 (s, 1H), 7.70–7.61 (m, 1H), 7.38–7.27 (m, 2H), 7.23–7.15 (m, 1H), 7.04 (s, 1H), 7.00–6.88 (m, 2H), 6.77 (d, *J* = 8.4 Hz, 1H), 4.20–3.95 (m, 2H), 3.15–2.91 (m, 2H). ^13^C NMR (100 MHz, CDCl_3_): *δ* 162.1 (d, *J* = 245.0 Hz), 140.3 (d, *J* = 12.5 Hz), 134.8, 134.8, 131.5, 129.1, 128.7, 126.9, 126.8, 122.4 (d, *J* = 11.0 Hz). 121.9, 111.2 (d, *J* = 25.7 Hz), 96.6 (d, *J* = 27.1 Hz), 83.9, 61.8, 28.0. ^19^F NMR (376 MHz, CDCl_3_): *δ* -113.6. HRMS (ESI), calcd. for C_16_H_13_FN_2_NaO (M + Na)^+^: 291.0904, found: 291.0913.

*6-chloro-1-(isochroman-1-yl)-1H-indazole* (**3n**). Colorless oil (84.4 mg, 99%). ^1^H NMR (400 MHz, CDCl_3_): *δ* 8.00 (s, 1H), 7.64 (d, *J* = 8.6 Hz, 1H), 7.40–7.27 (m, 2H), 7.24–7.08 (m, 3H), 7.05 (s, 1H), 6.96 (d, *J* = 7.7 Hz, 1H), 4.19–3.86 (m, 2H), 3.14–2.90 (m, 2H). ^13^C NMR (100 MHz, CDCl_3_): *δ* 140.4, 134.8, 134.6, 133.1, 131.5, 129.1, 128.7, 126.9, 126.8, 123.6, 122.5, 122.0, 110.3, 83.6, 61.7, 28.0. HRMS (ESI), calcd. for C_16_H_13_ClN_2_NaO (M + Na)^+^: 307.0609, found: 307.0609.

*1-(isochroman-1-yl)-6-nitro-1H-indazole* (**3o**). Colorless oil (47.1 mg, 60%). ^1^H NMR (400 MHz, CDCl_3_): *δ* 8.16 (s, 2H), 8.09–8.00 (m, 1H), 7.90–7.79 (d, *J* = 8.9 Hz, 1H), 7.42–7.31 (m, 2H), 7.24–7.15 (m, 2H), 6.96 (d, *J* = 7.8 Hz, 1H), 4.15–3.99 (m, 2H), 3.18–2.96 (m, 2H). ^13^C NMR (100 MHz, CDCl_3_): *δ* 146.7, 138.7, 134.8, 134.6, 130.9, 129.3, 129.1, 128.2, 126.9, 126.8, 121.8, 116.3, 107.3, 84.1, 61.9, 27.9. HRMS (ESI), calcd. for C_16_H_13_N_3_NaO_3_ (M + Na)^+^: 318.0849, found: 318.0849.

*1-(isochroman-1-yl)-7-methyl-1H-indazole* (**3p**). Colorless oil (56.9 mg, 72%). ^1^H NMR (400 MHz, CDCl_3_): *δ* 7.96 (s, 1H), 7.59 (d, *J* = 8.0 Hz, 1H), 7.37–7.26 (m, 3H), 7.24–7.17 (m, 2H), 7.15–7.09 (m, 1H), 7.04 (d, *J* = 7.5 Hz, 1H), 4.17–3.85 (m, 2H), 3.22–3.03 (m, 1H), 2.98–2.73 (m, 4H). ^13^C NMR (100 MHz, CDCl_3_): *δ* 140.0, 134.6, 134.6, 132.7, 129.0, 129.0, 128.2, 127.2, 126.3, 125.5, 121.6, 121.0, 119.0, 82.6, 60.2, 28.1, 19.7. HRMS (ESI), calcd. for C_17_H_16_N_2_NaO (M + Na)^+^: 287.1155, found: 287.1159.

*1-(isochroman-1-yl)-3-methyl-1H-indazole* (**3r**). Colorless oil (78.4 mg, 99%). ^1^H NMR (400 MHz, CDCl_3_): *δ* 7.63 (d, *J* = 8.0 Hz, 1H), 7.34–7.26 (m, 2H), 7.21–7.13 (m, 2H), 7.13–7.08 (m, 1H), 7.05 (s, 1H), 7.01 (d, *J* = 7.7 Hz, 1H), 6.82 (d, *J* = 8.4 Hz, 1H), 4.22–3.98 (m, 2H), 3.08–2.96 (m, 2H), 2.57 (s, 3H). ^13^C NMR (100 MHz, CDCl_3_): *δ* 143.5, 140.8, 135.0, 132.3, 129.0, 128.4, 127.1, 126.8, 126.6, 124.9, 120.5, 120.4, 110.6, 84.2, 62.3, 28.2, 12.1. HRMS (ESI), calcd. for C_17_H_16_N_2_NaO (M + Na)^+^: 287.1155, found: 287.1158.

*1-(isochroman-1-yl)-1H-benzo[d]imidazole* (**3s**). Colorless oil (11.1mg, 15%). ^1^H NMR (400 MHz, CDCl_3_): *δ* 7.84–7.78 (m, 1H), 7.61 (s, 1H), 7.48–7.41 (m, 1H), 7.41–7.35 (m, 1H), 7.34–7.21 (m, 4H), 7.07 (d, *J* = 7.7 Hz, 1H), 6.91 (s, 1H), 4.07–3.71 (m, 2H), 3.21–2.78 (m, 2H). ^13^C NMR (100 MHz, CDCl_3_): *δ* 144.2, 142.7, 135.0, 133.8, 130.3, 129.3, 129.2, 127.1, 127.0, 123.4, 122.8, 120.4, 111.1, 80.2, 60.1, 27.7. HRMS (ESI), calcd. for C_16_H_14_N_2_NaO (M + Na)^+^: 273.0998, found: 273.1003 [36].

*1-(isochroman-1-yl)-1H-benzo[d][1,2,3]triazole* (**3t**). Colorless oil (33.1 mg, 44%). ^1^H NMR (400 MHz, CDCl_3_): *δ* 8.12–8.00 (m, 1H), 7.42 (s, 1H), 7.40–7.28 (m, 4H), 7.23–7.15 (m, 1H),7.05–6.97 (m, 1H), 6.94 (d, *J* = 7.8 Hz, 1H), 4.17–4.03 (m, 2H), 3.22–2.95 (m, 2H). ^13^C NMR (100 MHz, CDCl_3_ & MeOD (v:v = 30:1)): *δ* 146.1, 134.7, 132.6, 129.9, 129.1, 129.1, 127.8, 127.0, 126.8, 124.4, 119.7, 110.8, 83.5, 62.1, 27.7. HRMS (ESI), calcd. for C_15_H_13_N_3_NaO (M + Na)^+^: 274.0951, found: 274.0956 [36].

*1-(isochroman-1-yl)-3-phenyl-1H-pyrazole* (**3u**). Colorless oil (34.5 mg, 42%). ^1^H NMR (400 MHz, CDCl_3_): *δ* 7.86 (d, *J* = 7.1 Hz, 2H), 7.43–7.35 (m, 2H), 7.35–7.28 (m, 2H), 7.28–7.15 (m, 3H), 7.10 (d, *J* = 7.7 Hz, 1H), 6.85 (s, 1H), 6.55 (s, 1H), 4.09–3.89 (m, 2H), 3.16–2.97 (m, 1H), 2.90–2.76 (m, 1H). ^13^C NMR (100 MHz, CDCl_3_): *δ* 152.5, 135.0, 133.3, 131.2, 130.7, 129.0, 128.8, 128.6, 127.9, 127.5, 126.7, 125.9, 103.2, 85.0, 60.4, 27.9. HRMS (ESI), calcd. for C_18_H_16_N_2_NaO (M + Na)^+^: 299.1155, found: 299.1159 [36].

*1-(isochroman-1-yl)-5-methyl-3-phenyl-1H-pyrazole* (**3v**). Colorless oil (48.3 mg, 58%). ^1^H NMR (400 MHz, CDCl_3_): *δ* 7.75 (d, *J* = 7.2 Hz, 2H), 7.38–7.31 (m, 2H), 7.29–7.23 (m, 2H), 7.22–7.12 (m, 2H), 6.91 (d, *J* = 7.7 Hz, 1H), 6.81 (s, 1H), 6.39 (s, 1H), 4.40–4.30 (m, 1H), 4.07–3.98 (m, 1H), 3.12–3.01 (m, 1H), 2.95–2.83 (m, 1H), 2.15 (s, 3H). ^13^C NMR (100 MHz, CDCl_3_): *δ* 150.6, 140.7, 134.6, 133.6, 133.1, 128.7, 128.5, 128.1, 127.6, 126.6, 126.3, 125.7, 104.6, 85.0, 62.9, 28.1, 11.6. HRMS (ESI), calcd. for C_19_H_18_N_2_NaO (M + Na)^+^: 313.1311, found: 313.1319.

*1-(7-bromoisochroman-1-yl)-1H-indazole* (**3w**). Colorless oil (44.5 mg, 45%). ^1^H NMR (400 MHz, CDCl_3_): *δ* 8.05(s, 1H), 7.76 (d, *J* = 8.1 Hz, 1H), 7.48–7.41 (m, 1H), 7.40–7.30 (m, 2H), 7.24–7.10 (m, 3H), 7.05 (s, 1H), 4.18–4.09 (m, 1H), 4.03–3.94 (m, 1H), 3.07–2.86 (m, 2H). ^13^C NMR (100 MHz, CDCl_3_): *δ* 140.1, 135.0, 134.0, 133.9, 131.7, 130.7, 129.9, 126.9, 125.0, 121.6, 121.3, 120.1, 110.0, 82.2, 61.3, 27.6. HRMS (ESI), calcd. for C_16_H_14_BrN_2_O (M + H)^+^: 329.0284, found: 329.0285.

*1-(tetrahydrofuran-2-yl)-1H-indazole* (**3x**). Colorless oil (34.9 mg, 62%). ^1^H NMR (400 MHz, CDCl_3_): *δ* 8.01 (s, 1H), 7.71 (d, *J* = 8.0 Hz, 1H), 7.63–7.55 (m, 1H), 7.43–7.30 (m, 1H), 7.21–7.07 (m, 1H), 6.44–6.28 (m, 1H), 4.11–3.89 (m, 2H), 3.00–2.83 (m, 1H), 2.48–2.31 (m, 2H), 2.17–1.98 (m, 1H), ^13^C NMR (100 MHz, CDCl_3_): *δ* 139.9, 133.9, 126.6, 124.7, 121.1, 121.0, 109.7, 86.9, 68.8, 30.3, 25.1. HRMS (ESI), calcd. for C_11_H_12_N_2_NaO (M + Na)^+^: 211.0842, found: 211.0850 [40].

*1-(tetrahydro-2H-pyran-2-yl)-1H-indazole* (**3y**). Colorless oil (31.8 mg, 52%). ^1^H NMR (400 MHz, CDCl_3_): *δ* 8.03 (s, 1H), 7.72 (d, *J* = 8.2 Hz, 1H), 7.59 (d, *J* = 8.6 Hz, 1H), 7.43–7.35 (m, 1H), 7.21–7.13 (m, 1H), 5.79–5.66 (m, 1H), 4.12–3.97 (m, 1H), 3.85–3.67 (m, 1H), 2.67–2.50 (m, 1H), 2.23–2.03 (m, 2H), 1.89–1.51 (m, 3H). ^13^C NMR (100 MHz, CDCl_3_): *δ* 139.5, 134.0, 126.6, 124.7, 121.2, 121.1, 110.0, 85.3, 67.6, 29.5, 25.2, 22.7. HRMS (ESI), calcd. for C_12_H_14_N_2_NaO (M + Na)^+^: 225.0998, found: 225.1005.

*1-(1,3-dihydroisobenzofuran-1-yl)-1H-indazole* (**3z**). Colorless oil (29.7 mg, 42%). ^1^H NMR (400 MHz, CDCl_3_): *δ* 8.05 (s, 1H), 7.71 (d, *J* = 8.1 Hz, 1H), 7.60 (s, 1H), 7.51–7.40 (m, 2H), 7.38–7.31 (m, 1H), 7.29–7.20 (m, 2H), 7.18–7.08 (m, 1H), 6.96–6.86 (m, 1H), 5.43–5.4 (m, 1H), 5.25 (d, *J* = 12.6 Hz, 1H). ^13^C NMR (100 MHz, CDCl_3_): *δ* 139.9, 139.3, 135.8, 135.0, 129.7, 128.2, 126.7, 125.5, 123.2, 121.4, 121.4, 121.2, 109.8, 92.7, 73.3. HRMS (ESI), calcd. for C_15_H_12_N_2_NaO (M + Na)^+^: 259.0842, found: 259.0844.

## 4. Conclusions

In summary, we developed an efficient and simple electro-oxidative method for α-C(sp^3^)–H/N–H cross-coupling reactions to achieve isochroman–azole hybrid molecules. Under metal- and oxidant-free conditions, various substituted indazoles and isochromans were suitable for this transformation with moderate to good yields at room temperature in air. Based on the results of control experiments and cyclic voltammetry, the reaction might involve both radical coupling and nucleophilic addition processes. This work provides another example of a new strategy for the efficient and mild synthesis of hybrid molecules.

## Figures and Tables

**Table 1:**
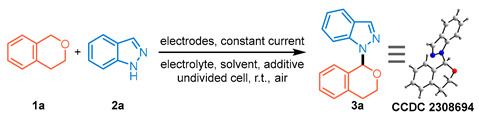
Optimization of the reaction conditions ^a^.

Entry	Anode/Cathode	Electrolyte	Additive	Yield (%) ^b^
1	RVC/Ni	*n*-Bu_4_NBF_4_	-	trace
2	RVC/Ni	*n*-Bu_4_NBF_4_	TfOH	39
3	RVC/Ni	*n*-Bu_4_NBF_4_	AcOH	2
4 ^c^	RVC/Ni	*n*-Bu_4_NBF_4_	TfOH	73
5 ^c^	RVC/Ni	*n*-Bu_4_NClO_4_	TfOH	54
6 ^c^	RVC/Ni	*n*-Bu_4_NPF_6_	TfOH	88
7 ^c^	RVC/Ni	*n*-Bu_4_NOAc	TfOH	39
8 ^c^	C/Ni	*n*-Bu_4_NPF_6_	TfOH	73
9 ^c^	RVC/Pt	*n*-Bu_4_NPF_6_	TfOH	71
10 ^c,d^	RVC/Ni	*n*-Bu_4_NPF_6_	TfOH	50
11 ^c,e,f^	RVC/Ni	*n*-Bu_4_NPF_6_	TfOH	72
12 ^c,g^	RVC/Ni	*n*-Bu_4_NPF_6_	TfOH	88
13 ^c,h^	RVC/Ni	*n*-Bu_4_NPF_6_	TfOH	69
14 ^c,f^	RVC/Ni	*n*-Bu_4_NPF_6_	TfOH	84
15 ^c^	RVC/Ni	*n*-Bu_4_NPF_6_	-	70
16 ^c,i^	RVC/Ni	*n*-Bu_4_NPF_6_	TfOH	50

^a^ Reaction conditions: **1a** (0.3 mmol), **2a** (0.45 mmol, 1.5 equiv.), I = 10 mA, electrolyte (0.1 M), additive (0.45 mmol), MeCN (6 mL), at room temperature for 3 h. ^b^ Isolated yield. ^c^ **1a** (1.2 mmol), **2a** (0.3 mmol). ^d^ EtOH was used as solvent. ^e^ 7.5 mA. ^f^ 5 h. ^g^ 12.5 mA. ^h^ 2.5 h. ^i^ Ar.

## Data Availability

The data presented in this study are available in the article and Supplementary Materials.

## Supplementary Materials

The following supporting information can be downloaded at https://www.mdpi.com/article/10.3390/molecules30010004/s1, Copies of ^1^H NMR, ^13^C NMR and ^19^F NMR spectra of the products are included in the Supporting Information. Figure S1–S4: Cyclic voltammetry of isochroman and indazole. Figures S5–S8: LC-MS spectrum of compounds. Figures S9–S61: ^1^H and ^13^C NMR spectra of products. Figures S62–S86: HRMS Spectra for the Products. Scheme S1: Control experiments. Table S1 and Figure S87: Crystal data and structure refinement for **3a**.
