# Supplementary material for: Electrochemical α-C(sp3)–H/N–H Cross-Coupling of Isochromans and Azoles
Source: Molecules. 2024 Dec 24;30(1):4. doi: 10.3390/molecules30010004 (PMC11720983; doi:10.3390/molecules30010004)
Supplement: Supplementary file 1 [file molecules-30-00004-s001.zip › molecules-3347859-supplementary.pdf]

Supporting Information

Electrochemical α-C(sp^3)–H/N–H Cross-Coupling of Isochromans and Azoles

Guoping Li, Bing Yan, Liangliang Wu, Yabo Li, Xinqi Hao, Ming Gong * and Yangjie Wu

College of Chemistry, Henan Key Laboratory of Chemical Biology and Organic Chemistry, Key Laboratory of Applied Chemistry of Henan Universities, Zhengzhou University, Zhengzhou 450052, China.

*Corresponding authors. E-mail: gongming@zzu.edu.cn

Table of Contents

1. General Information 3

2. Experimental Procedures 3

3. Cyclic Voltammetry Experiments 4

4. Control Experiments 6

5. ^{1}H, ^{13}C and ^{19}F NMR Spectra 8

6. HRMS Spectra for the Products 35

7. Determination of Structure of 3a 48

1. General Information

All reagents were obtained from commercial sources without further purification unless otherwise stated. Solvents used in all experiments were dried and degassed by standard methods. Analytical thin-layer chromatography (TLC) was performed on Merck silica gel aluminum plates with F-254 indicator, visualized by irradiation with UV light. Flash chromatography columns were packed with 200-300 mesh silica gel which was purchased from Qing Dao Hai Yang Chemical Industry. Digital Single Channel Adjustable Automatic Electronic Pipette Micropipette dPettee+ were purchased from Dragon Laboratory Instruments Limited. ^{1}H NMR and ^{13}C NMR spectra were recorded on a Bruker DPX-400 spectrometer in $CDCl_3$. All chemical shifts (δ) are reported in ppm and coupling constants (J) in Hz relative to tetramethylsilane as internal standard (δ = 0 ppm). For the ^{19}F spectra, α-trifluorotoluene served as external standard (δ = -63.9 ppm). High resolution mass spectra (HRMS) were obtained on a waters UPLC G2-XS Qtof spectrometer using electrospray ionization (ESI). Low resolution mass spectrometry (LRMS) was obtained on Agilent 1260-6120 single quadrupole LC-MS using electrospray ionization (ESI). The cyclic voltammetry (CV) was recorded in CH_3CN by CHI1040C. Electrolysis was conducted using an IKA Electra 2.0 in constant current mode. The X-ray single crystal structure is determined by GeminiE X-ray single crystal diffractometer.

2. Experimental Procedures

General procedure for the electrochemical synthesis of 1-(isochroman-1-yl)-1*H*-indazole

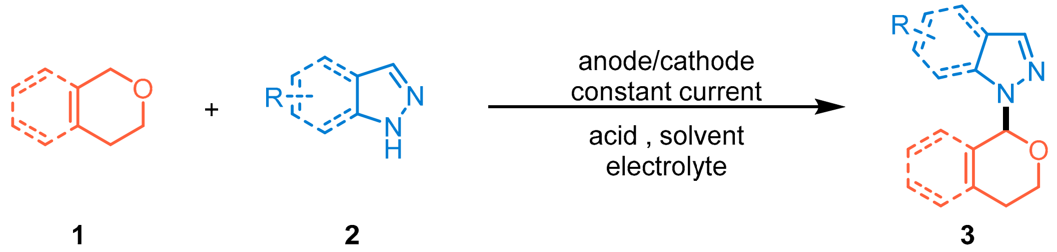

Compounds **1**, compounds **2**, acid, electrolyte and solvent were added to the ElectraSyn reaction vial equipped with an anode and a cathode. The doses of all reagents, additives and solvents are given in the manuscript. The reaction mixture was electrolyzed under the indicated constant current. After the reaction, the electrodes were rinsed with ethyl acetate (15 mL), which was combined with the reaction mixture. The reaction mixture was washed with 20 mL saturated saline water, then extracted with ethyl acetate (3×15 mL). The organic layer was dried by Na_2SO_4, and concentrated under reduced pressure. The residue was purified by silica gel column chromatography (ethyl acetate/petroleum

ether = 1:25 to 1:40, v/v) to give the desired products **3**.

3. Cyclic Voltammetry Experiments

Cyclic voltammetry (CV) was measured in a glass cell with CHI1040C electrochemical workstation under Ar atmosphere with a conventional three-electrode system. The working electrode was a steady glassy carbon disk electrode, and the counter electrode was a platinum wire. The reference was an Ag/AgCl electrode submerged in the saturated aqueous KCl solution. 6 mL of CH_3CN containing 0.05 M *n*-Bu_4NPF_6 were poured into the electrochemical cell in all experiments. The CV of substrates (**1a** and **2a**) were measured at the concentration of 10 mM. The scan rate was 0.1 V/s, ranging from 0 V to 3 V.

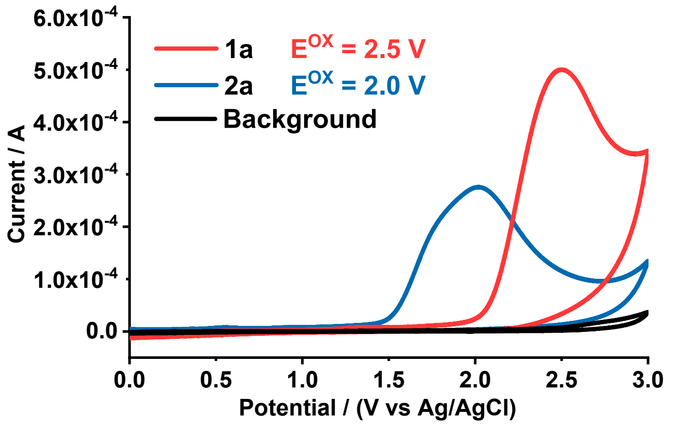

Figure S1. Cyclic voltammetry of isochroman (**1a**) and indazole (**2a**) was measured in 0.05 M *n*-Bu_4NPF_6/MeCN using a GC disk working, Pt wire and Ag/AgCl as the counter and reference electrode at 100 mV/s scan rate.

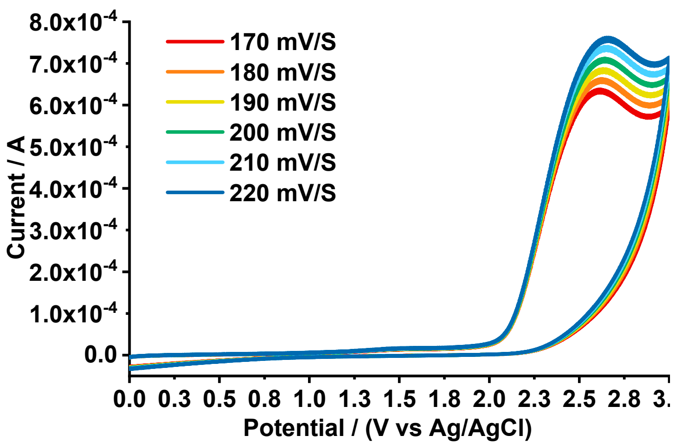

Figure S2. Cyclic voltammograms of isochroman (**1a**) at different scan rates. Curves were obtained at 170, 180, 190, 200, 210, 220 mV/s, respectively.

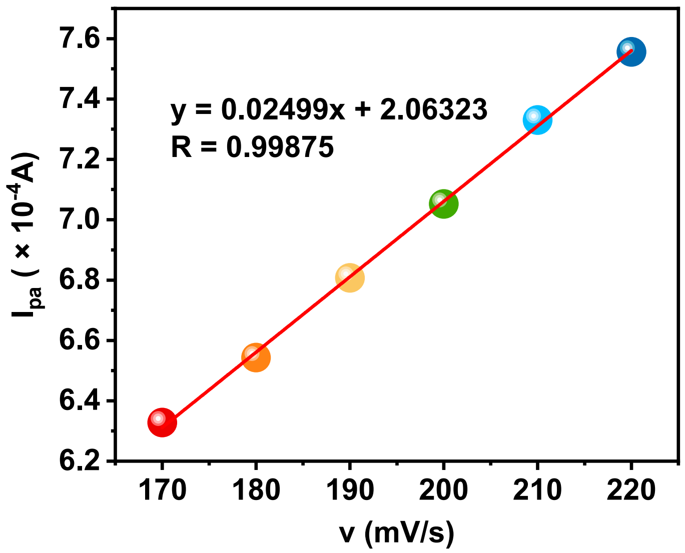

Figure S3. The plot of peak current *vs* scan rate for isochroman (**1a**).

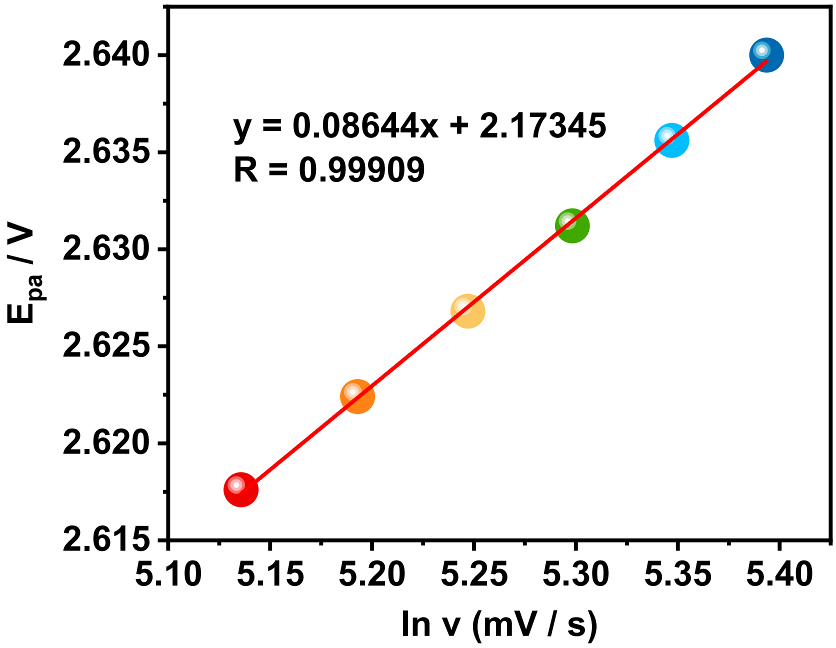

Figure S4. The relationship between E_{pa} and lnv for isochroman (**1a**).

The peak current increased linearly with the scan rate in the range of 170-220 mV/s and the equation could be expressed as follows: y = 0.02499 x + 2.06323, R = 0.99875. It could be seen that the oxidation of compound **1a** was an absorption-controlled process. For an adsorption-controlled and irreversible electrode process, according to Laviron method, [39] E_{pa} is defined by the following equation: $E_{pa} = E^0 + (RT/\alpha nF) \ln (RTk^0/\alpha nF) + (RT/\alpha nF) \ln v$.

Where α is transfer coefficient, k^0 is standard rate constant of the reaction, n is electron transfer number involved in the rate-determining step, v is scan rate, and E^0 is formal potential. Other symbols have their usual meanings. Thus, the value of α can be easily calculated from the slope of E_{pa}-lnv. In this system, the slope is 0.08644. Generally, transfer coefficient α was assumed as 0.5, so the value of the number of electron (n)

was calculated to be 1.

4. Control Experiments

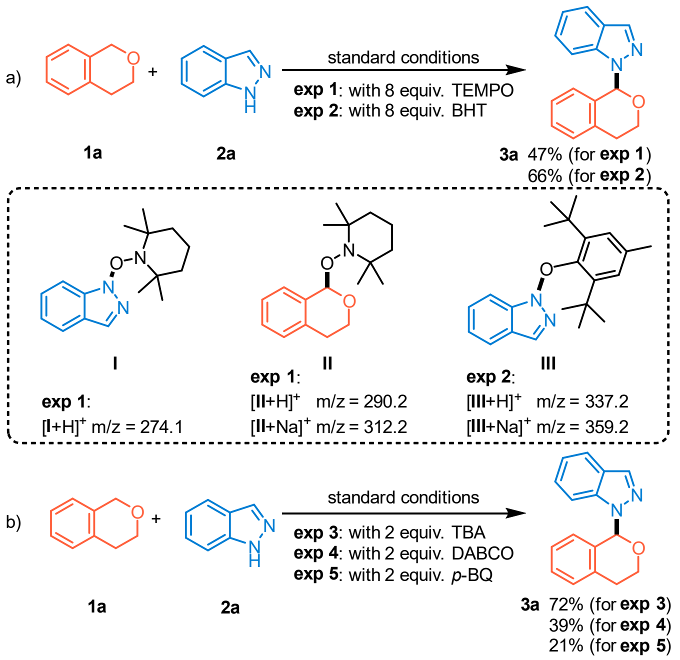

Scheme S1. Control experiments

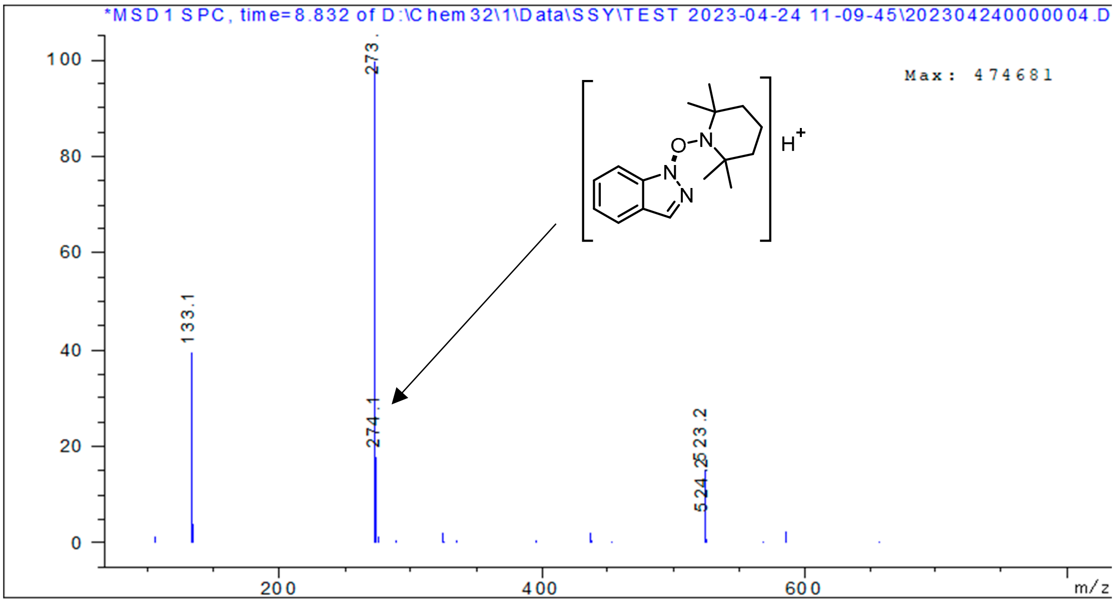

Figure S5. LC-MS spectrum of [**I** + H]$^+$ for **exp 1**

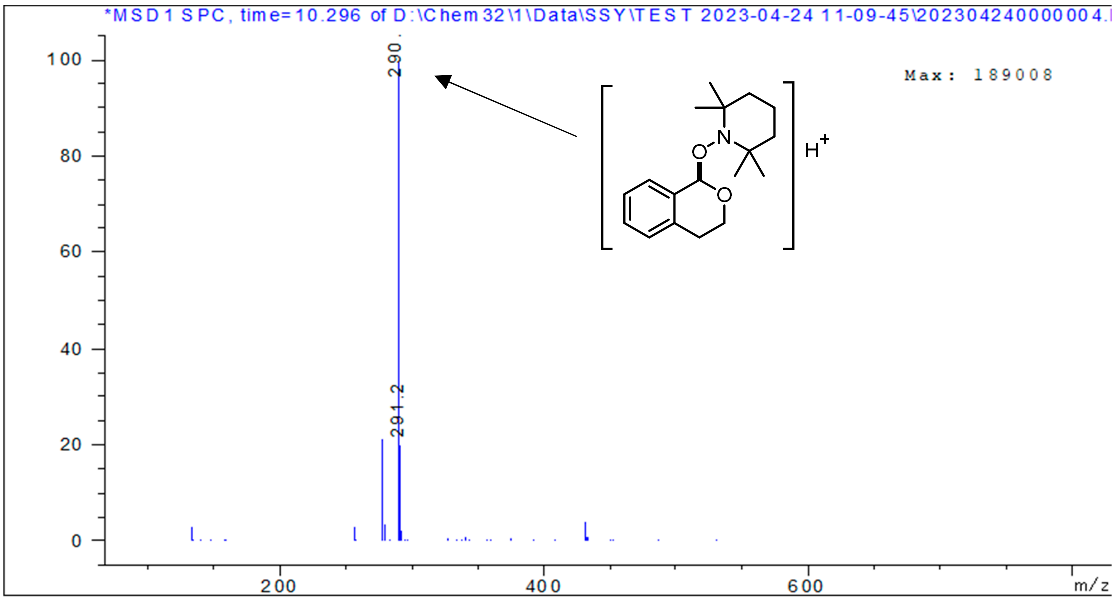

Figure S6. LC-MS spectrum of [**II** + H]$^+$ for **exp 1**

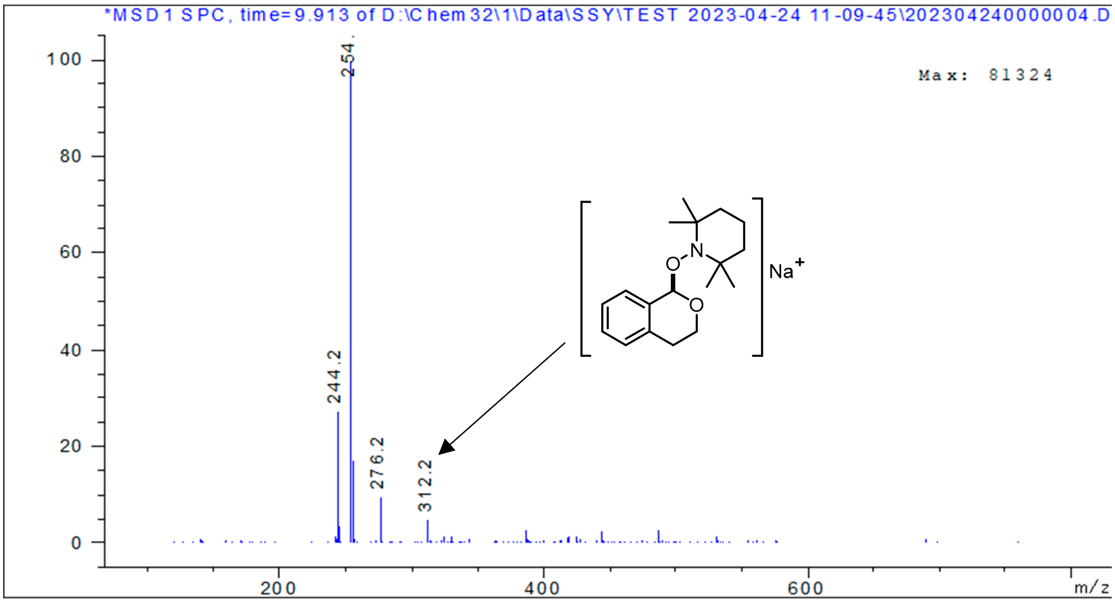

Figure S7. LC-MS spectrum of [**II** + Na]$^+$ for **exp 1**

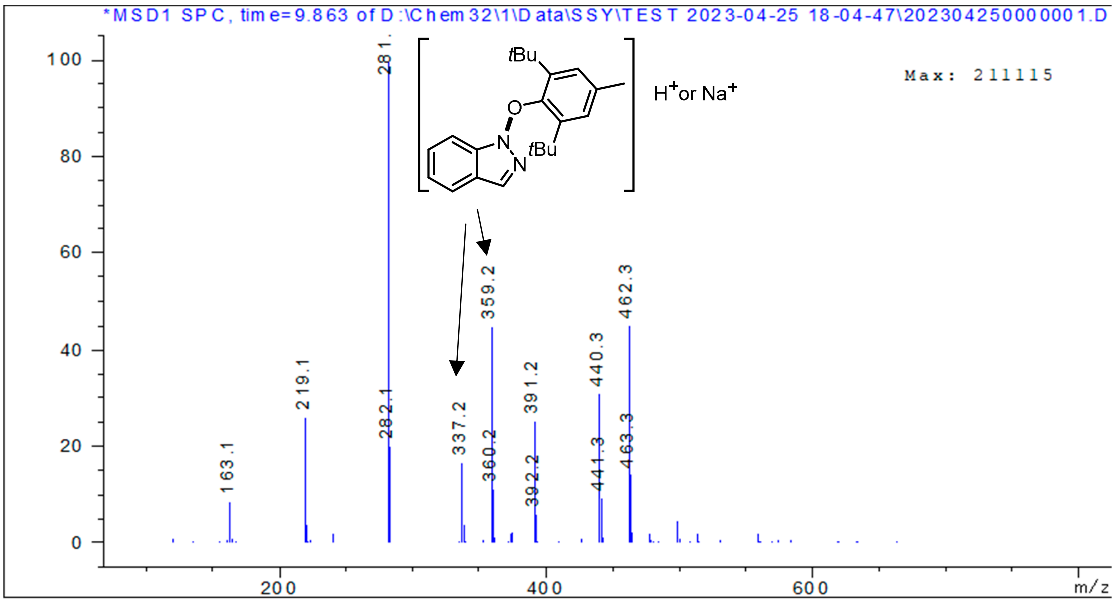

Figure S8. LC-MS spectrum of [**III** + H]$^+$ and [**III** + Na]$^+$ for **exp 2**

5. ^{1}H, ^{13}C and ^{19}F NMR Spectra

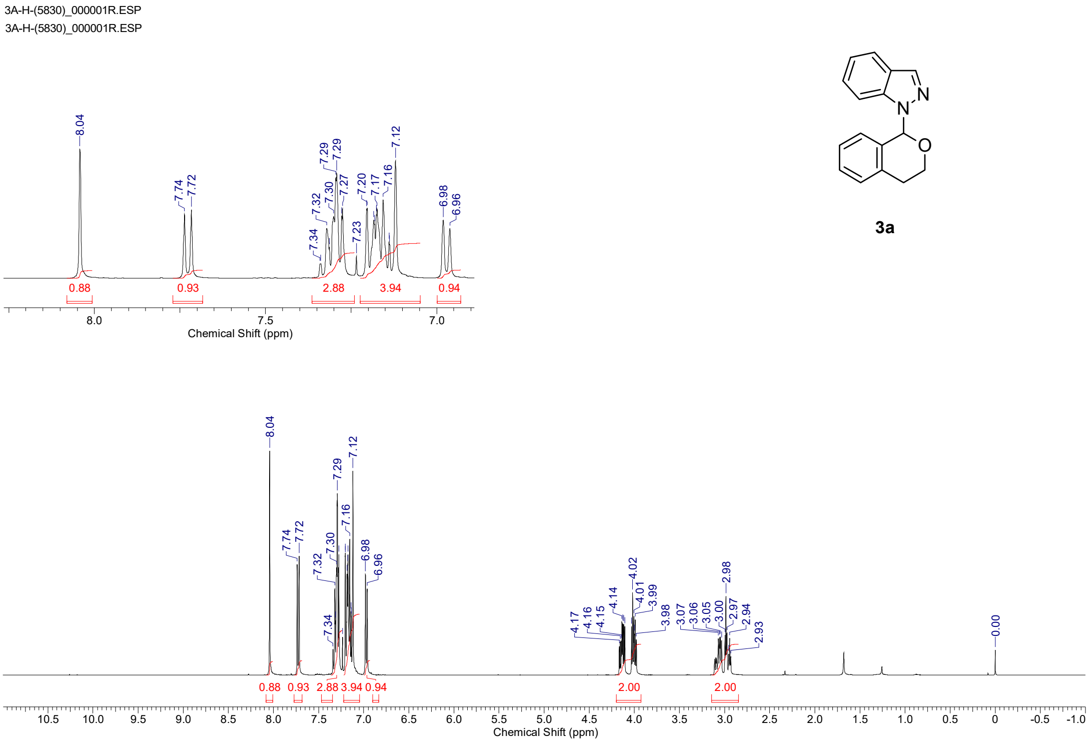

Figure S9. ^{1}H NMR spectra of compound **3a**

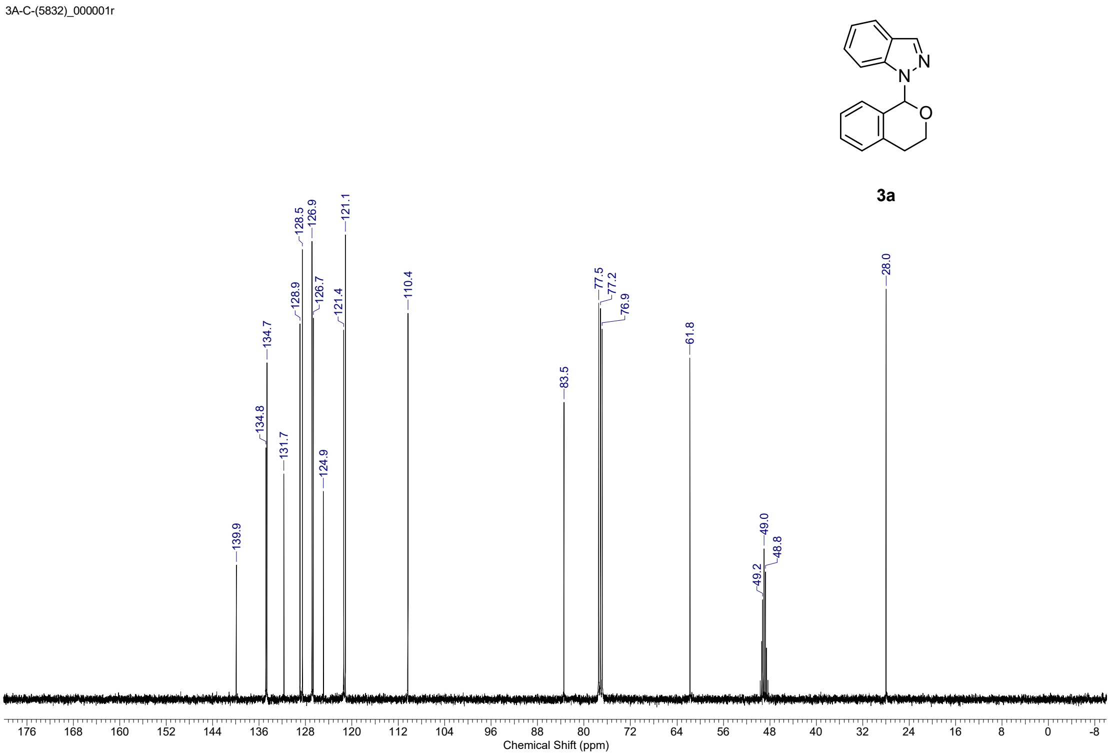

Figure S10. ^{13}C NMR spectra of compound **3a**

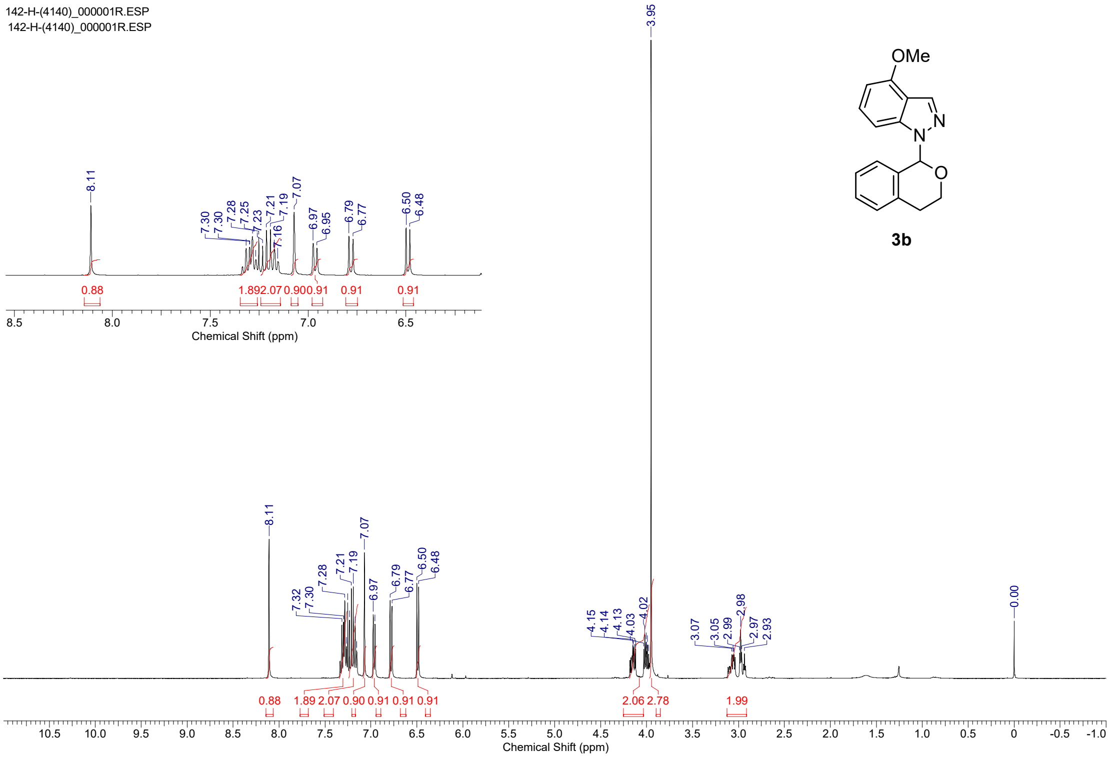

Figure S11. ^{1}H NMR spectra of compound **3b**

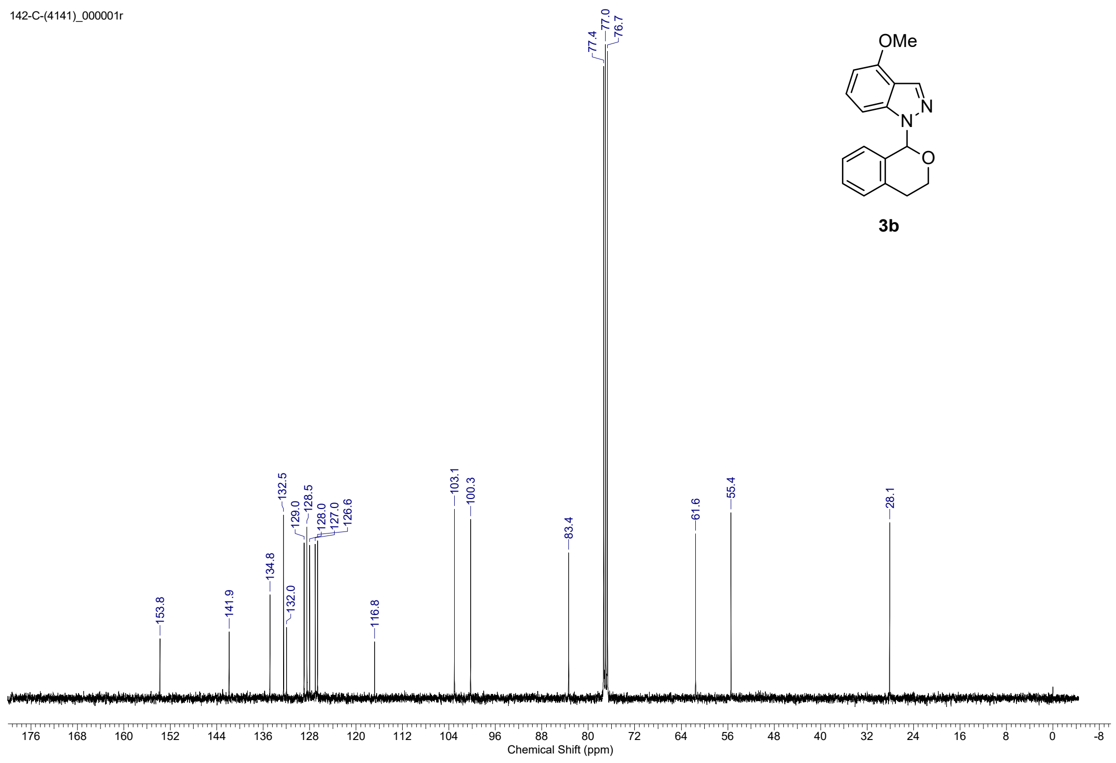

Figure S12. ^{13}C NMR spectra of compound **3b**

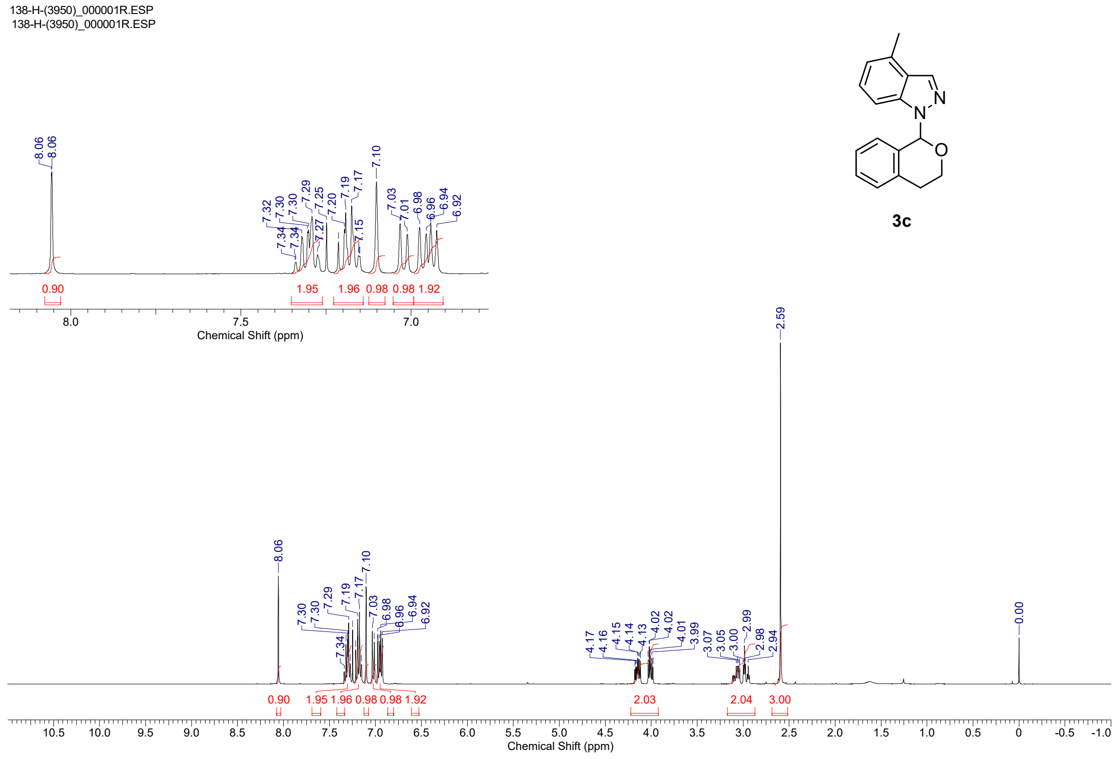

Figure S13. ^{1}H NMR spectra of compound **3c**

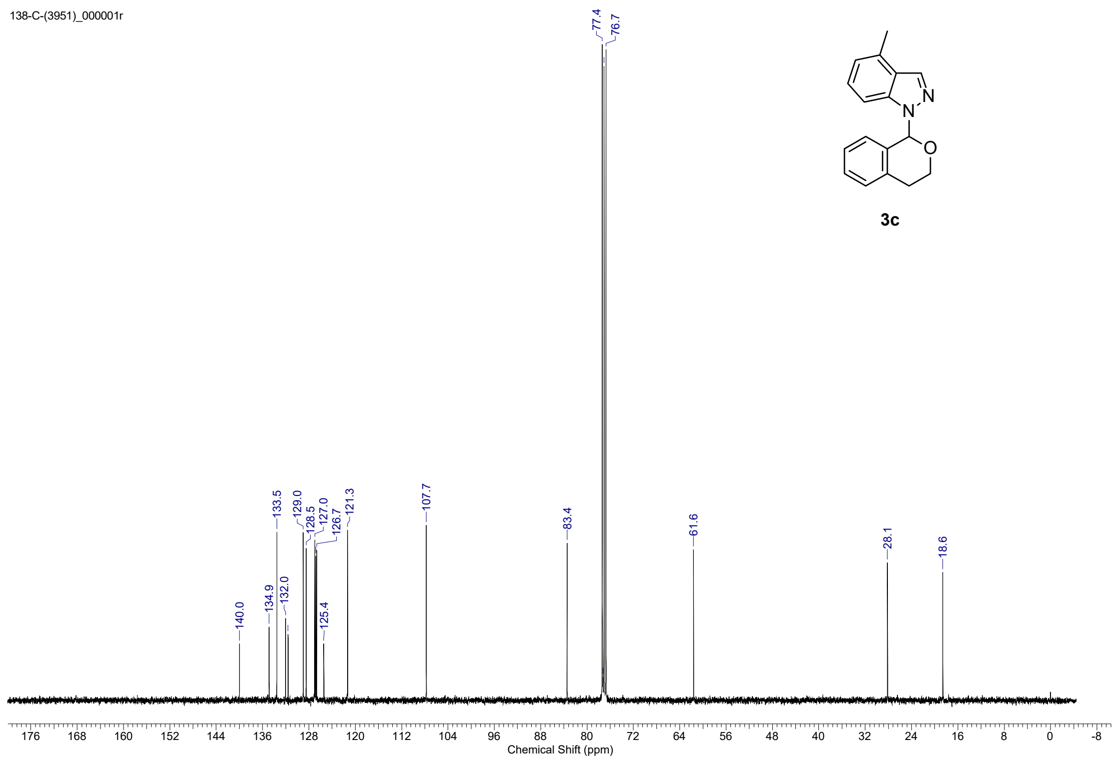

Figure S14. ^{13}C NMR spectra of compound **3c**

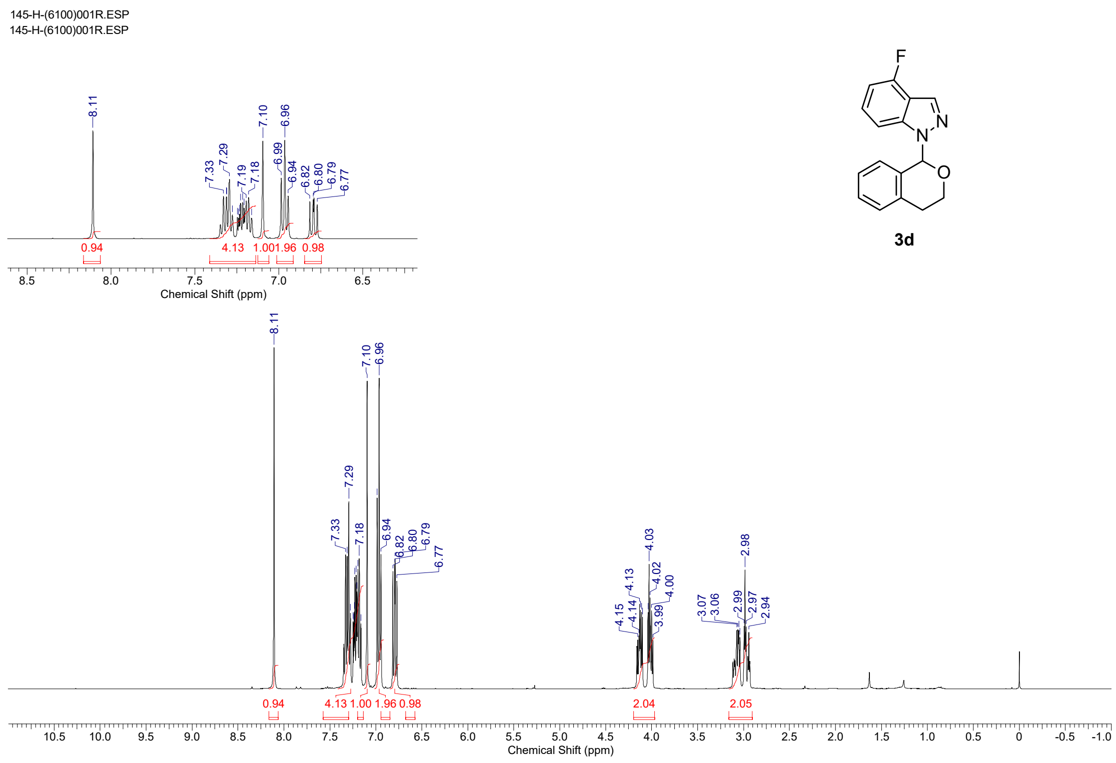

Figure S15. ^{1}H NMR spectra of compound **3d**

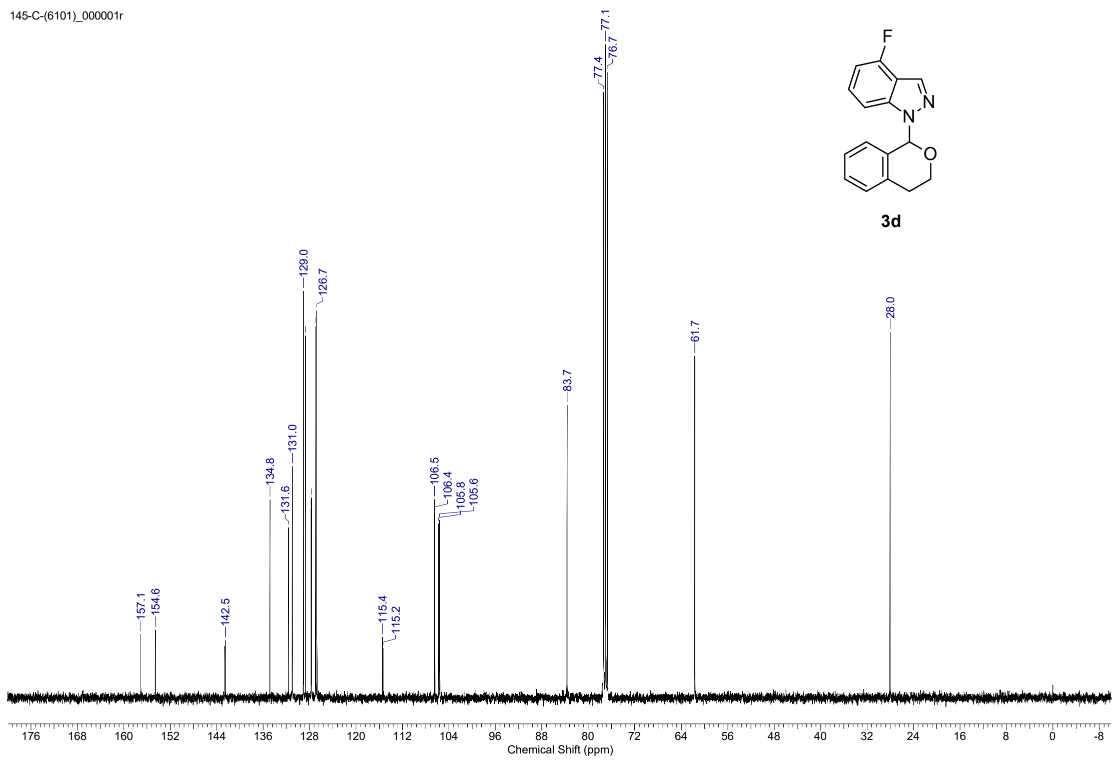

Figure S16. ^{13}C NMR spectra of compound **3d**

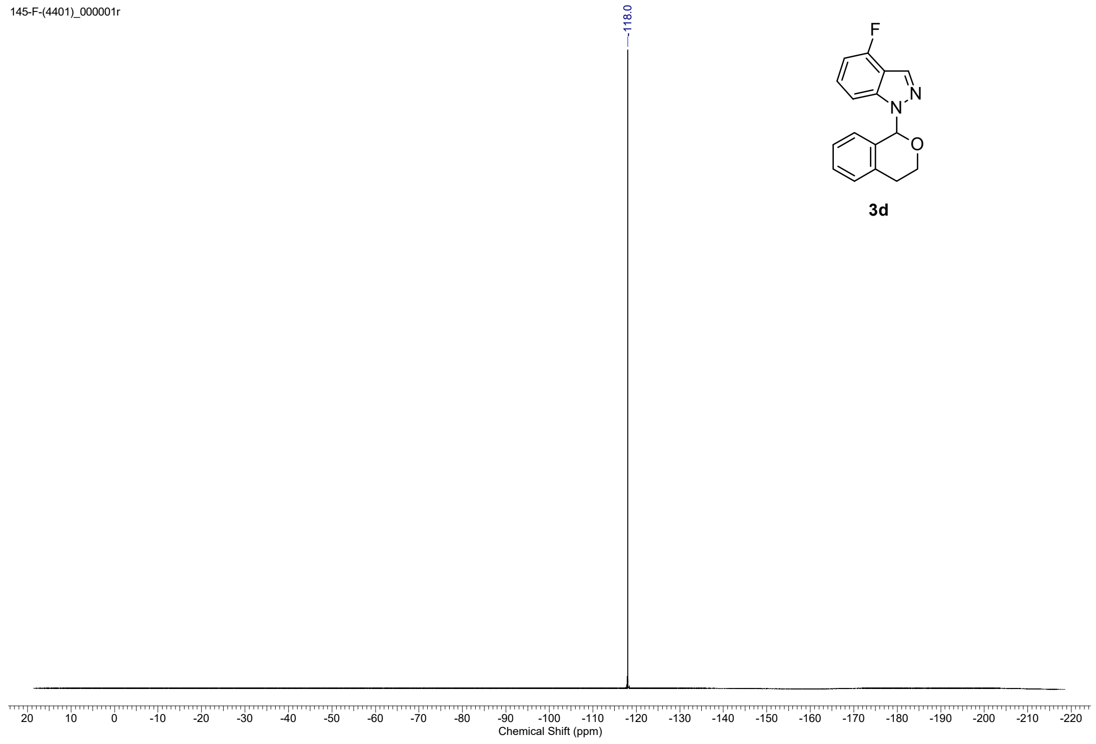

Figure S17. ^{19}F NMR spectra of compound **3d**

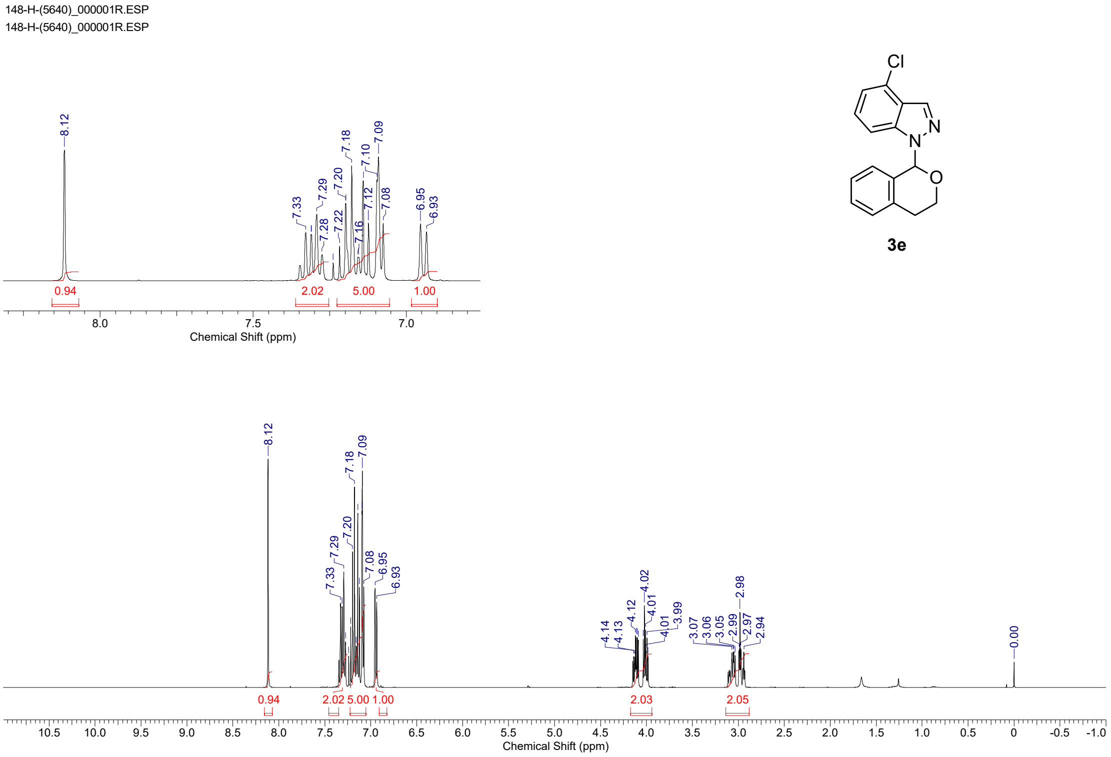

Figure S18. ^{1}H NMR spectra of compound **3e**

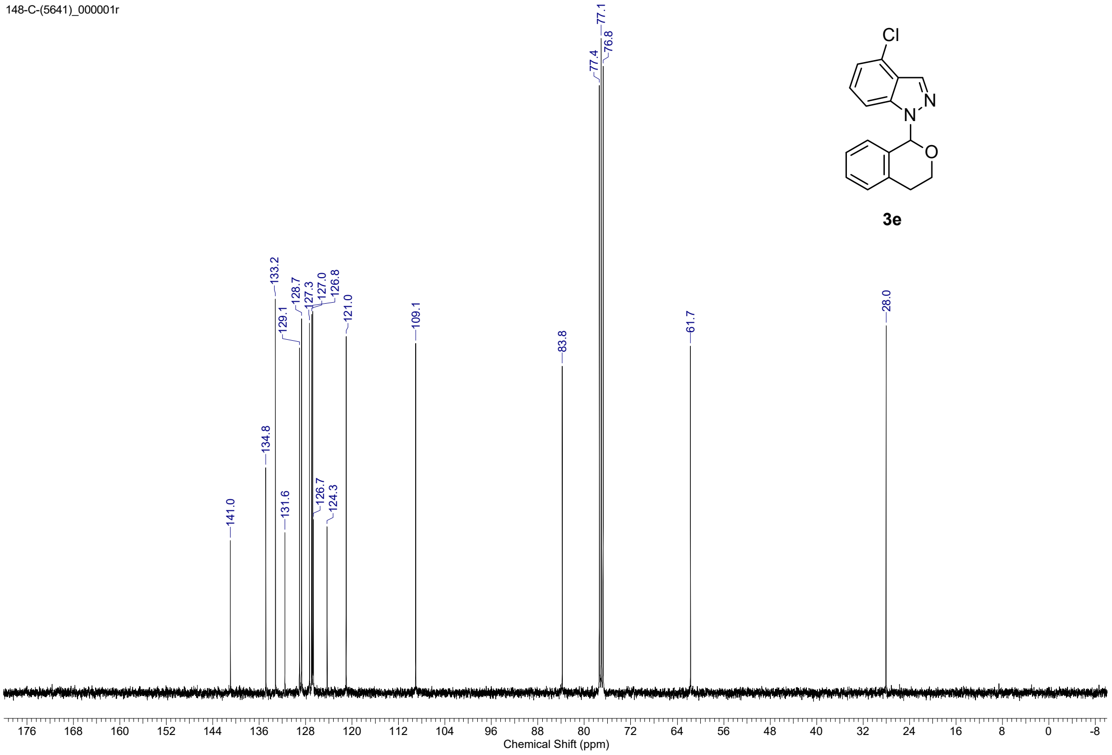

Figure S19. ^{13}C NMR spectra of compound **3e**

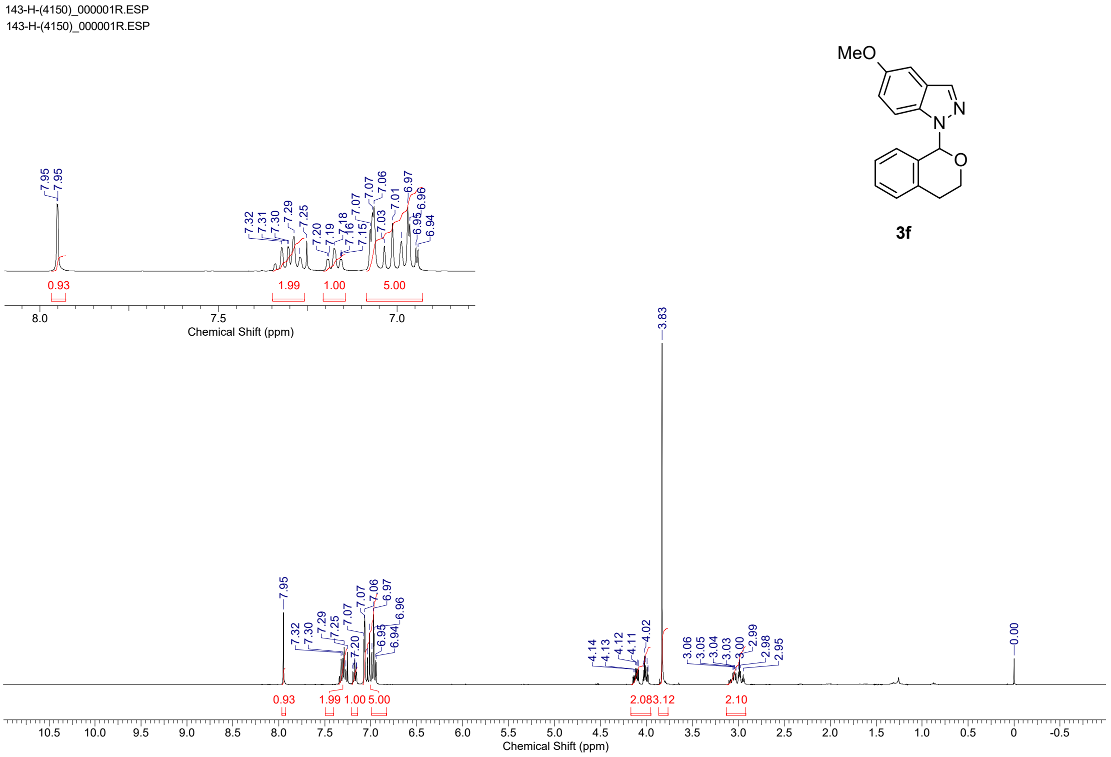

Figure S20. ^{1}H NMR spectra of compound **3f**

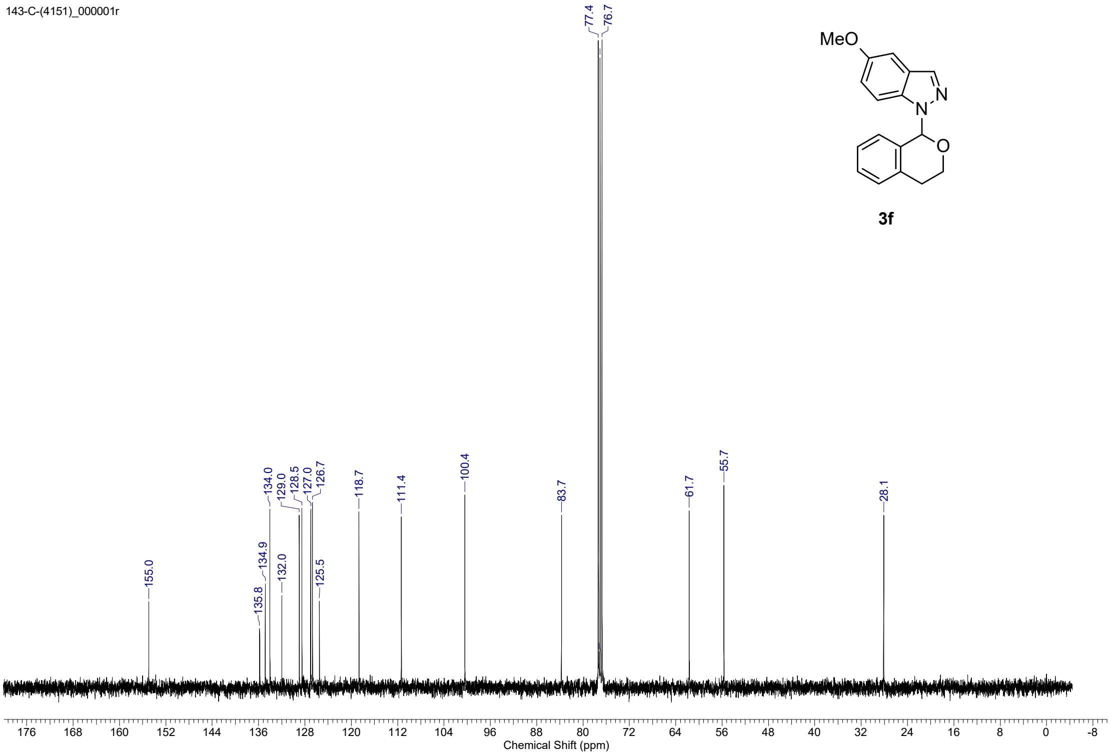

Figure S21. ^{13}C NMR spectra of compound **3f**

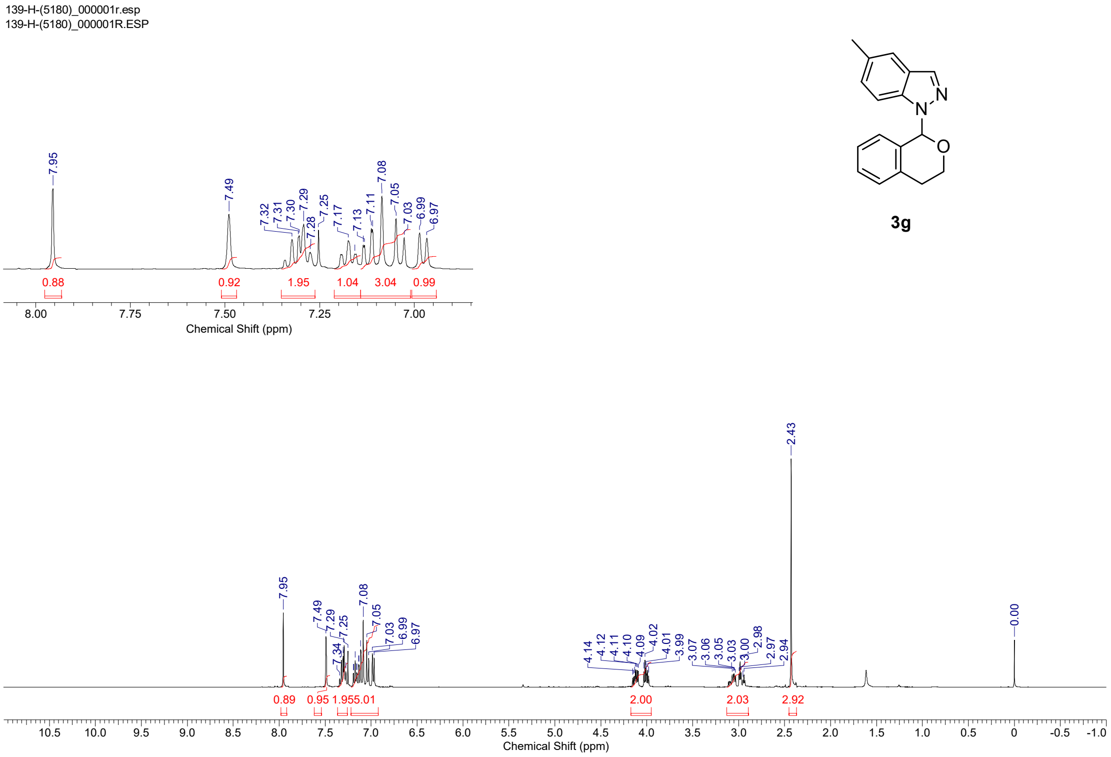

Figure S22. ^{1}H NMR spectra of compound **3g**

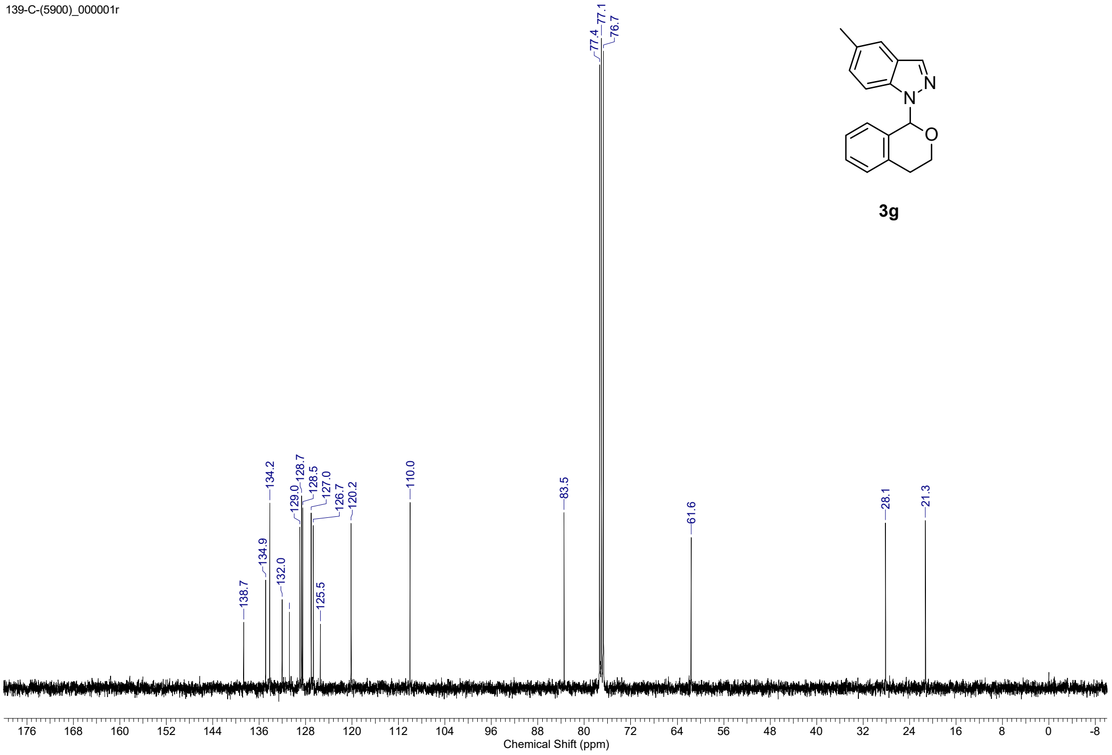

Figure S23. ^{13}C NMR spectra of compound **3g**

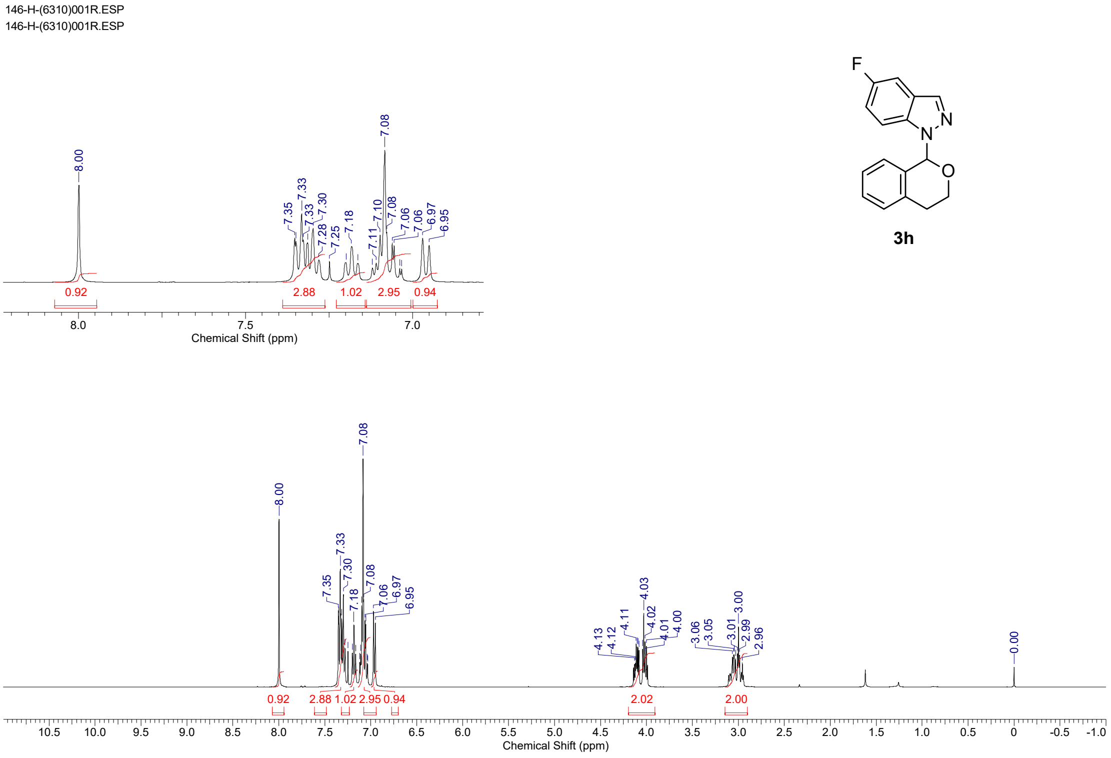

Figure S24. ^{1}H NMR spectra of compound **3h**

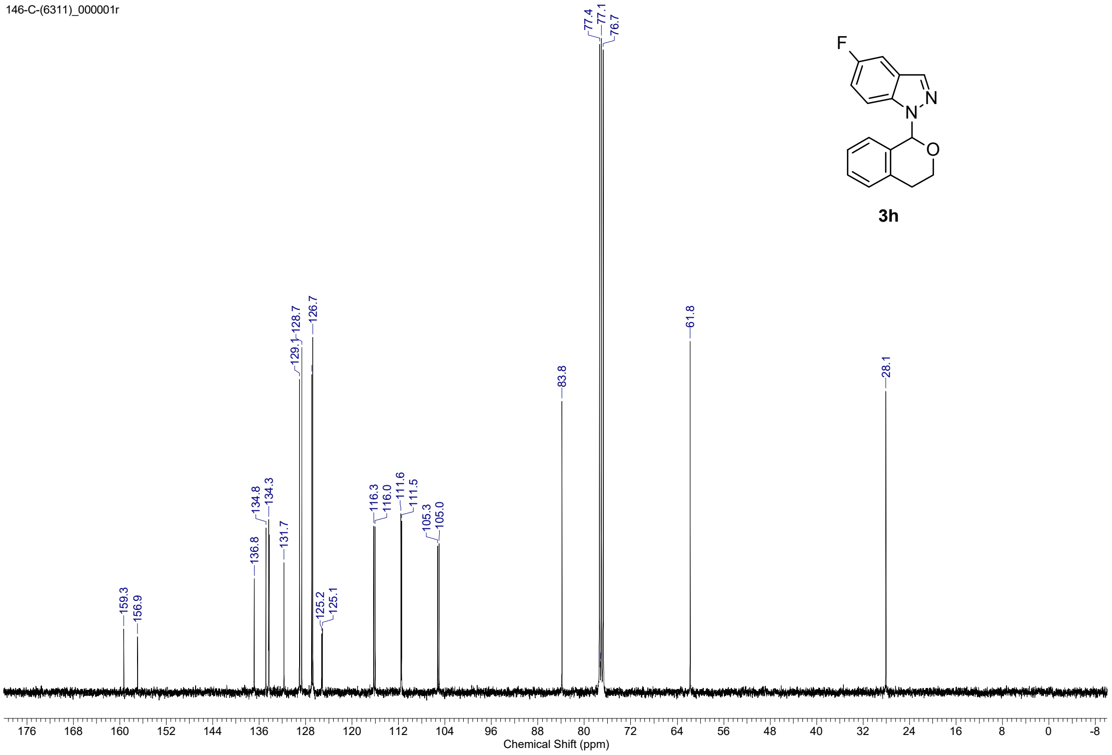

Figure S25. ^{13}C NMR spectra of compound **3h**

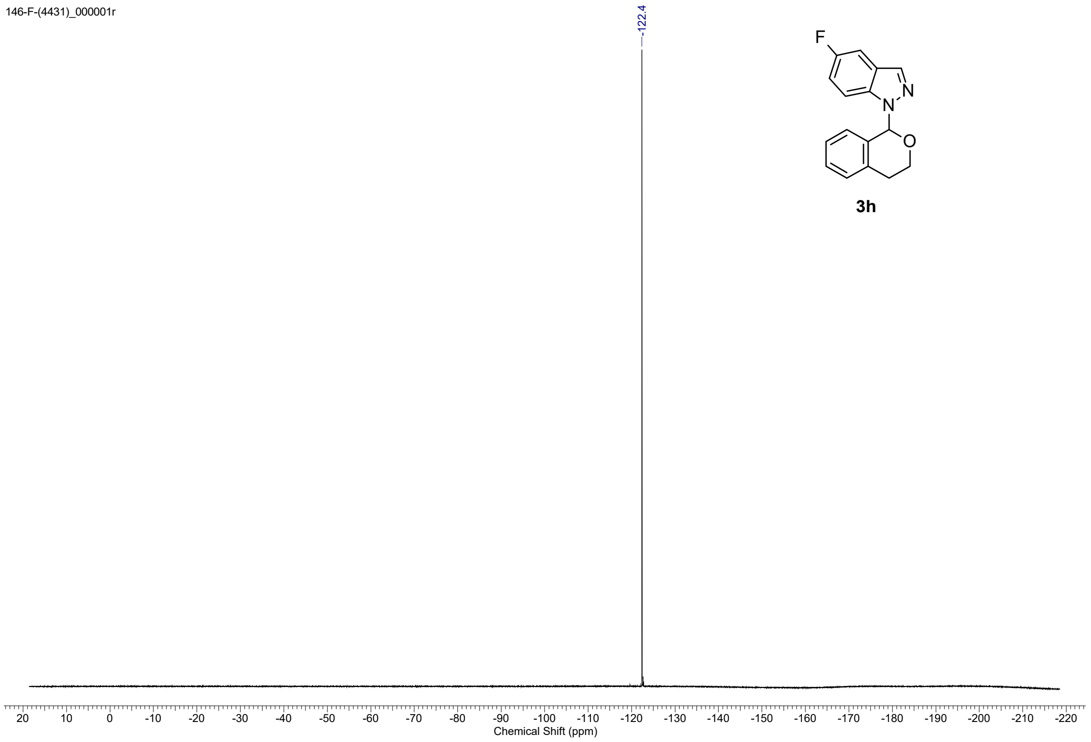

Figure S26. ^{19}F NMR spectra of compound **3h**

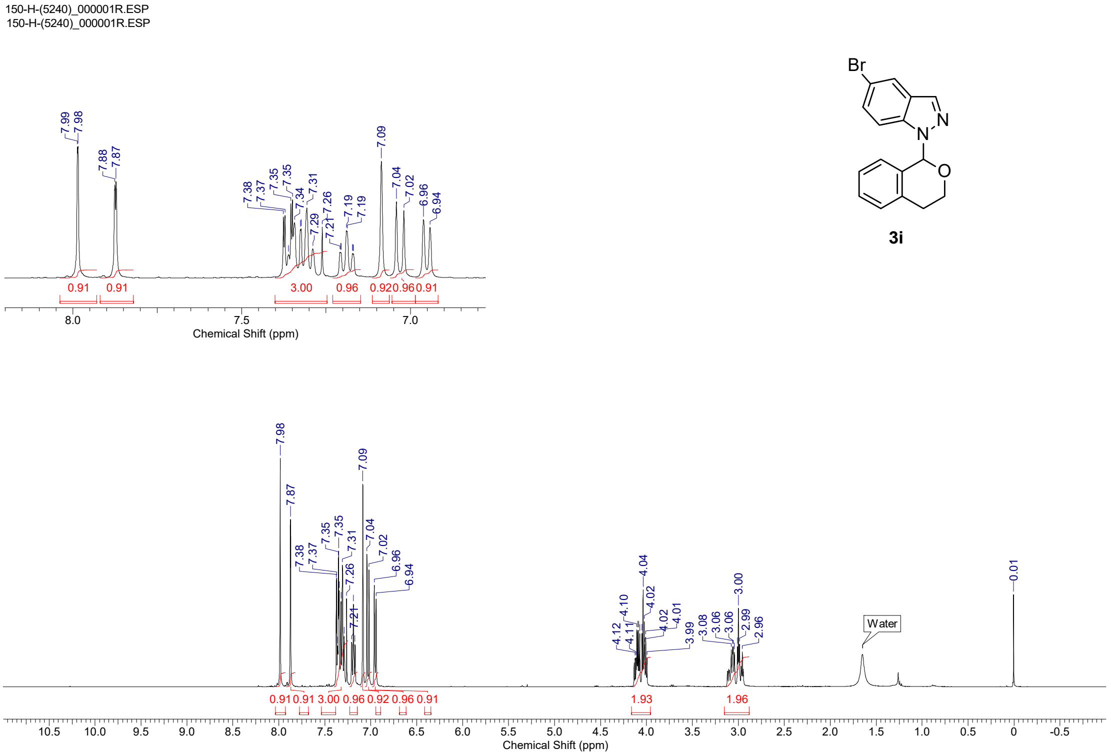

Figure S27. ^{1}H NMR spectra of compound **3i**

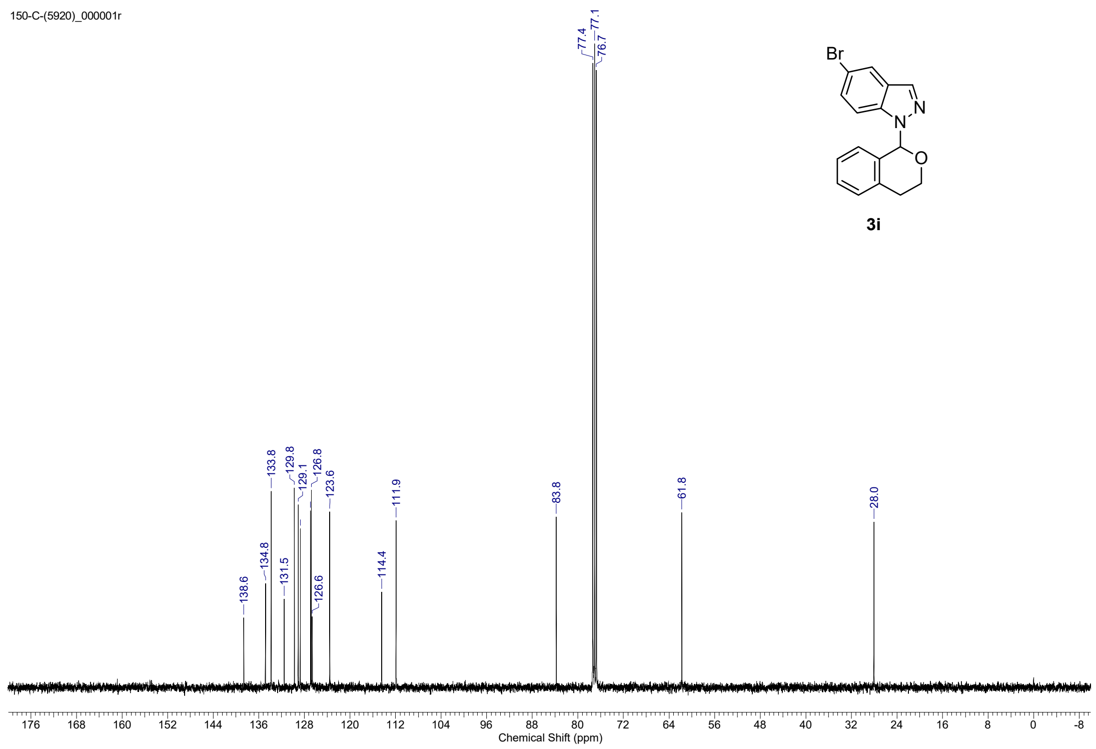

Figure S28. ^{13}C NMR spectra of compound **3i**

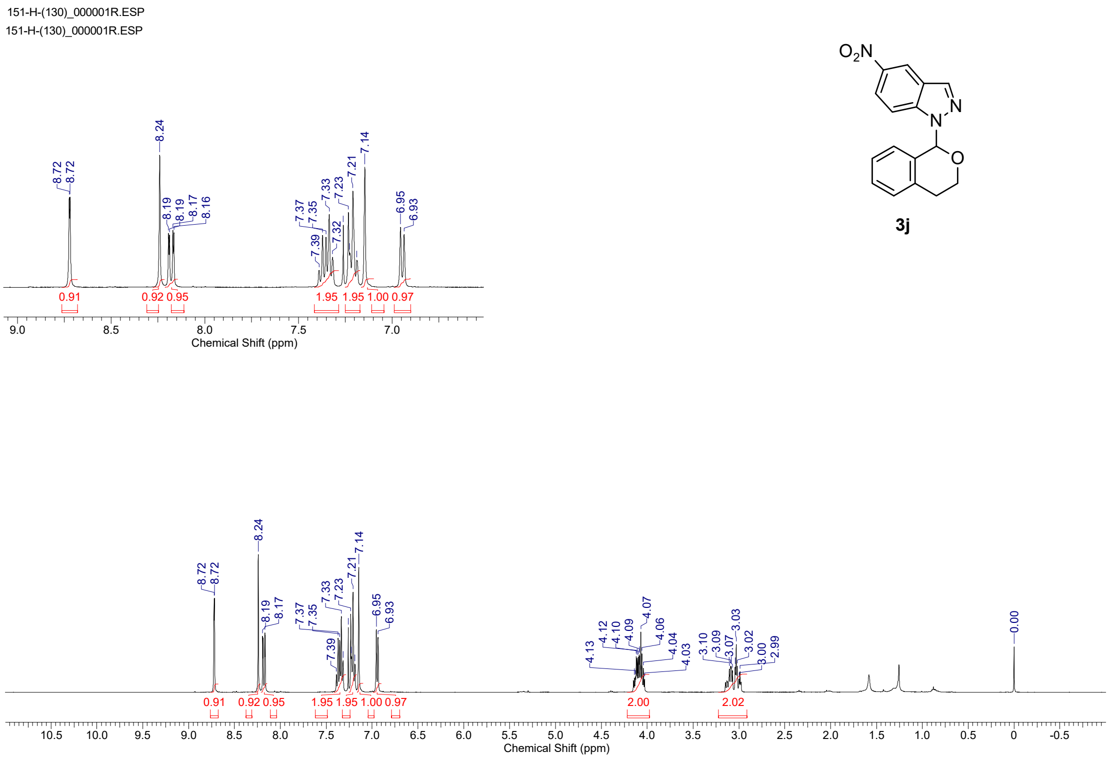

Figure S29. ^{1}H NMR spectra of compound **3j**

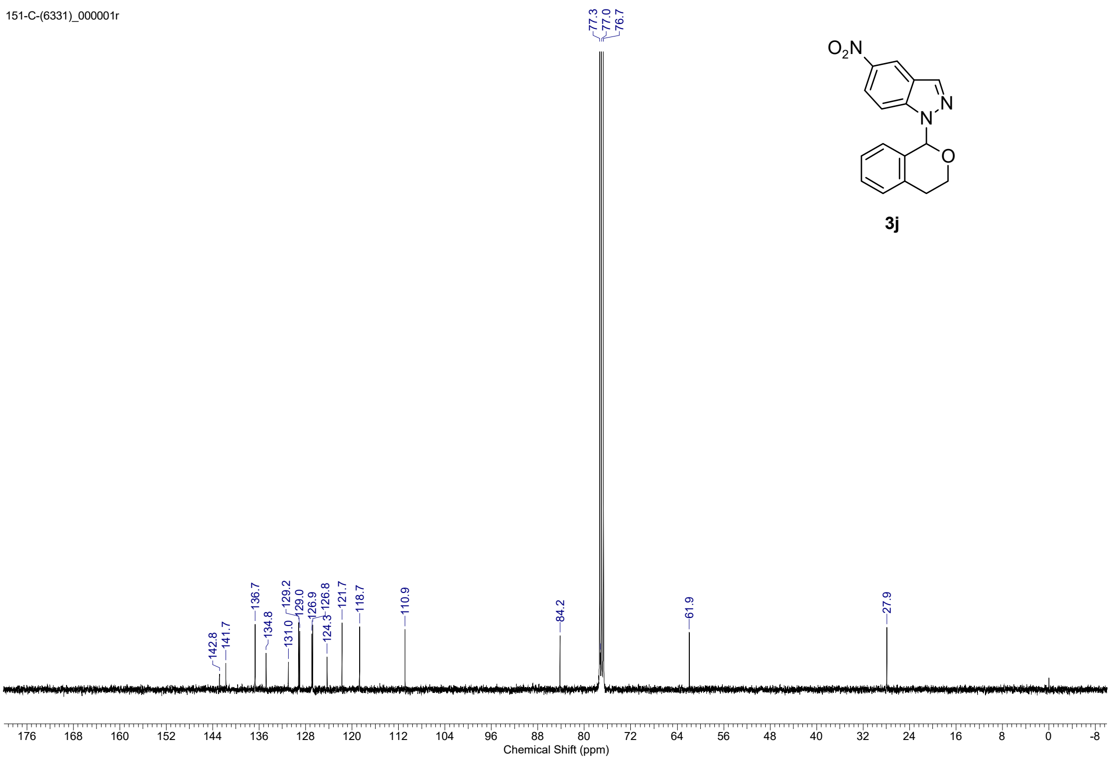

Figure S30. ^{13}C NMR spectra of compound **3j**

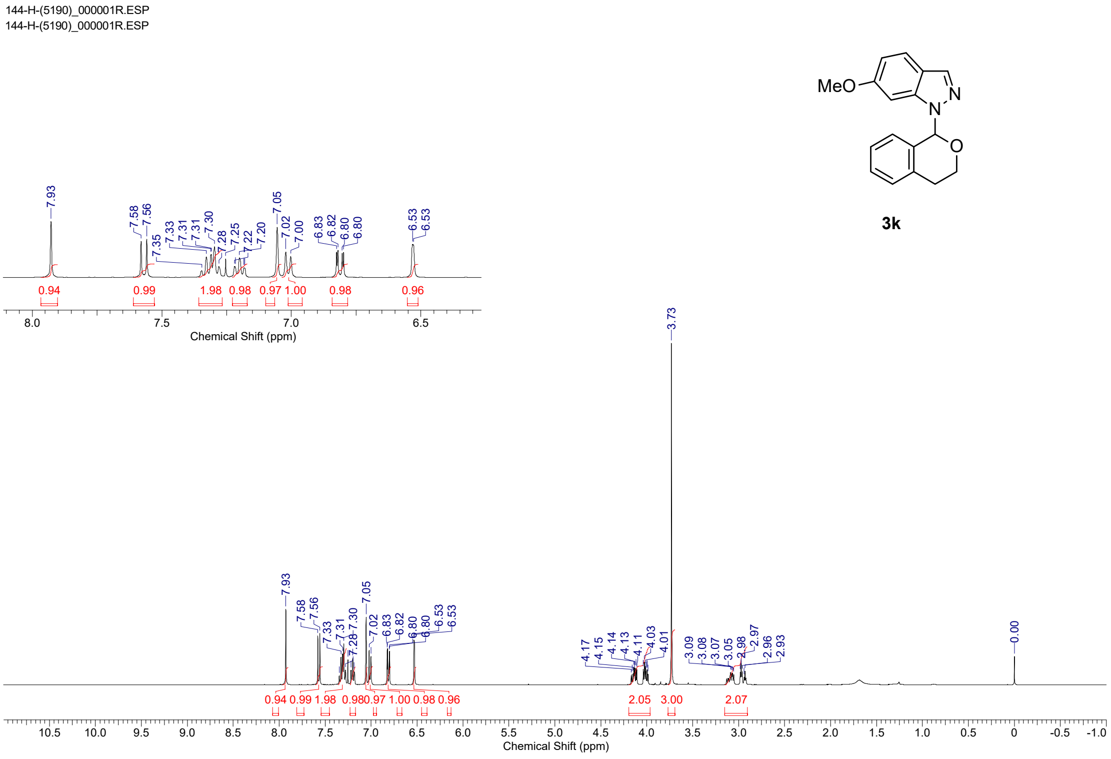

Figure S31. ^{1}H NMR spectra of compound **3k**

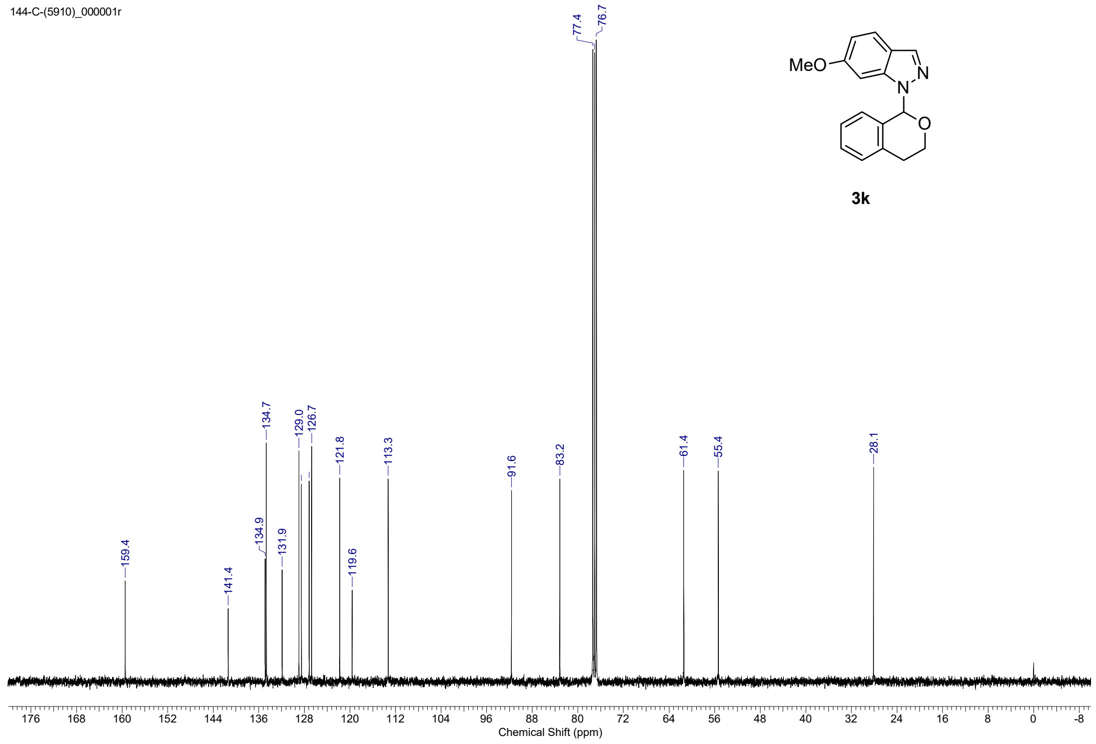

Figure S32. ^{13}C NMR spectra of compound **3k**

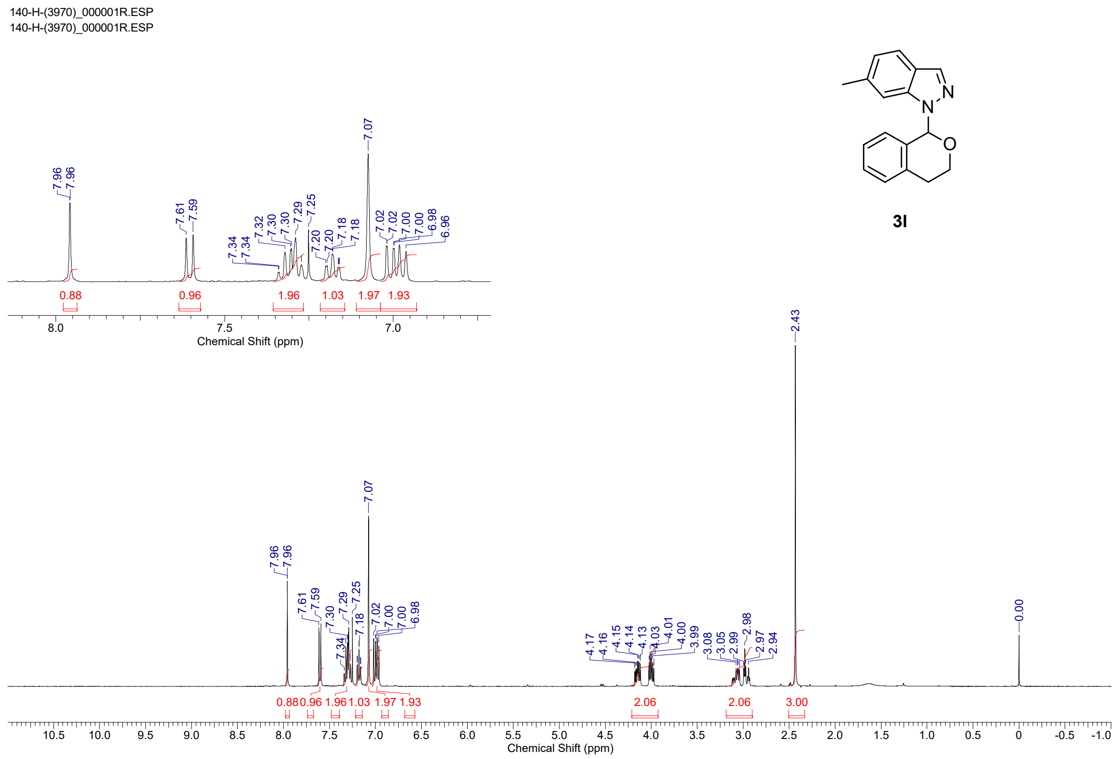

Figure S33. ^{1}H NMR spectra of compound **3l**

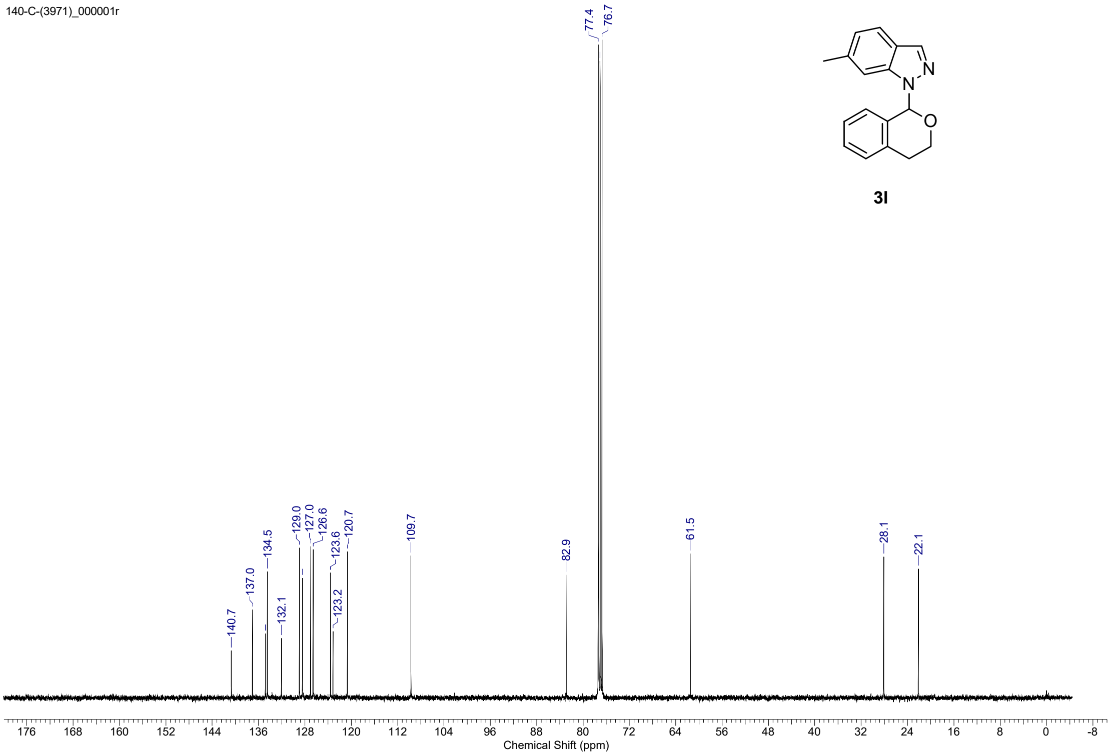

Figure S34. ^{13}C NMR spectra of compound **3l**

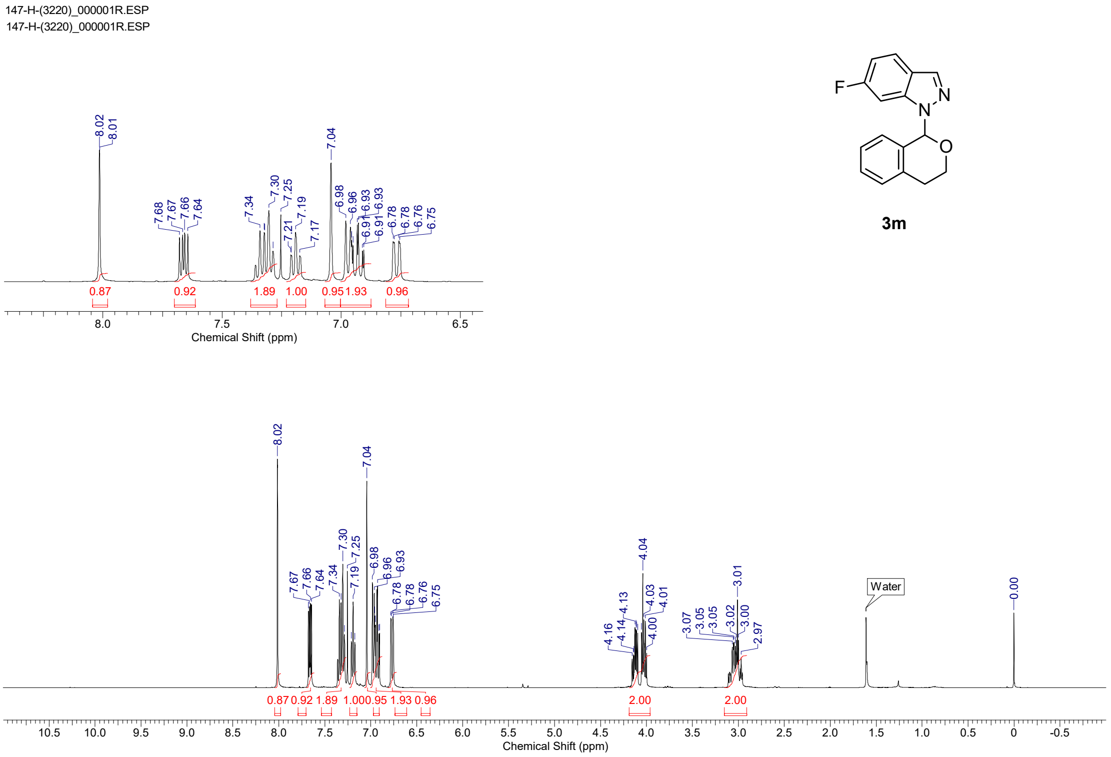

Figure S35. ^{1}H NMR spectra of compound **3m**

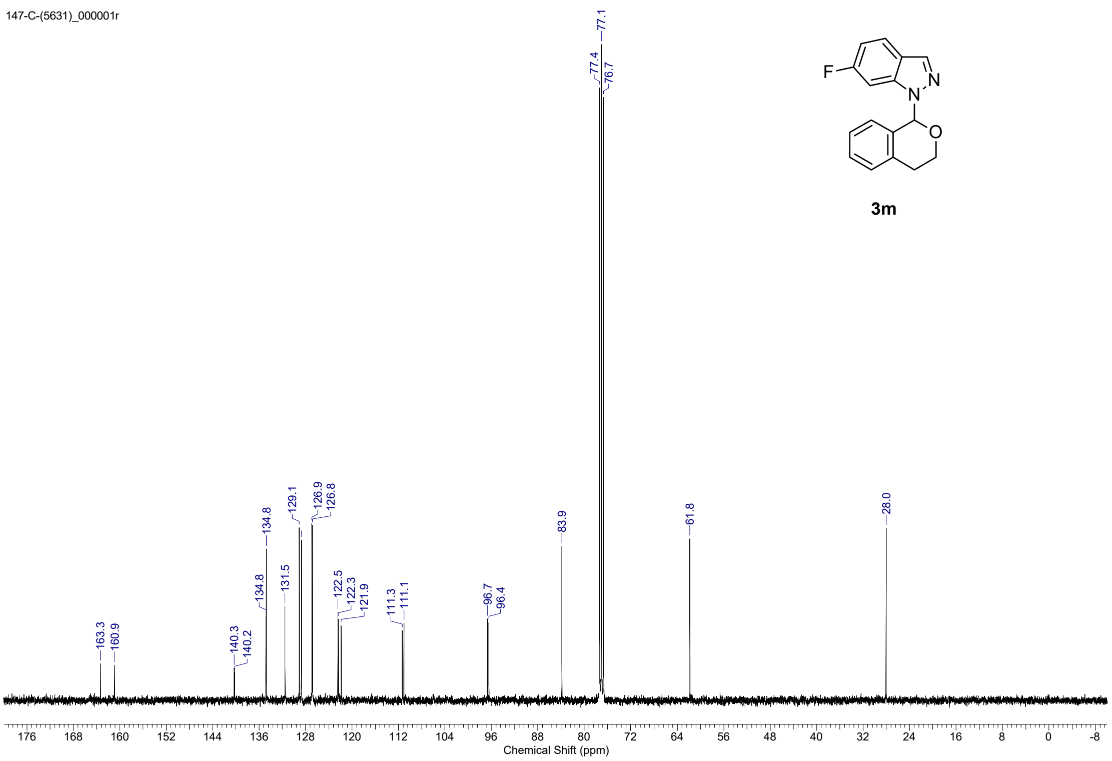

Figure S36. ^{13}C NMR spectra of compound **3m**

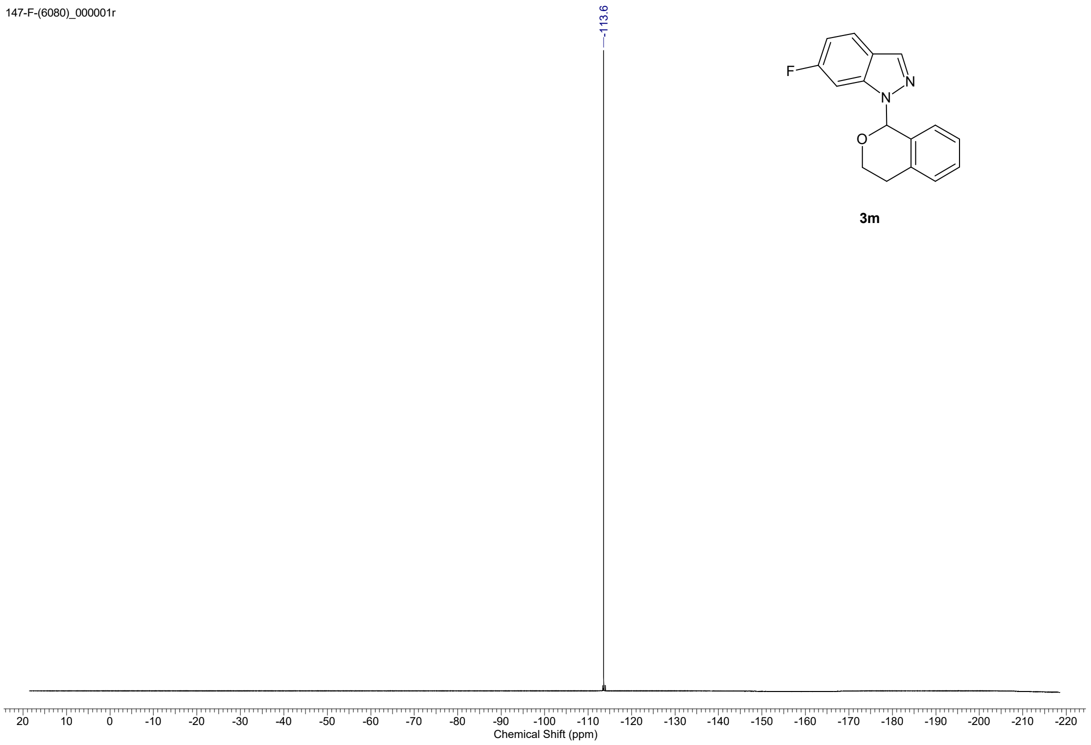

Figure S37. ^{19}F NMR spectra of compound **3m**

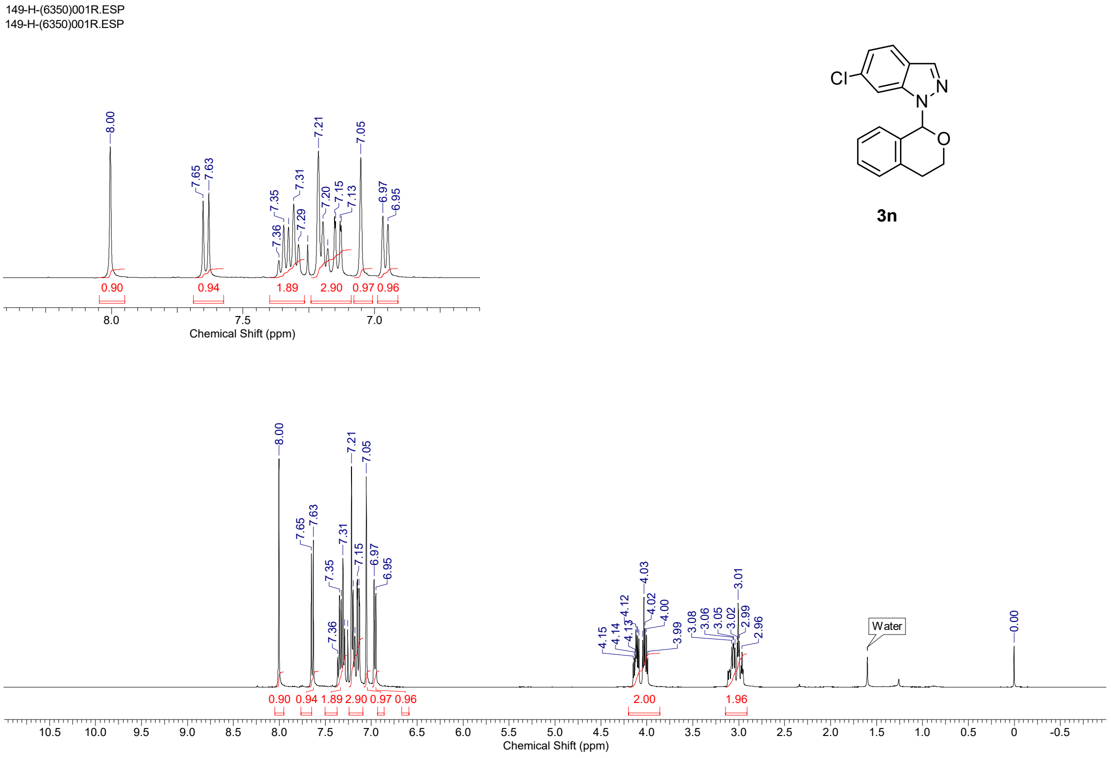

Figure S38. ^{1}H NMR spectra of compound **3n**

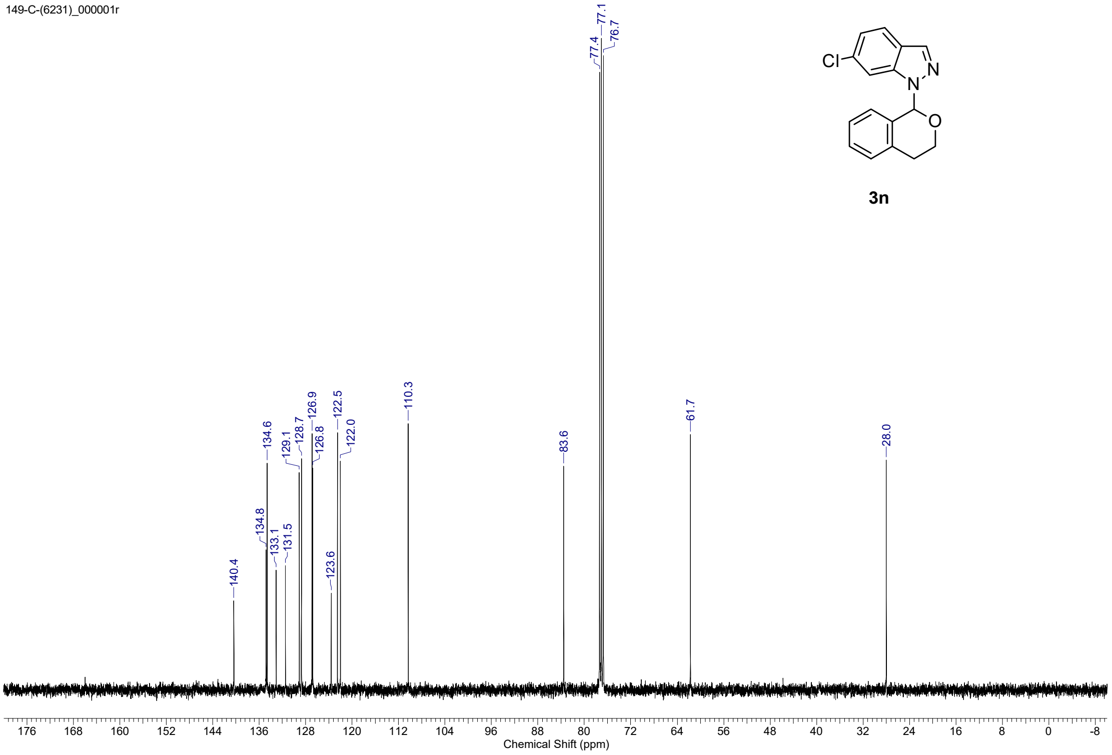

Figure S39. ^{13}C NMR spectra of compound **3n**

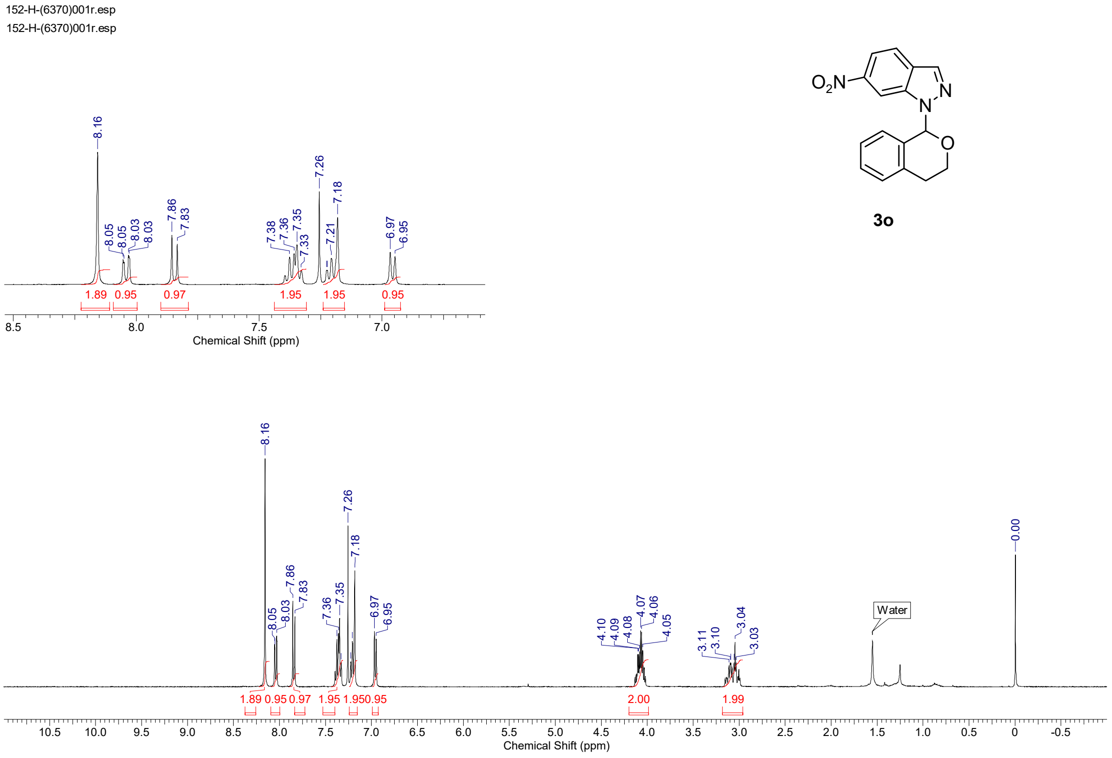

Figure S40. ^{1}H NMR spectra of compound **3o**

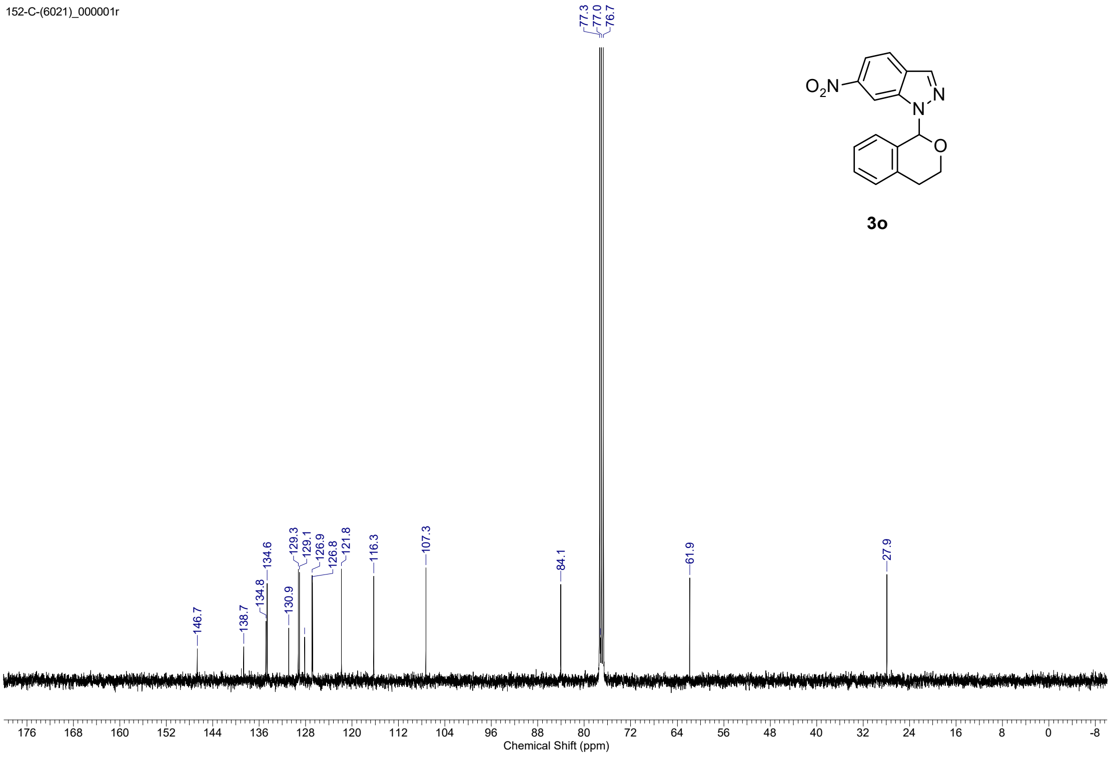

Figure S41. ^{13}C NMR spectra of compound **3o**

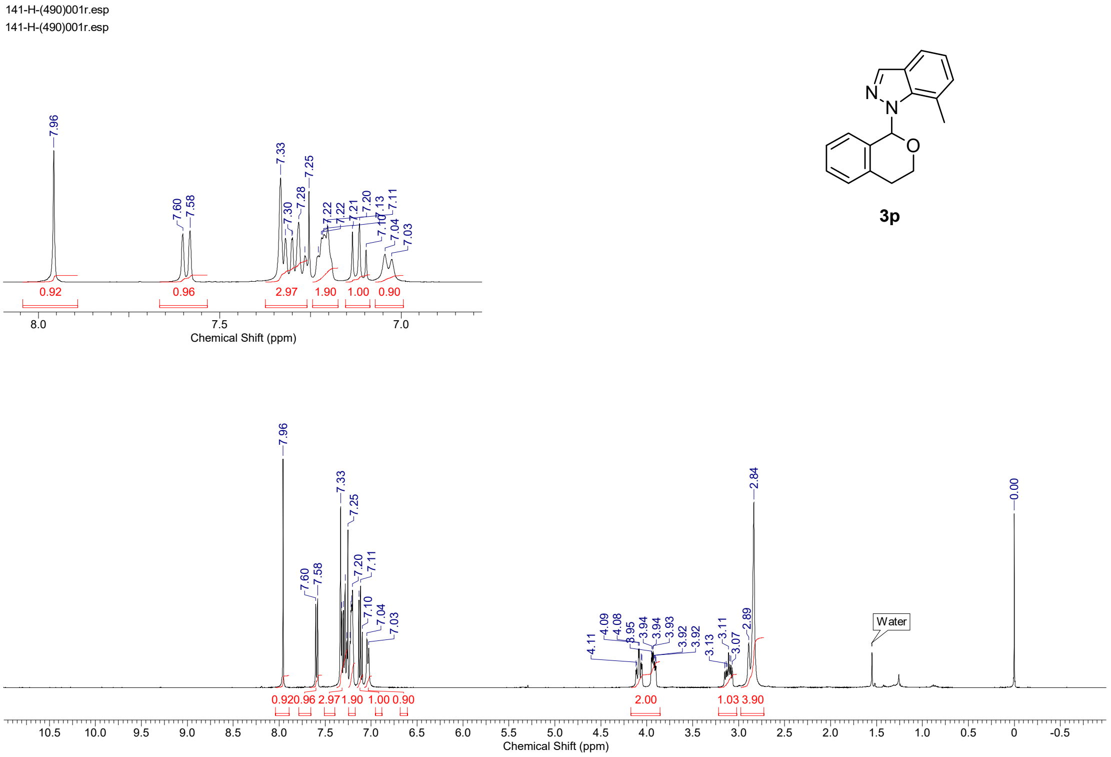

Figure S42. ^{1}H NMR spectra of compound **3p**

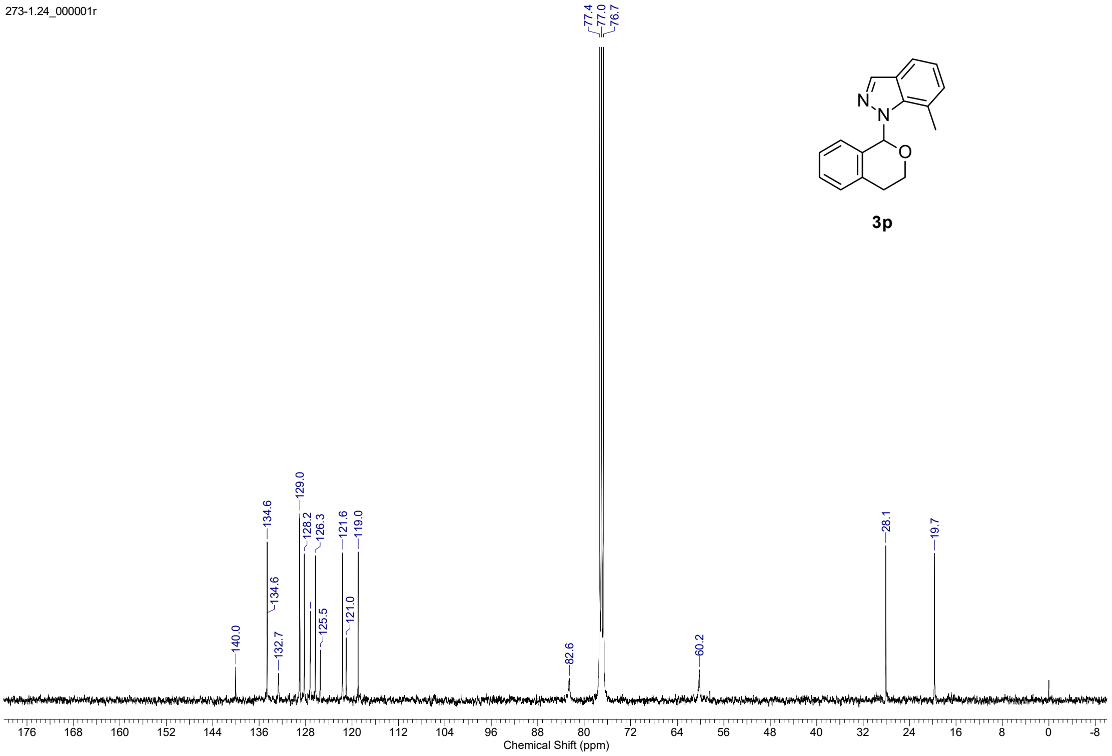

Figure S43. ^{13}C NMR spectra of compound **3p**

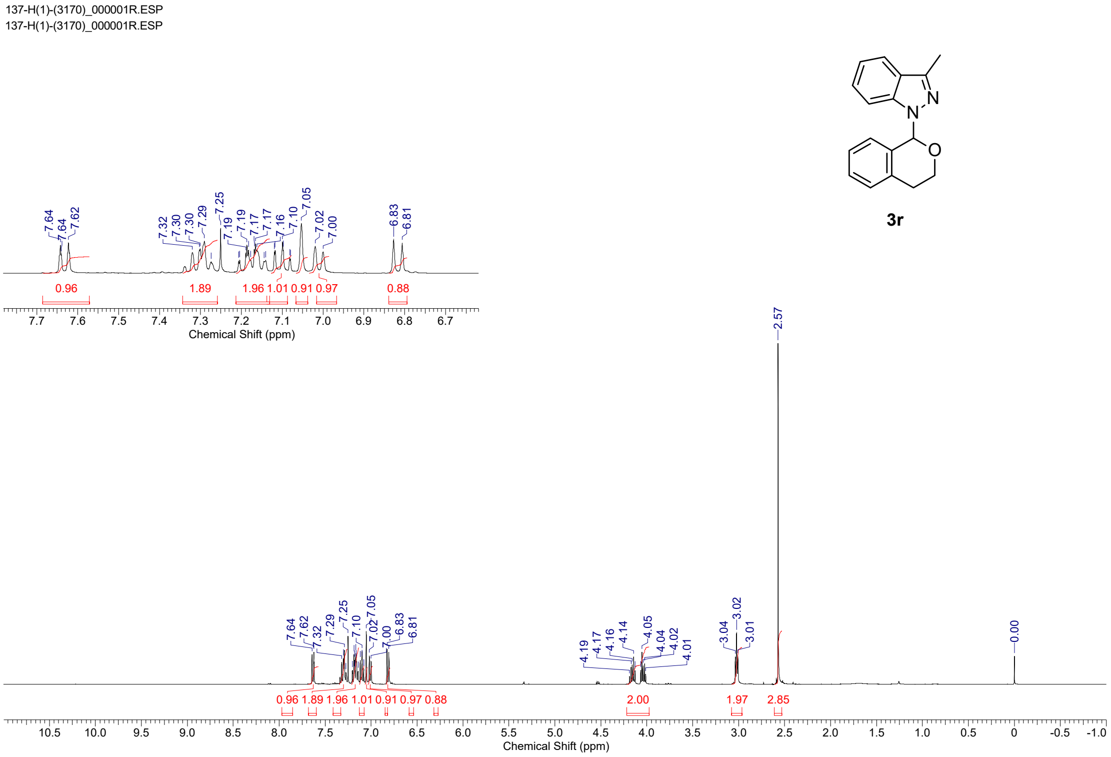

Figure S44. ^{1}H NMR spectra of compound **3r**

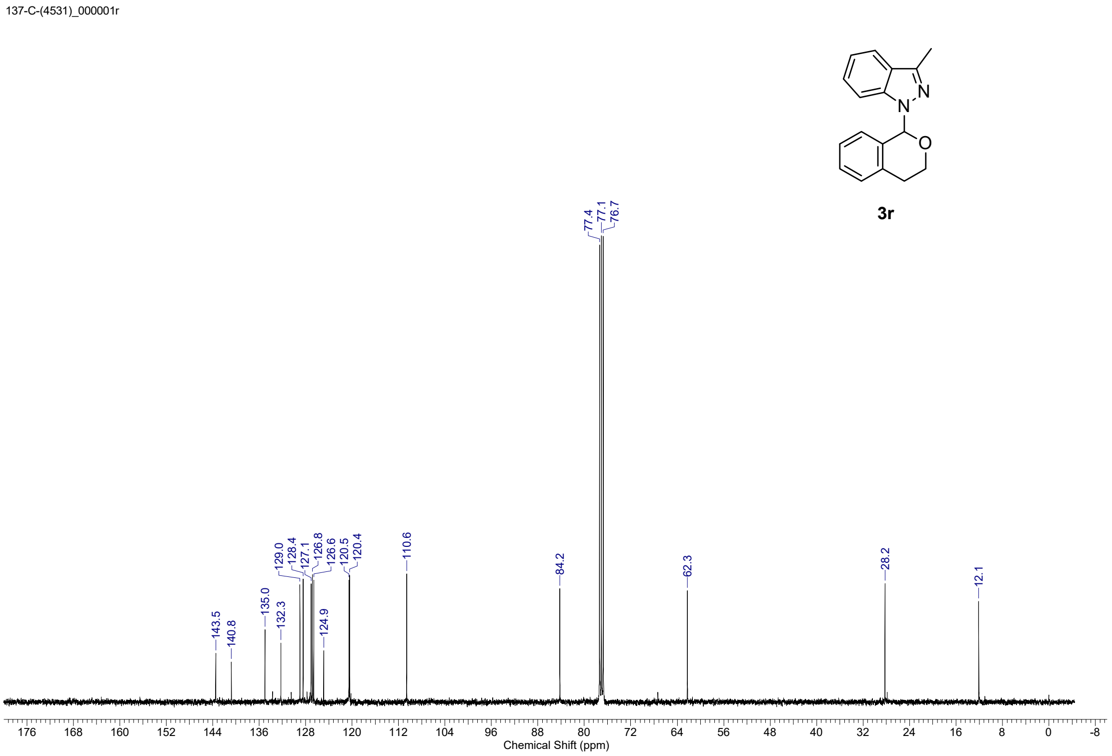

Figure S45. ^{13}C NMR spectra of compound **3r**

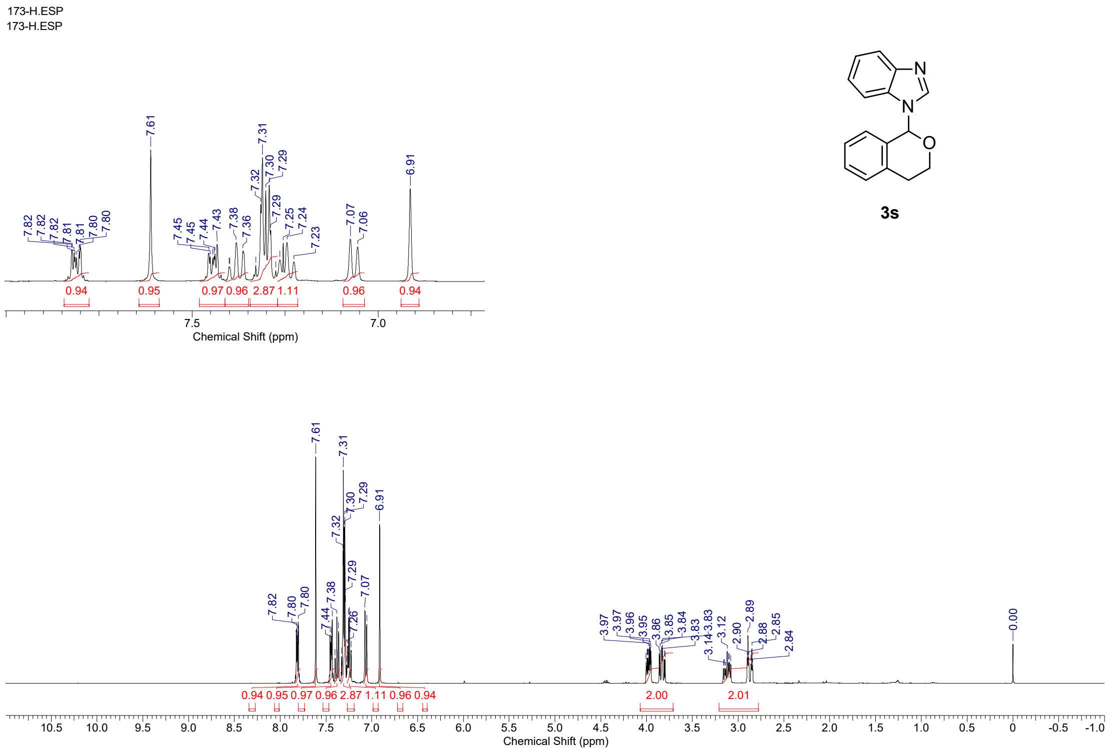

Figure S46. ^{1}H NMR spectra of compound **3s**

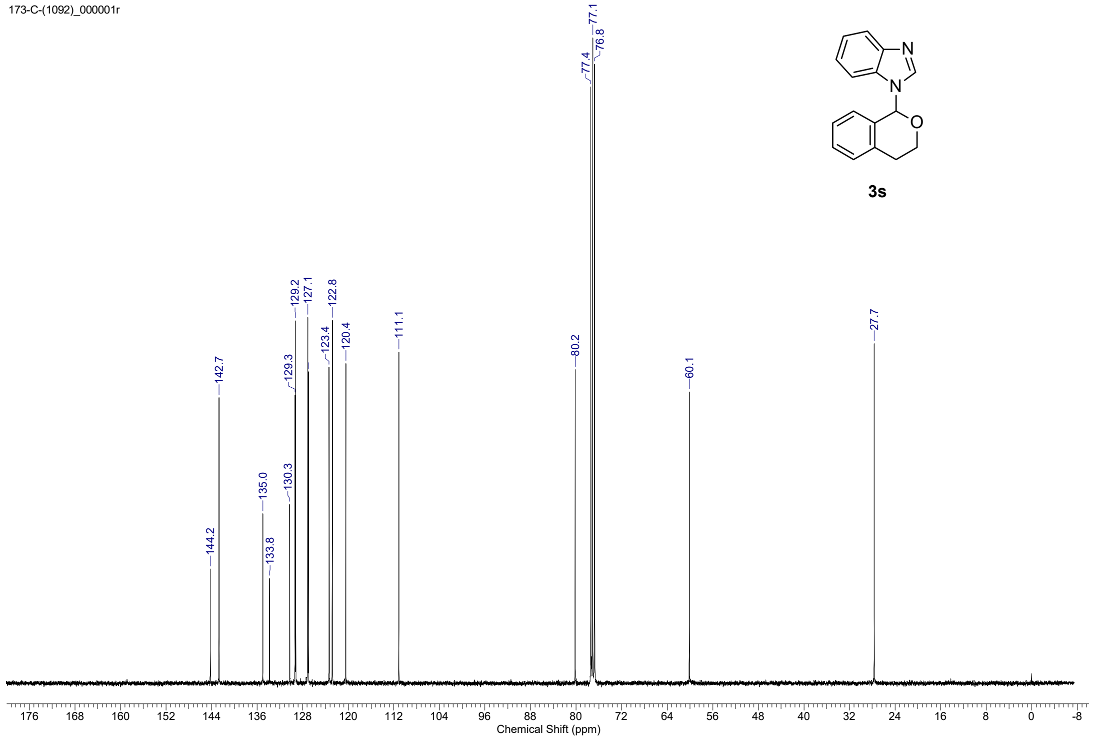

Figure S47. ^{13}C NMR spectra of compound **3s**

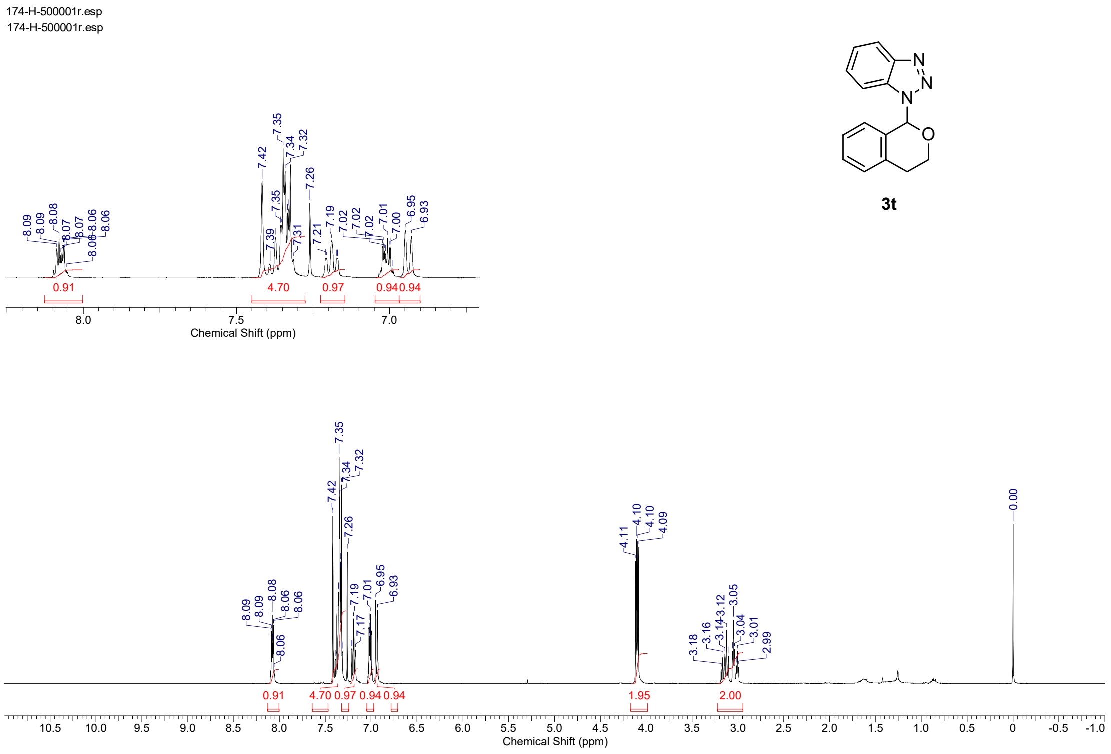

Figure S48. ^{1}H NMR spectra of compound **3t**

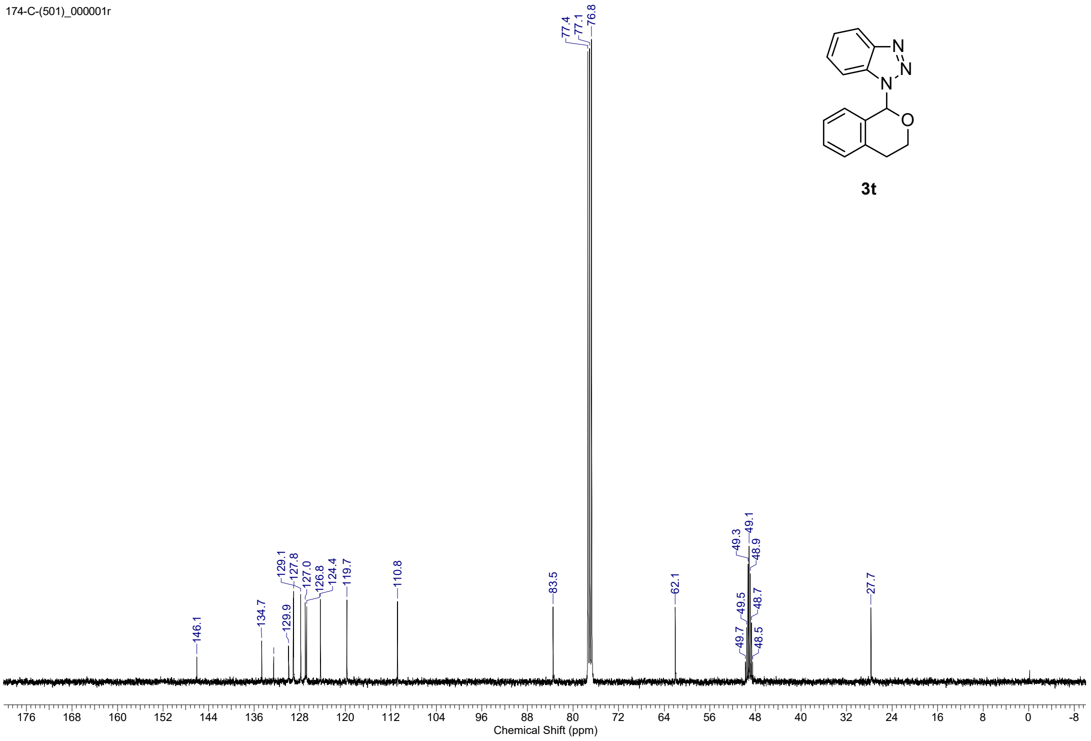

Figure S49. ^{13}C NMR spectra of compound **3t**

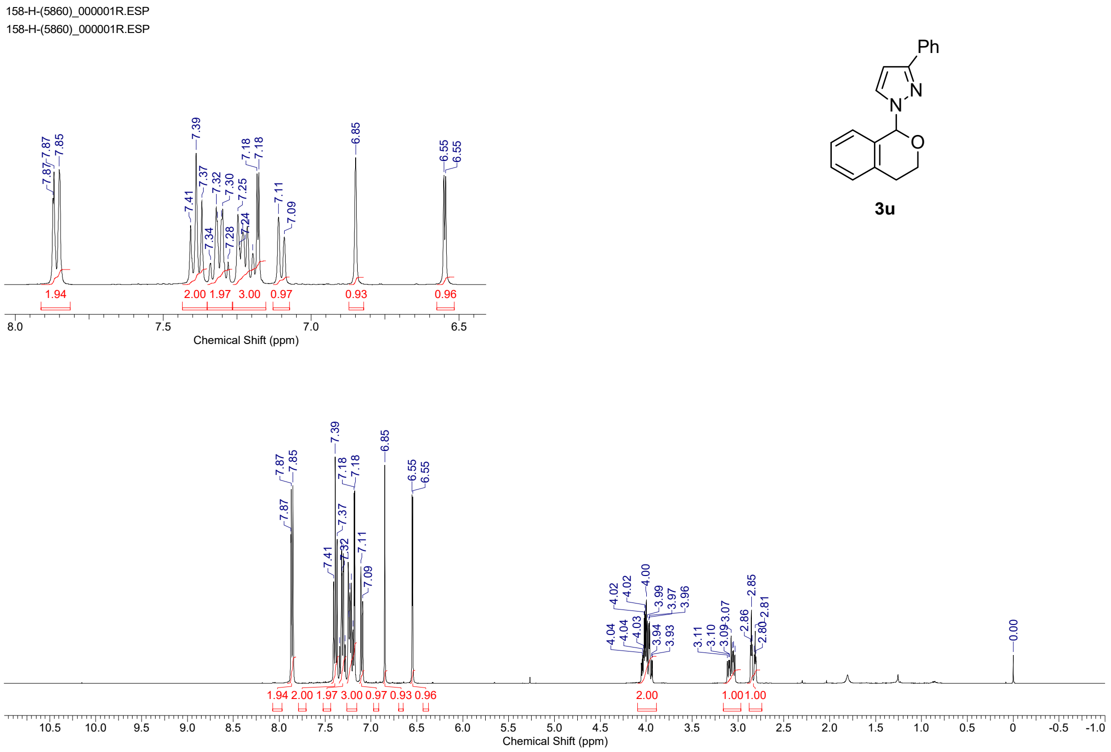

Figure S50. ^{1}H NMR spectra of compound **3u**

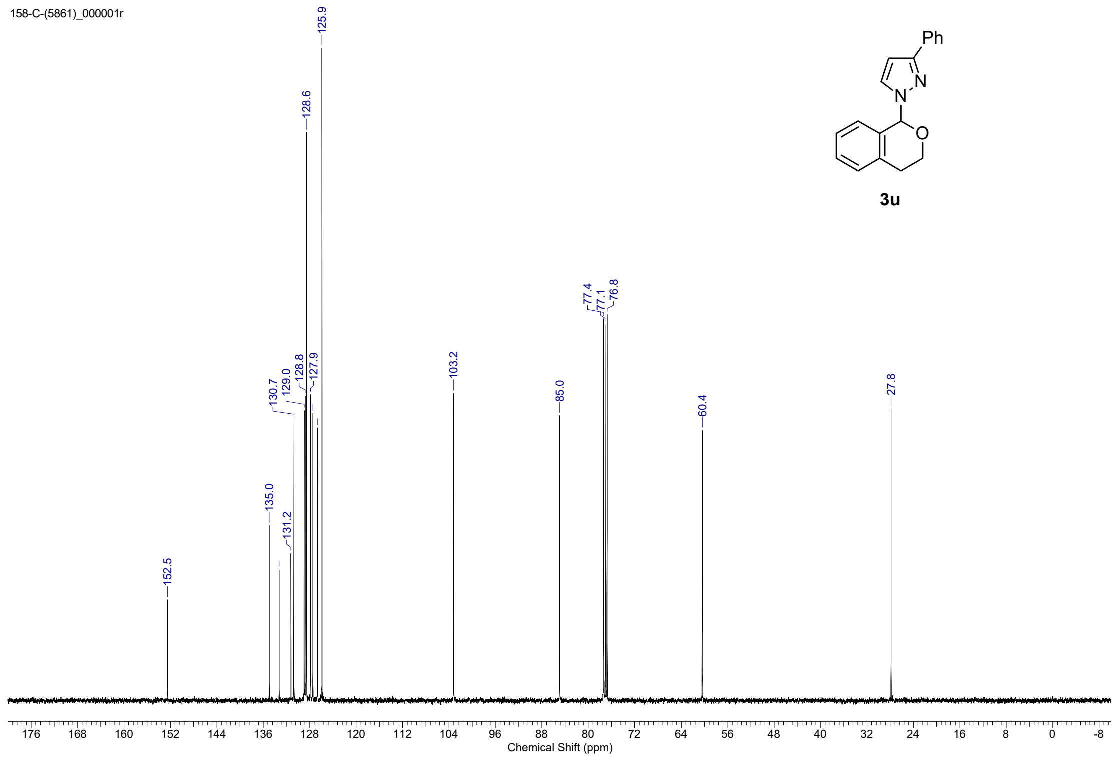

Figure S51. ^{13}C NMR spectra of compound **3u**

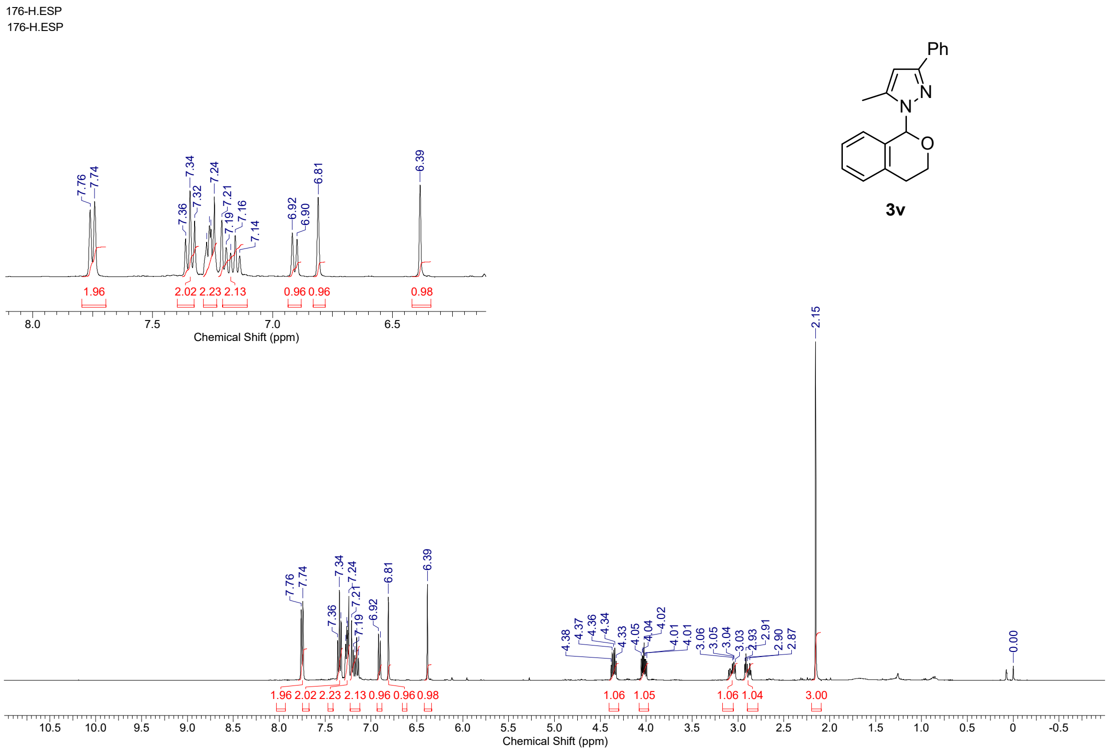

Figure S52. ^{1}H NMR spectra of compound **3v**

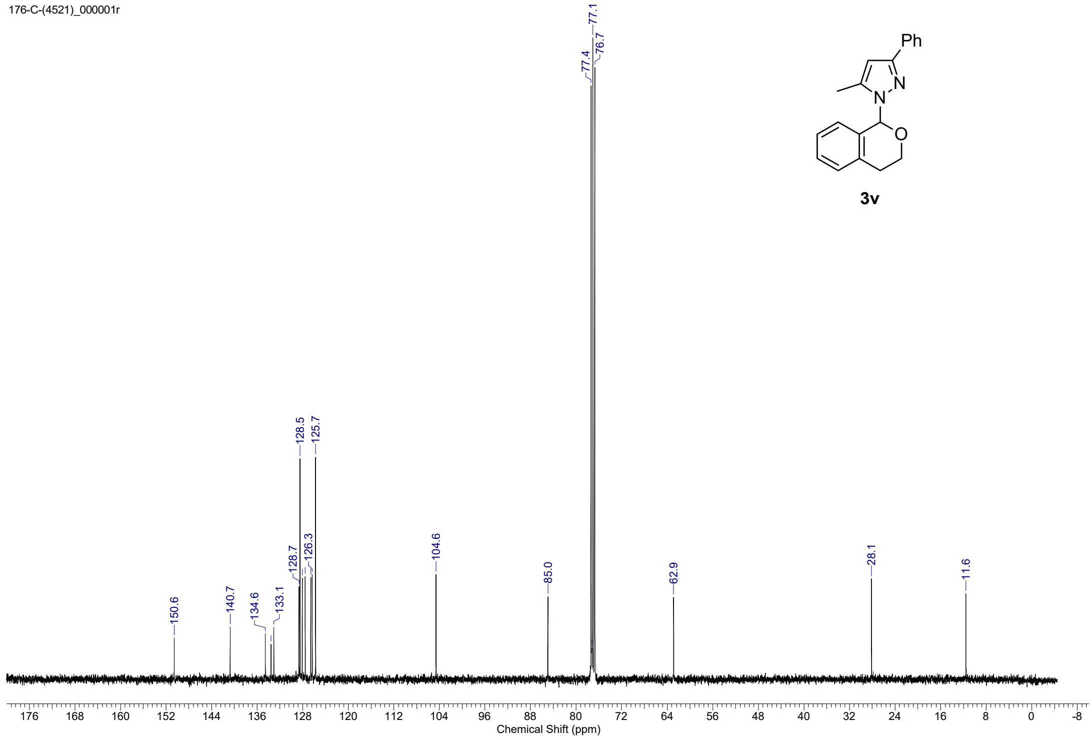

Figure S53. ^{13}C NMR spectra of compound **3v**

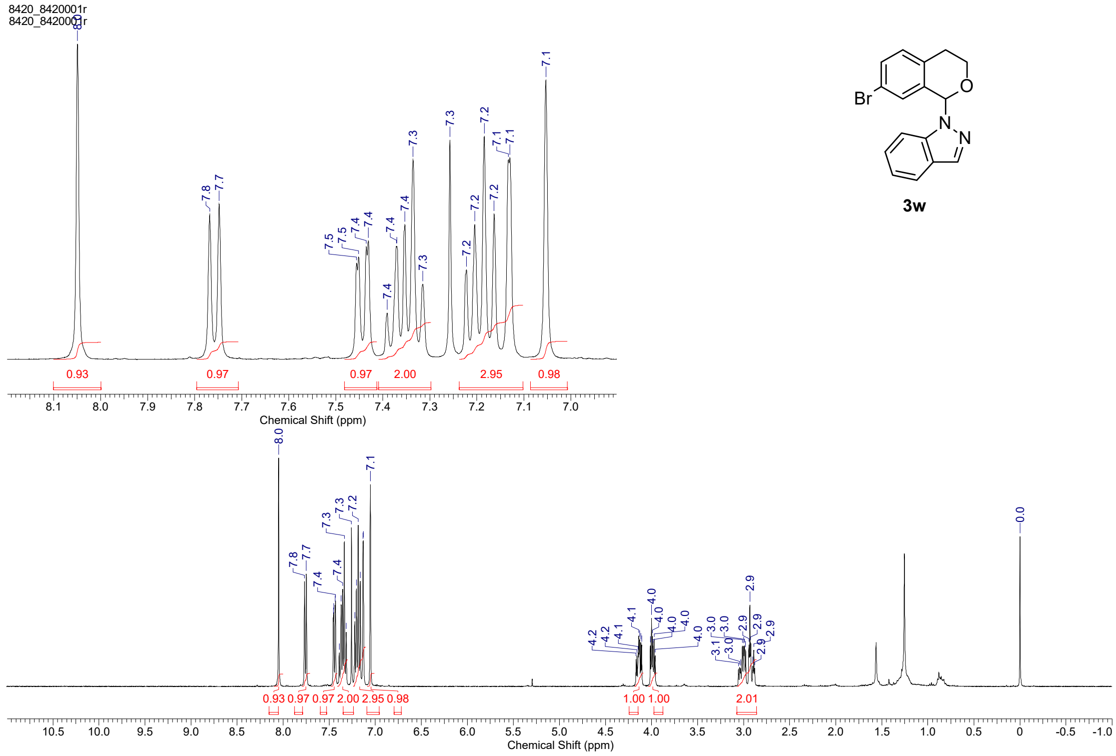

Figure S54. ^{1}H NMR spectra of compound **3w**

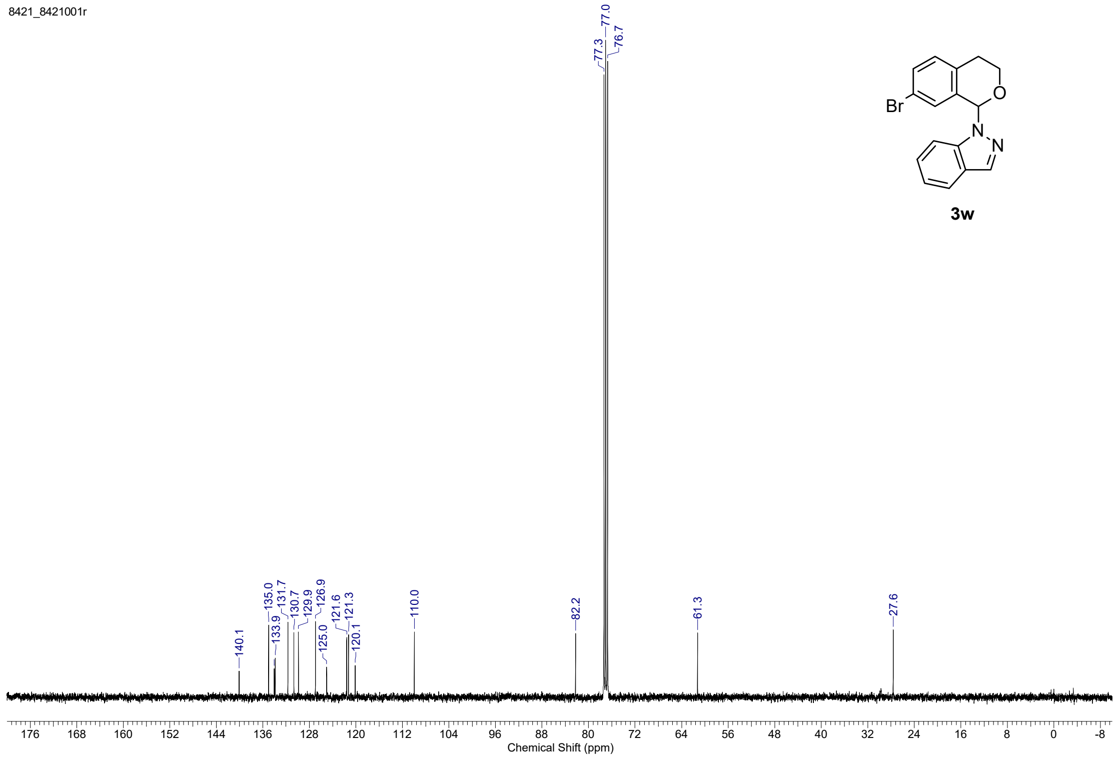

Figure S55. ^{13}C NMR spectra of compound **3w**

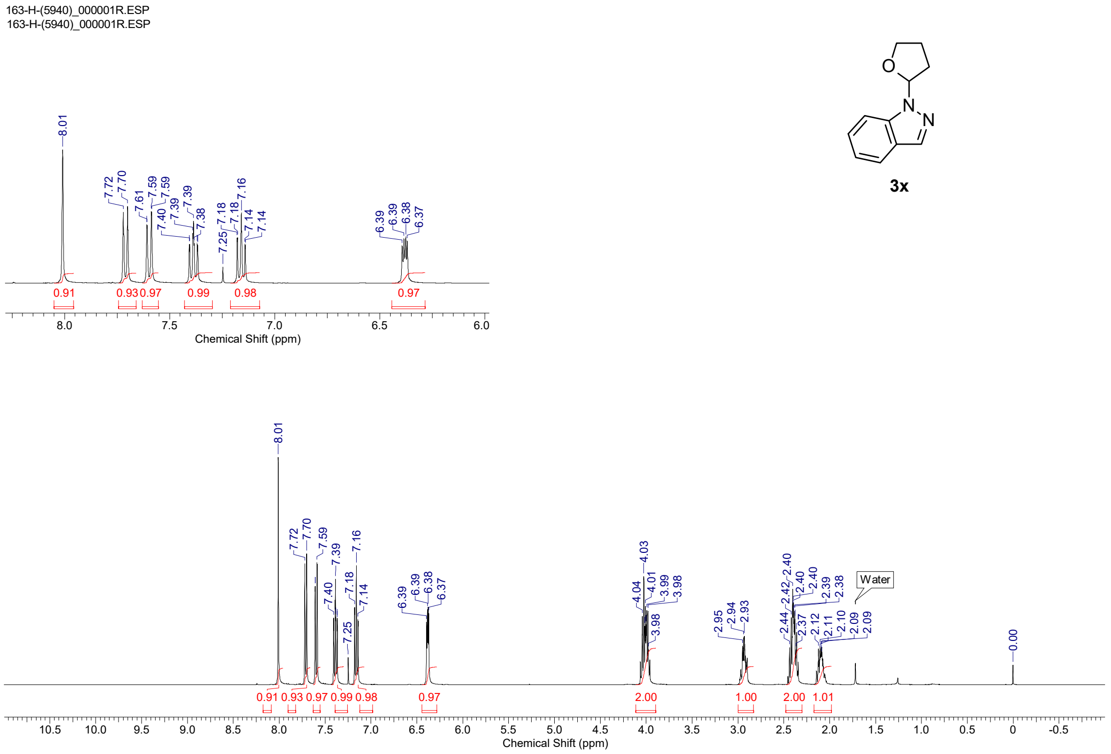

Figure S56. ^{1}H NMR spectra of compound **3x**

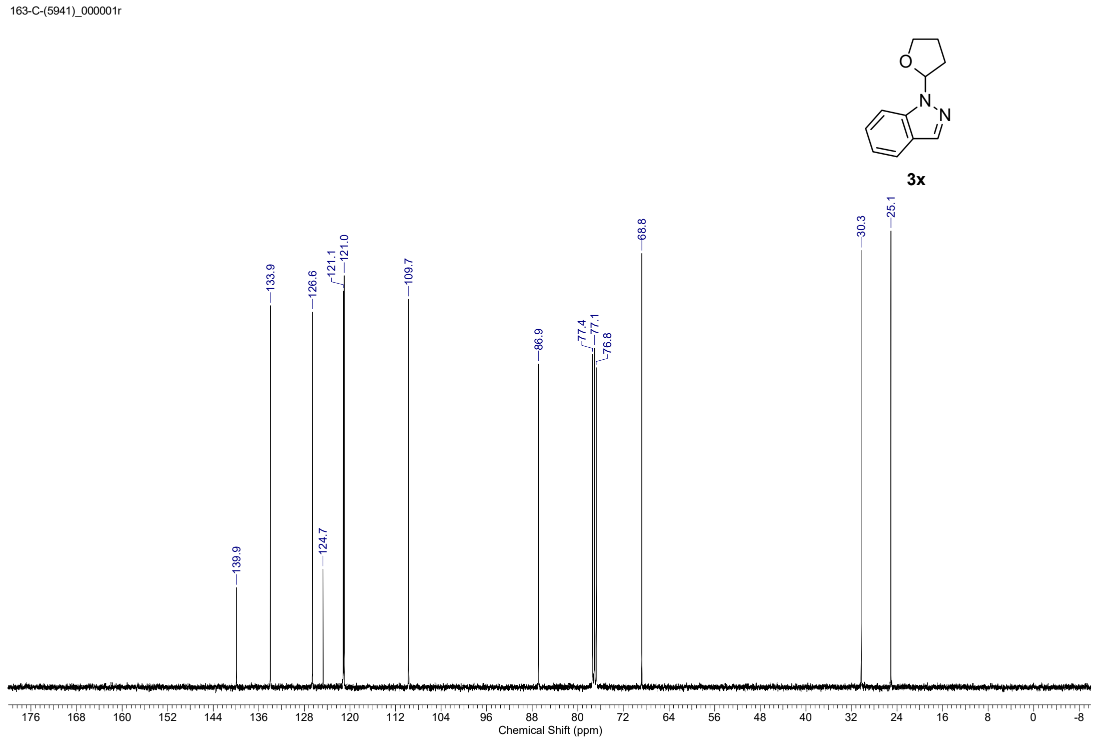

Figure S57. ^{13}C NMR spectra of compound **3x**

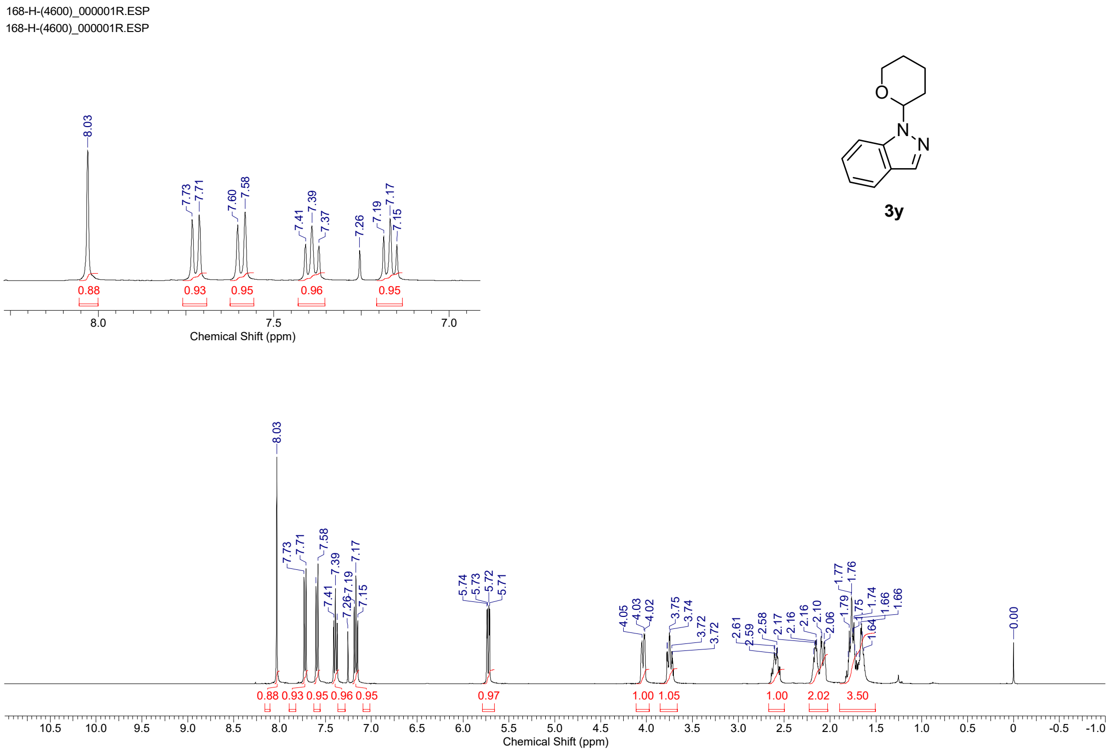

Figure S58. ^{1}H NMR spectra of compound **3y**

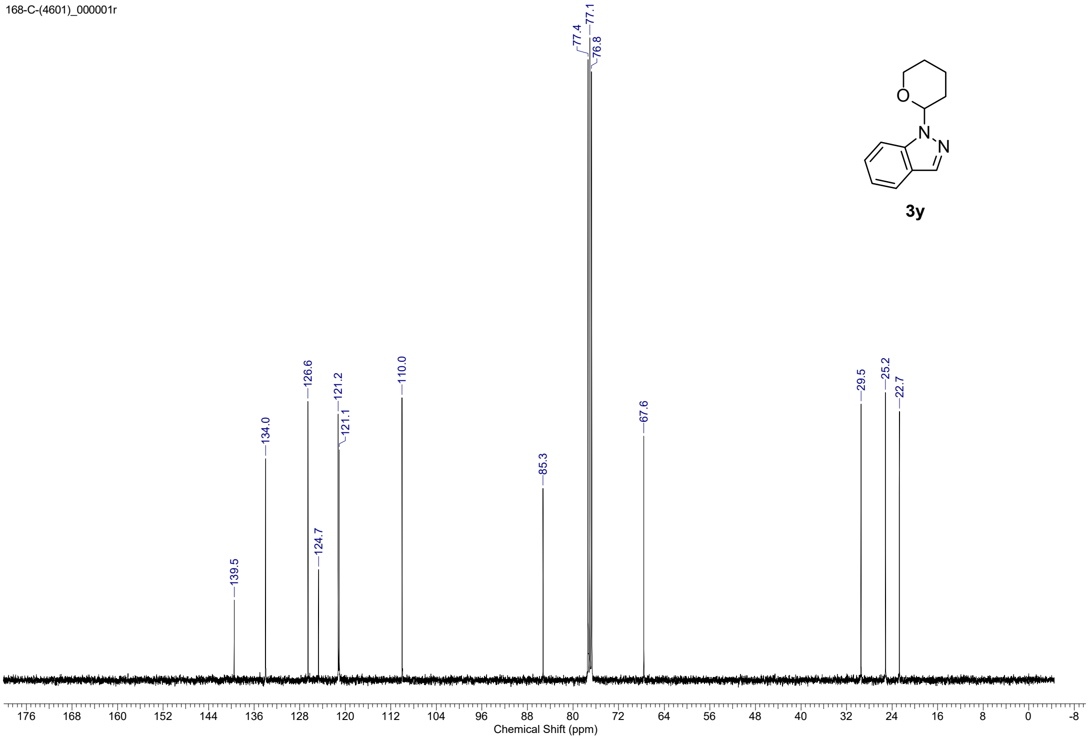

Figure S59. ^{13}C NMR spectra of compound **3y**

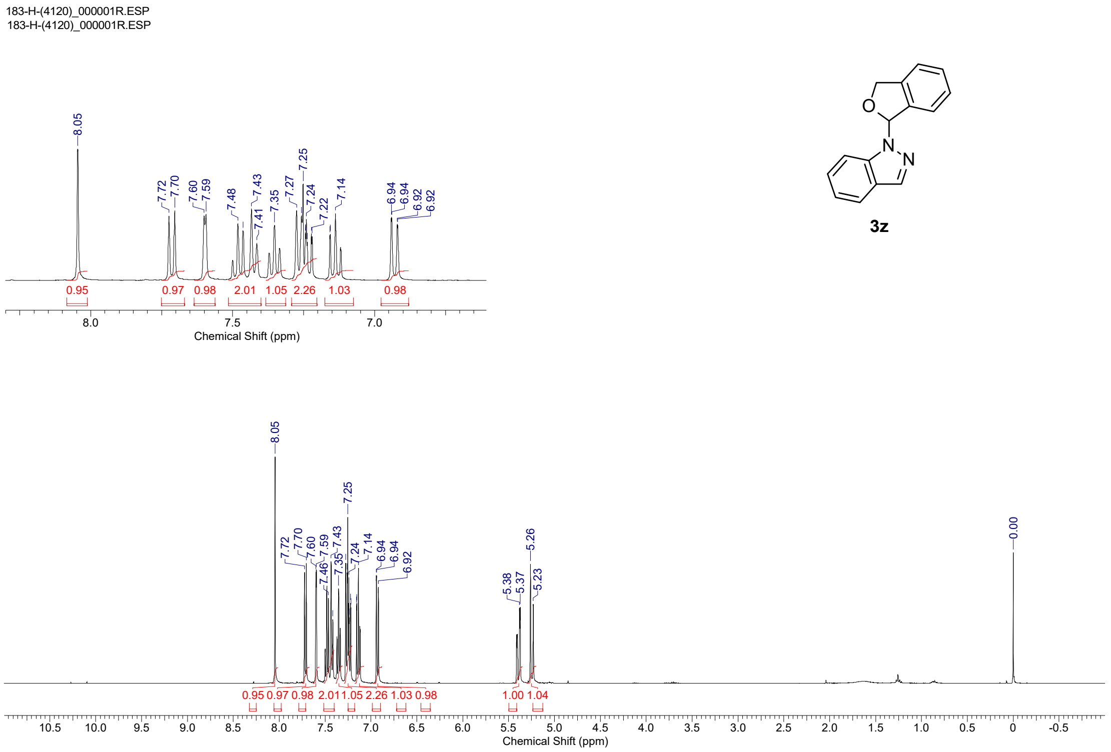

Figure S60. ^{1}H NMR spectra of compound **3z**

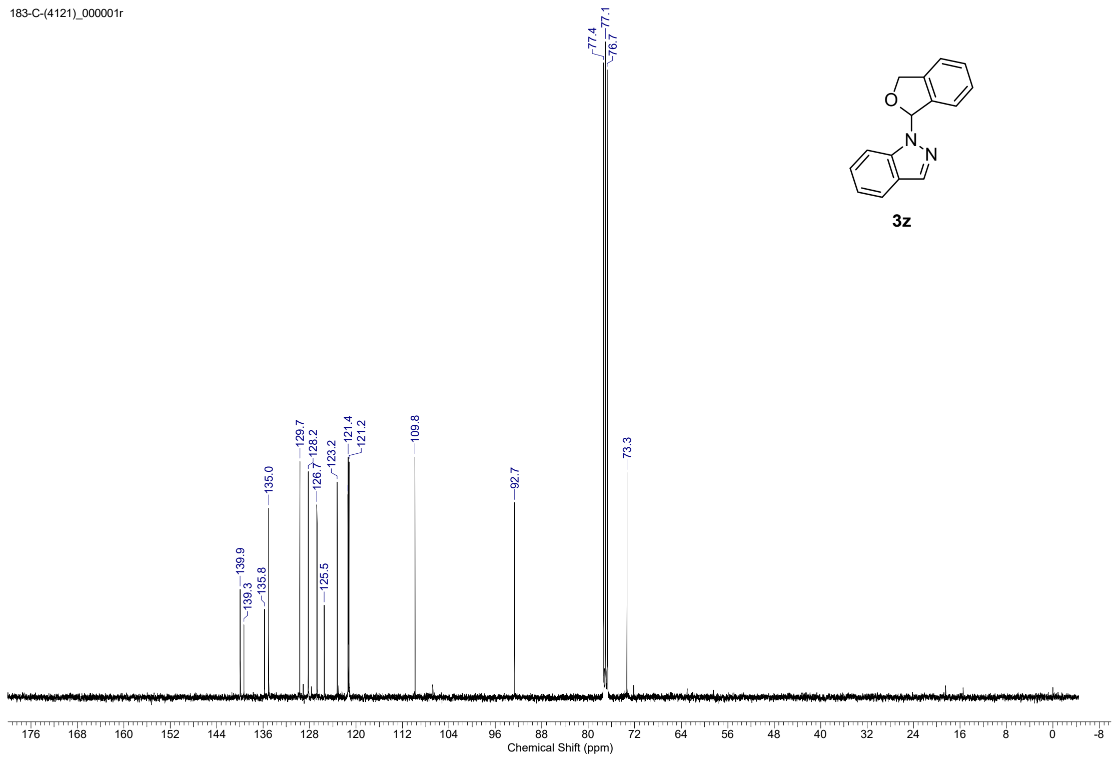

Figure S61. ^{13}C NMR spectra of compound **3z**

6. HRMS Spectra for the Products

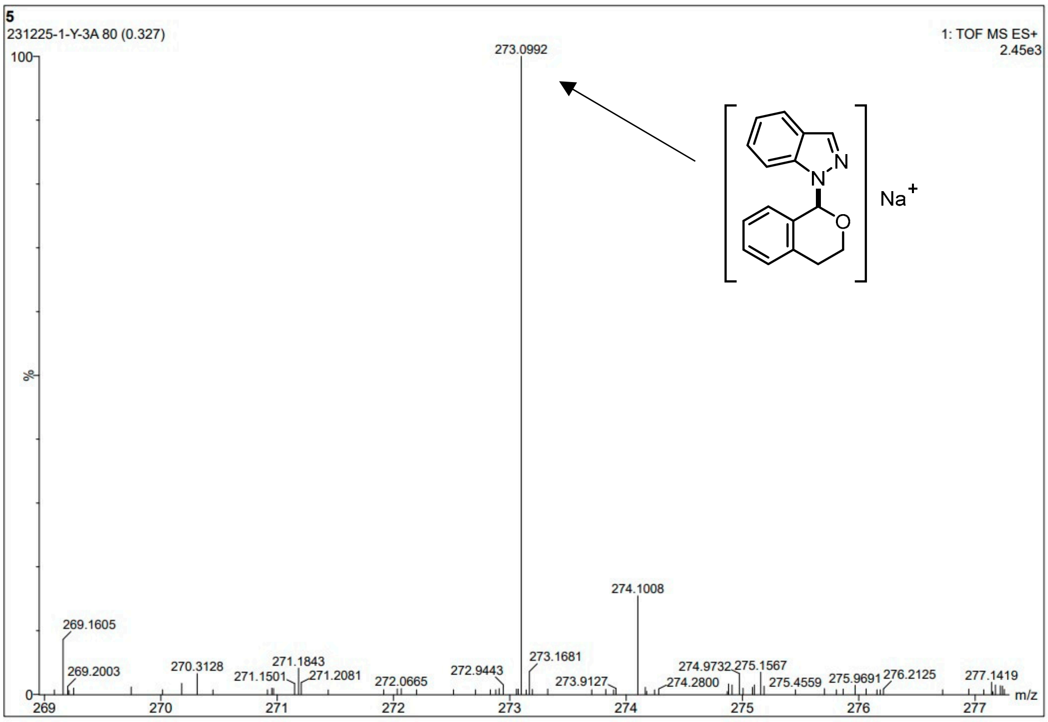

Figure S62. HRMS Spectra for **3a**

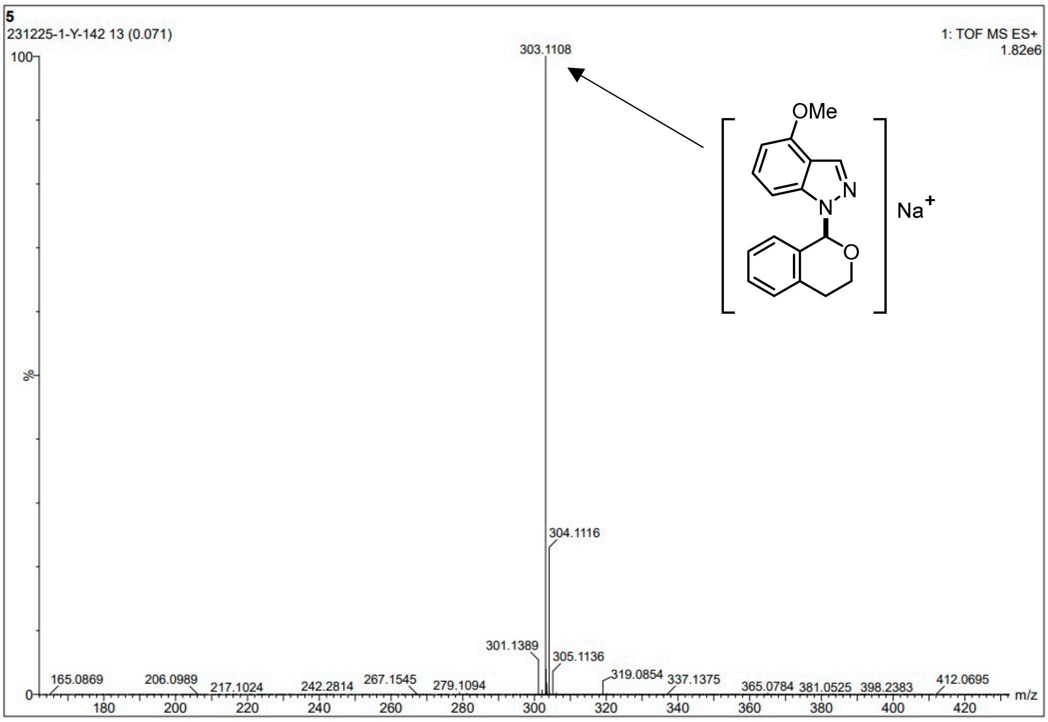

Figure S63. HRMS Spectra for **3b**

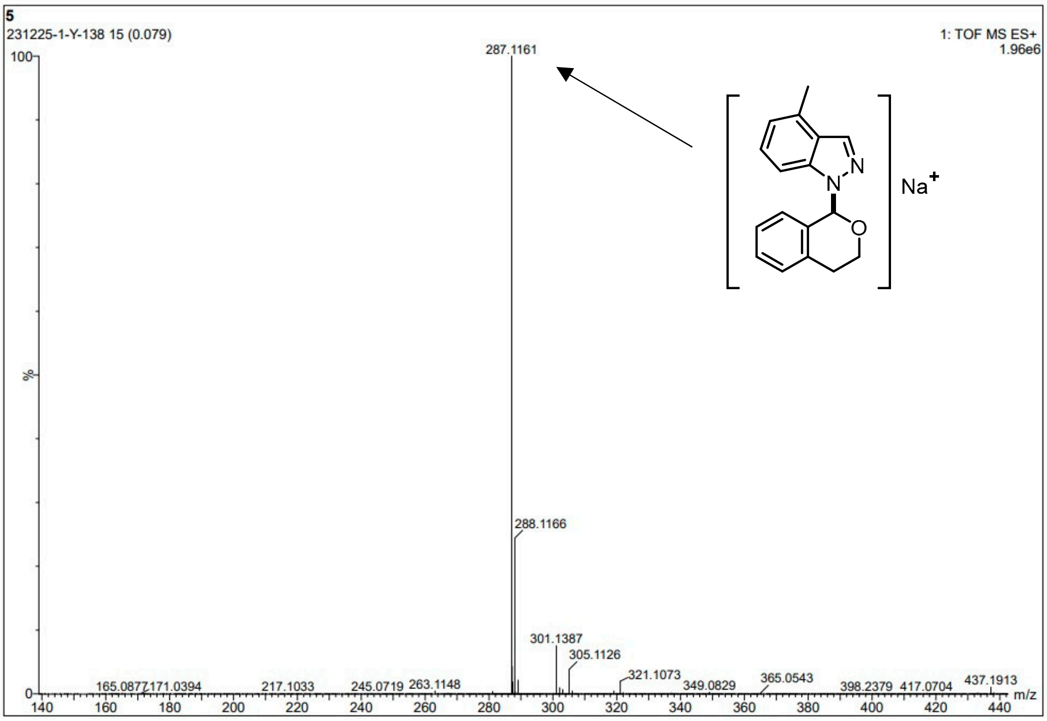

Figure S64. HRMS Spectra for **3c**

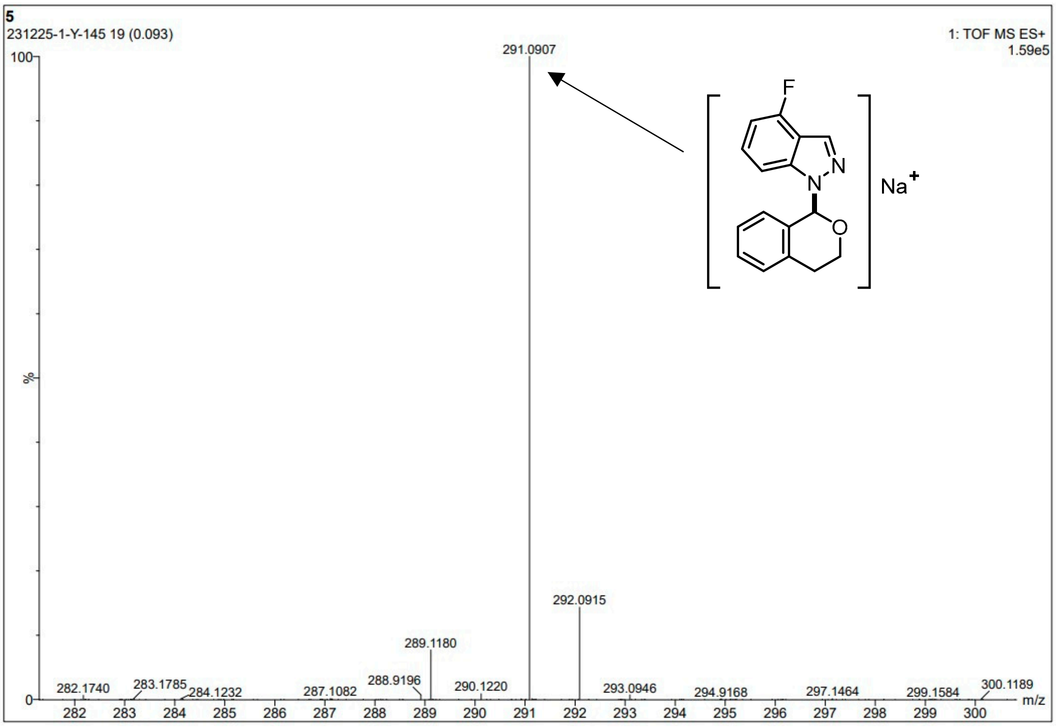

Figure S65. HRMS Spectra for **3d**

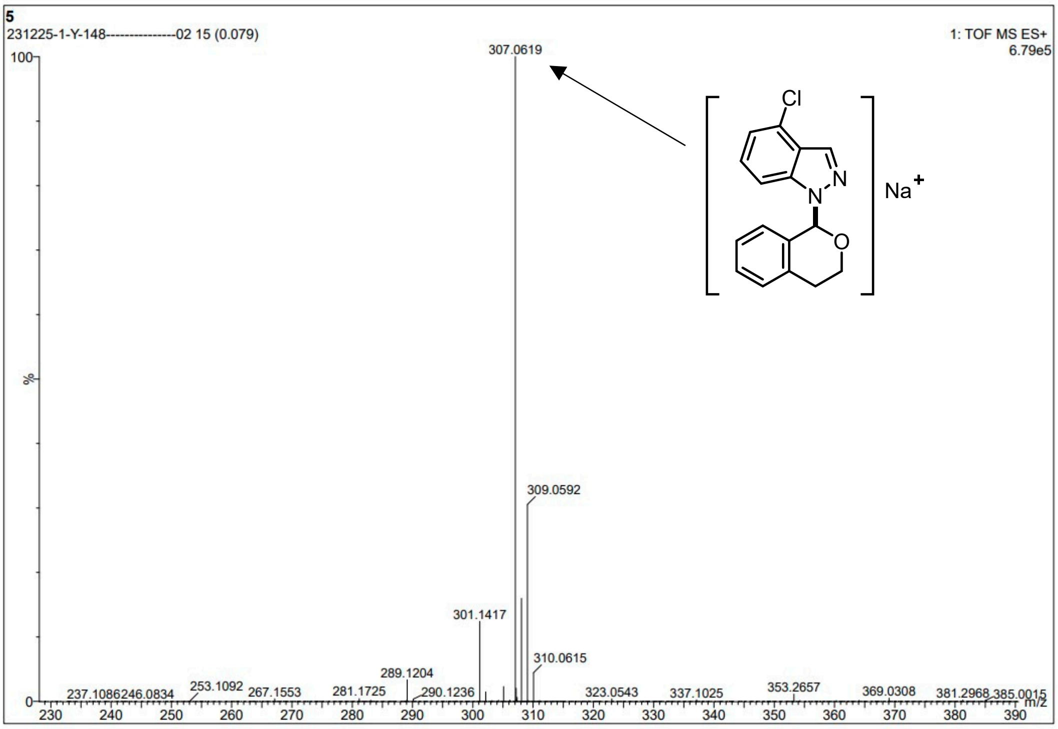

Figure S66. HRMS Spectra for **3e**

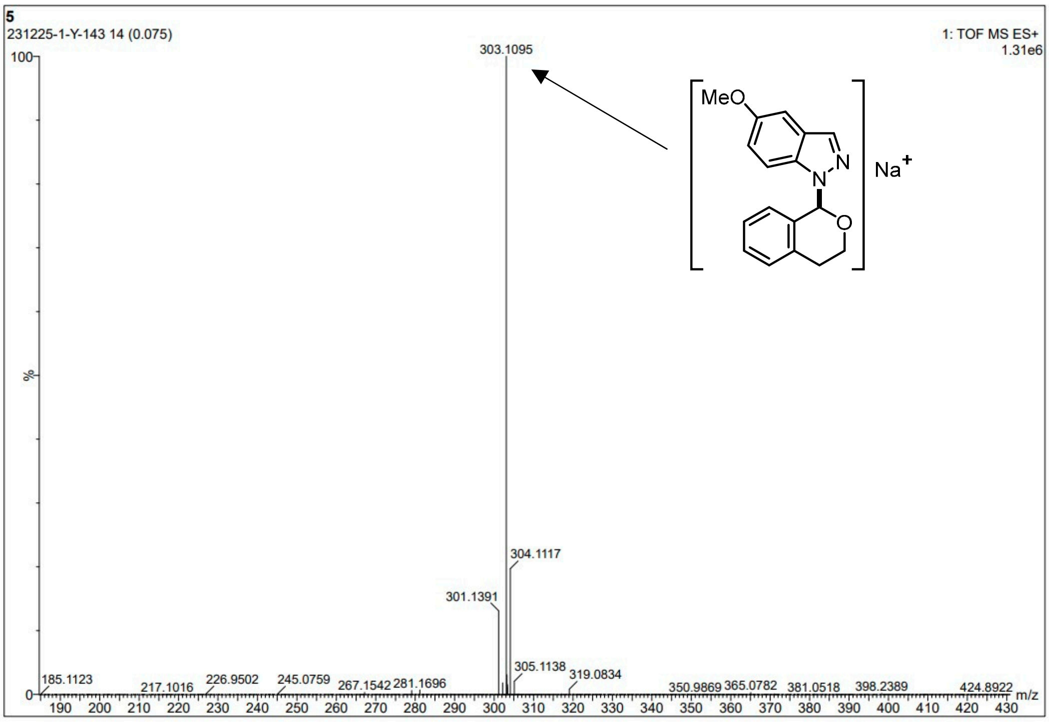

Figure S67. HRMS Spectra for **3f**

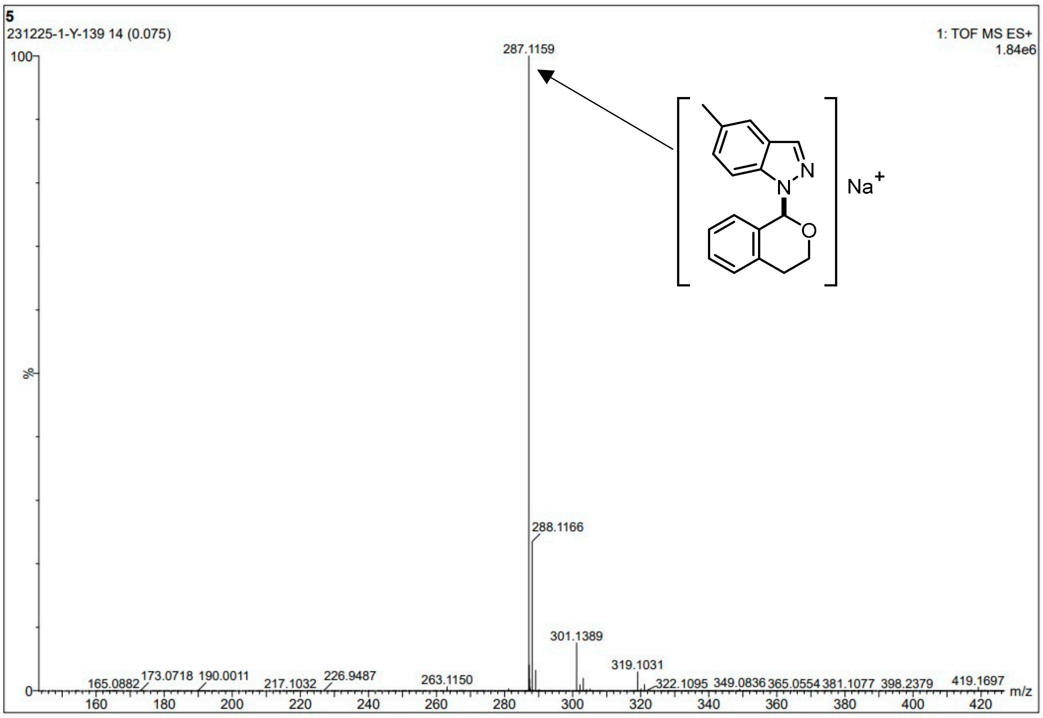

Figure S68. HRMS Spectra for **3g**

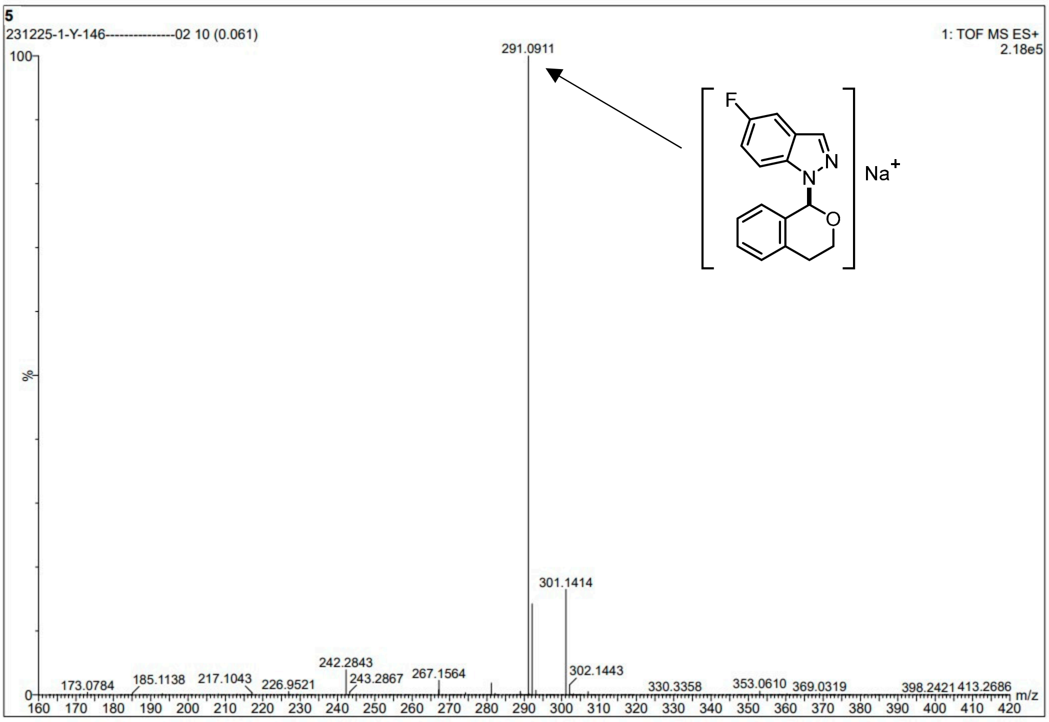

Figure S69. HRMS Spectra for **3h**

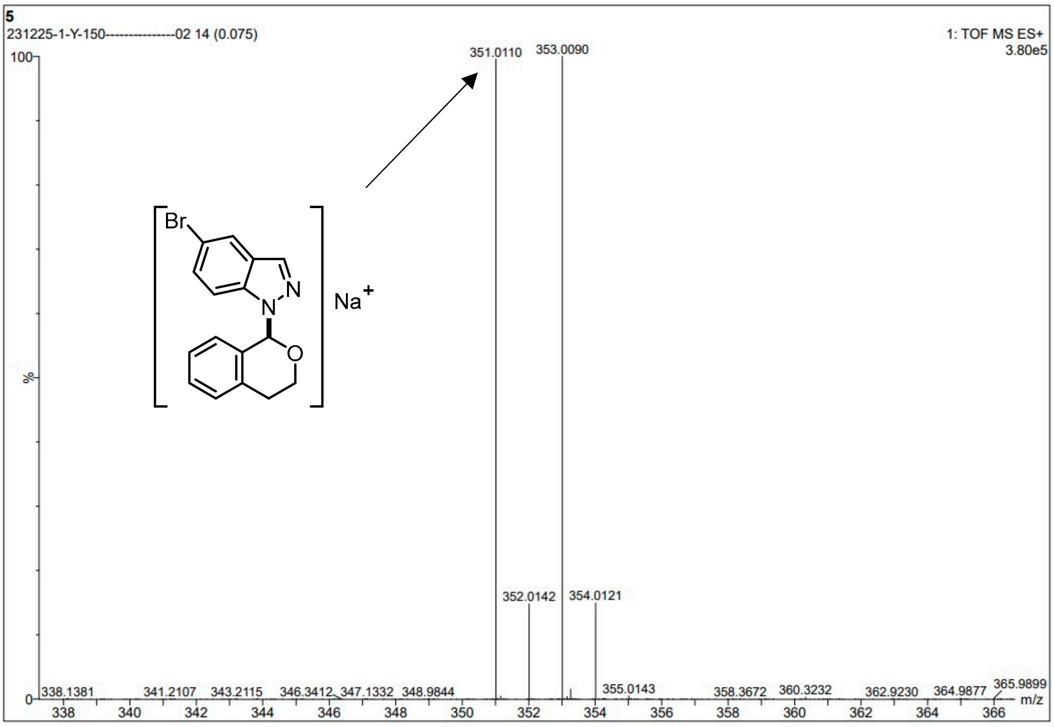

Figure S70. HRMS Spectra for **3i**

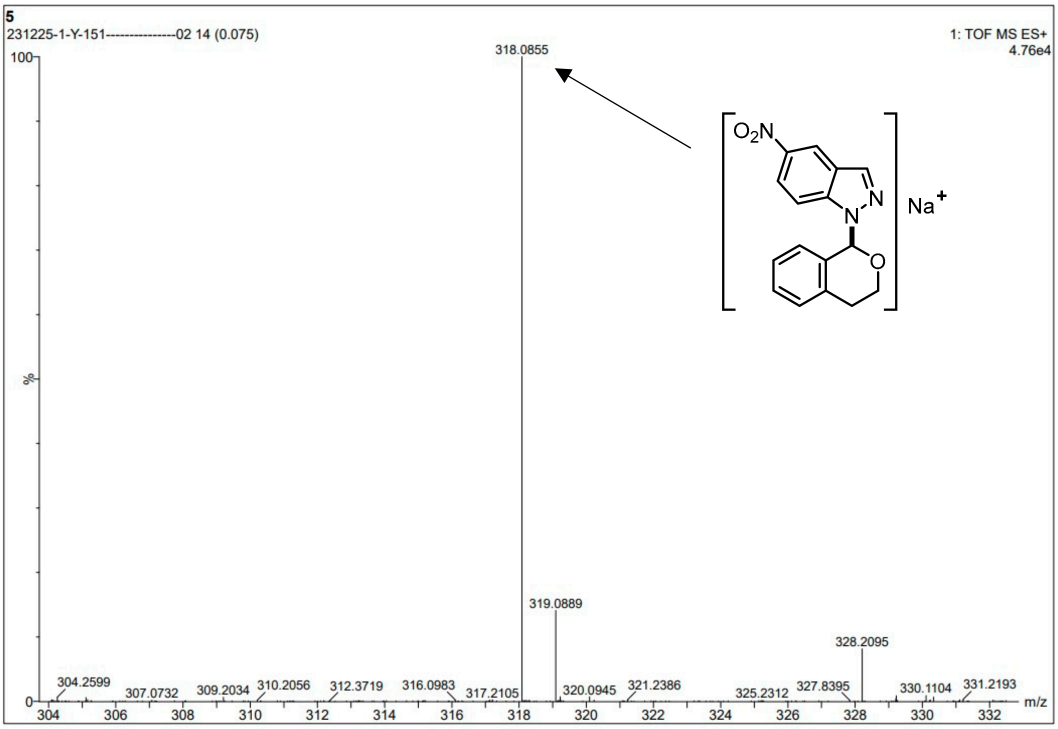

Figure S71. HRMS Spectra for **3j**

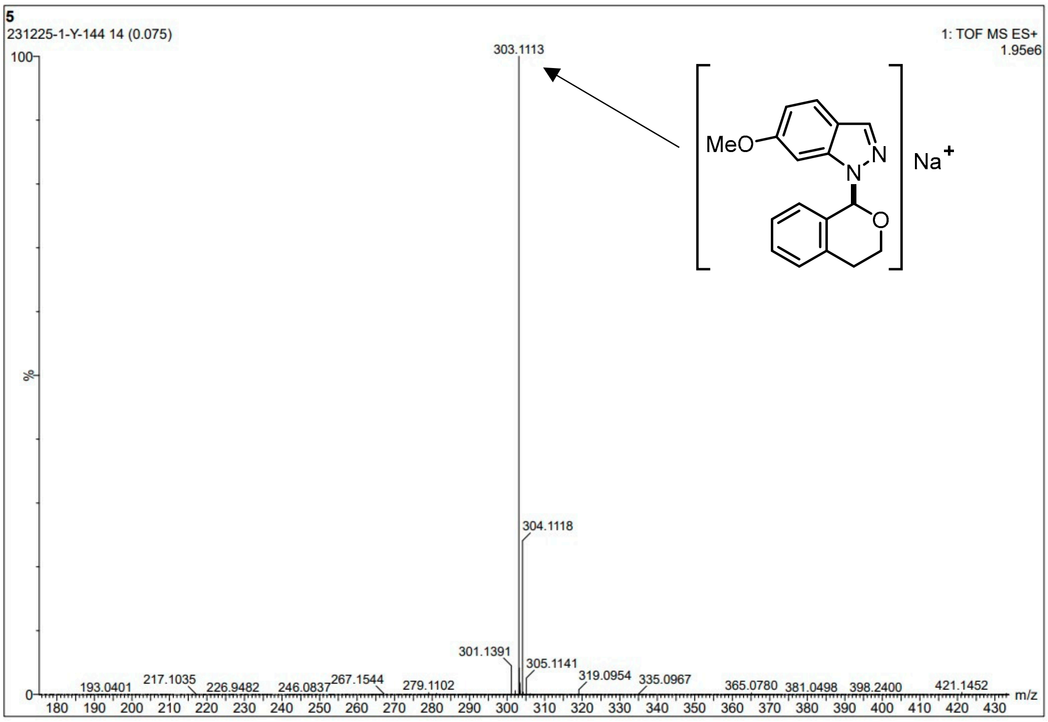

Figure S72. HRMS Spectra for **3k**

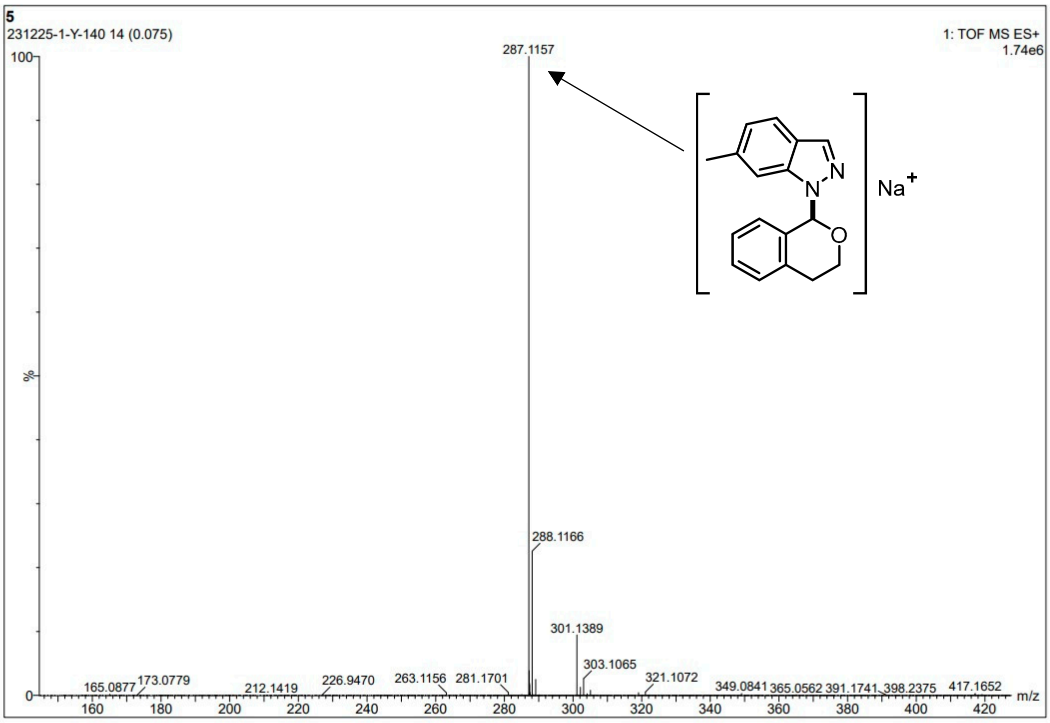

Figure S73. HRMS Spectra for **3l**

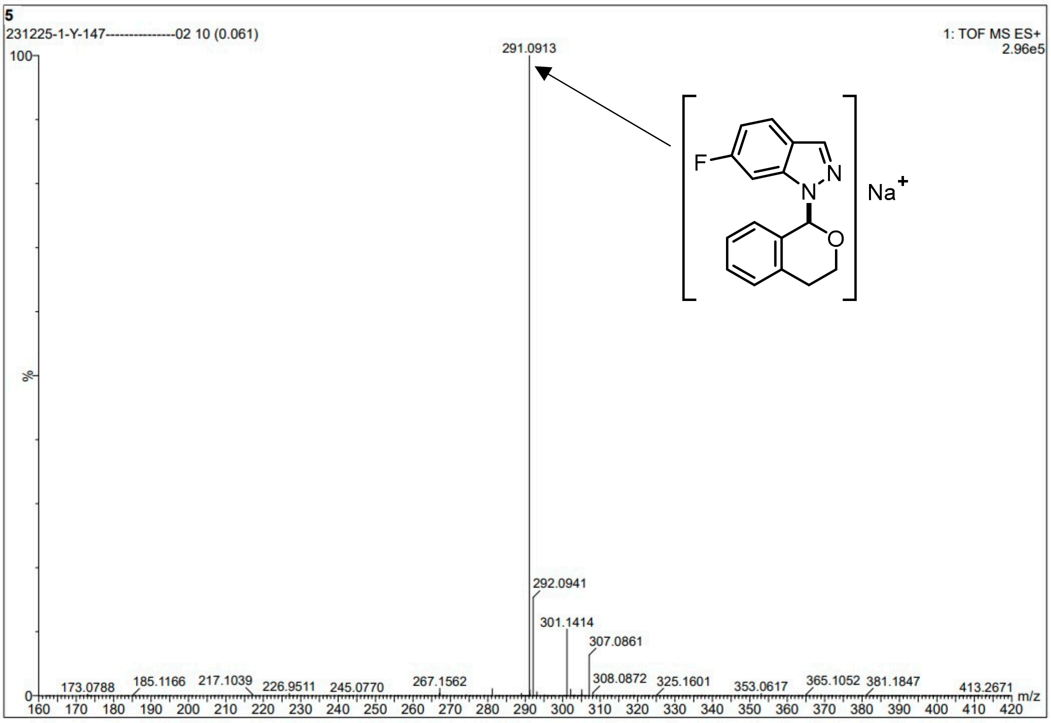

Figure S74. HRMS Spectra for **3m**

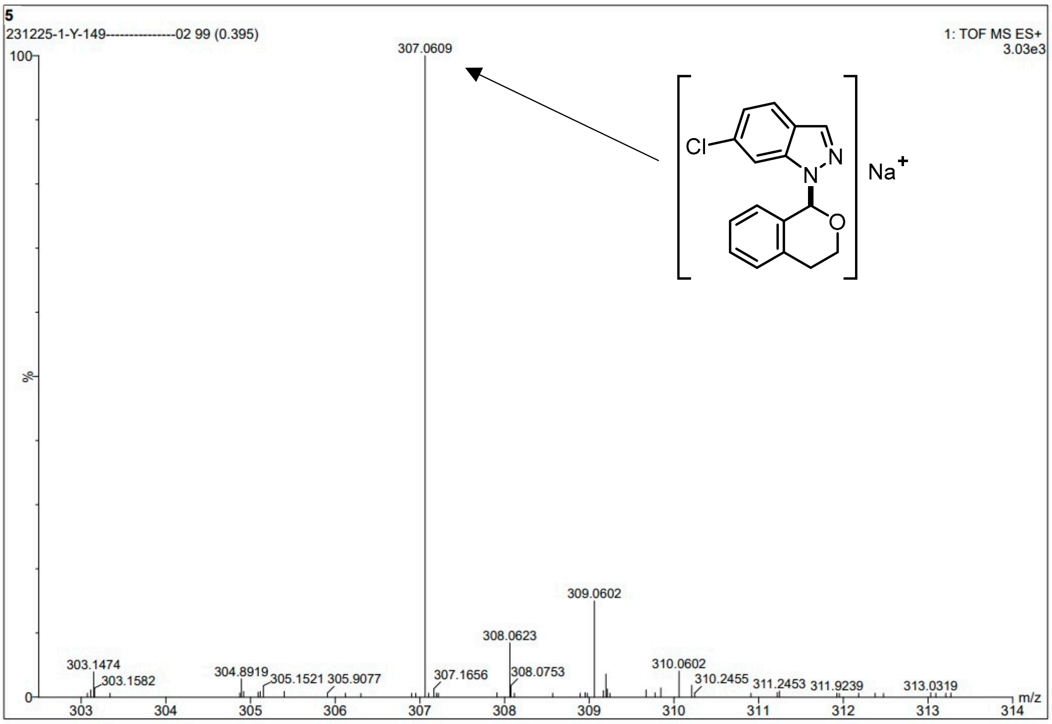

Figure S75. HRMS Spectra for **3n**

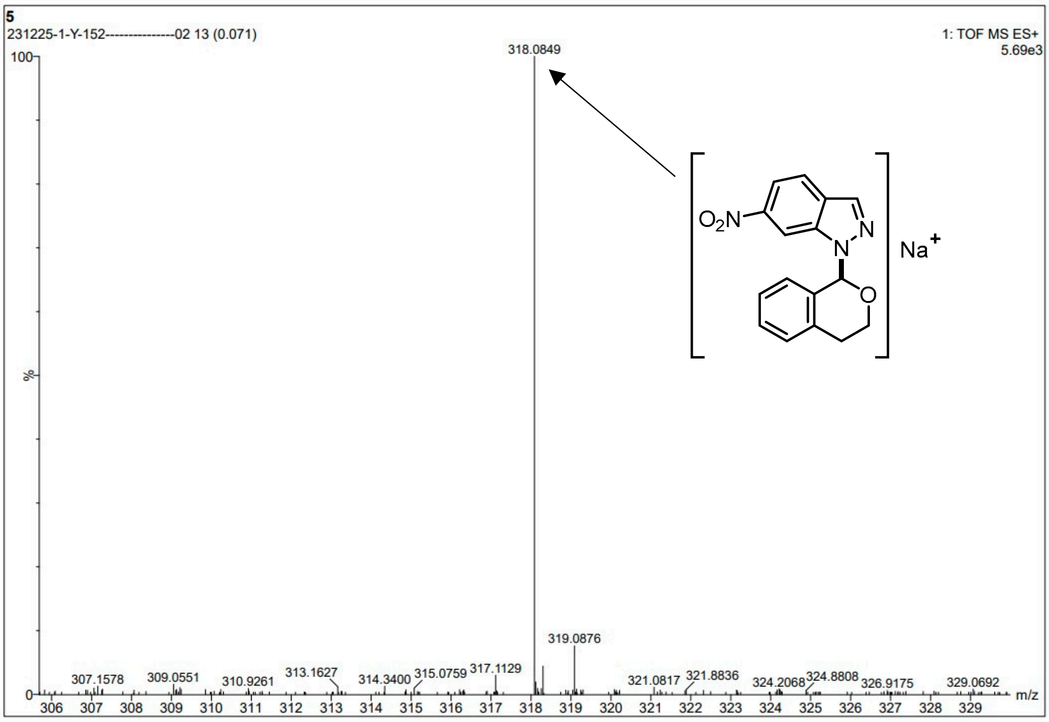

Figure S76. HRMS Spectra for **3o**

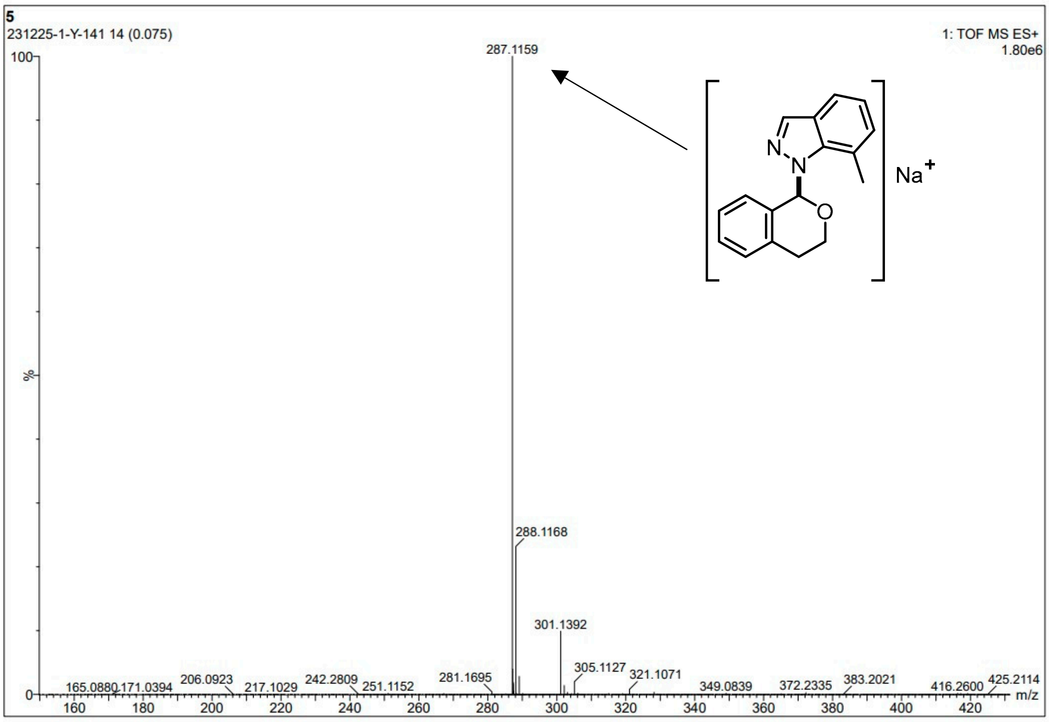

Figure S77. HRMS Spectra for **3p**

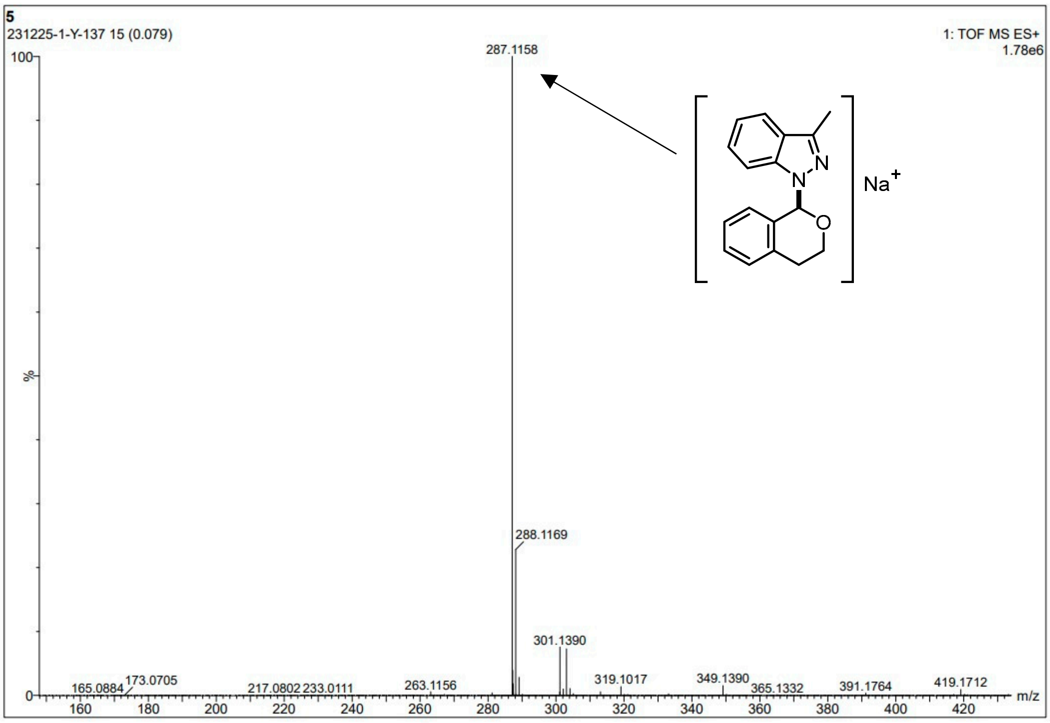

Figure S78. HRMS Spectra for **3r**

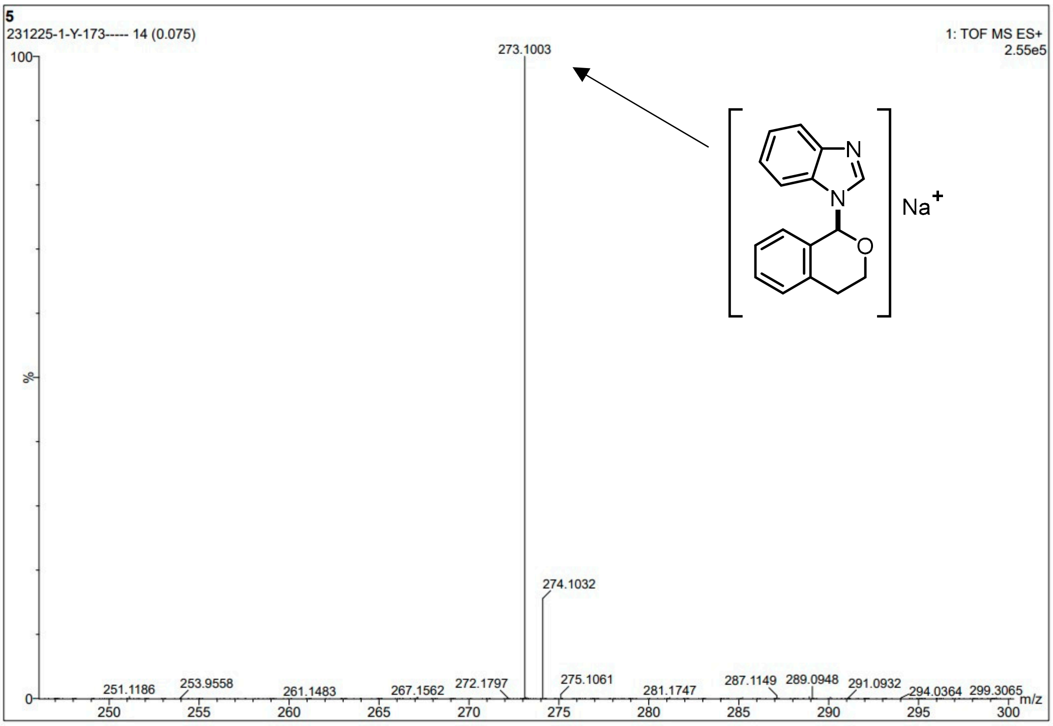

Figure S79. HRMS Spectra for **3s**

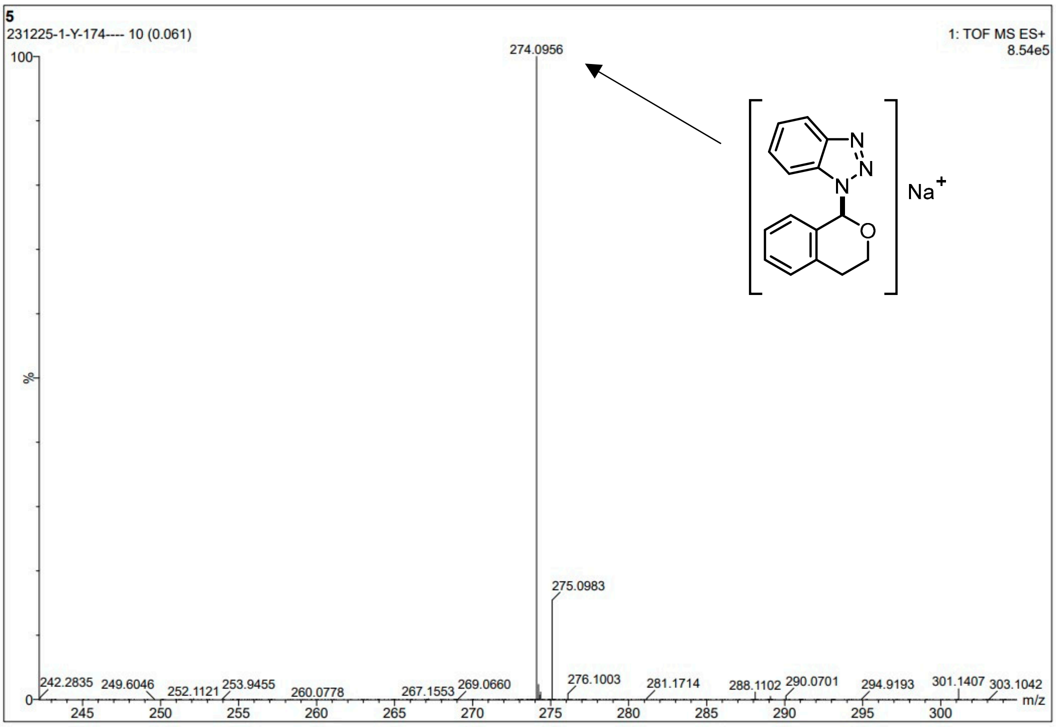

Figure S80. HRMS Spectra for **3t**

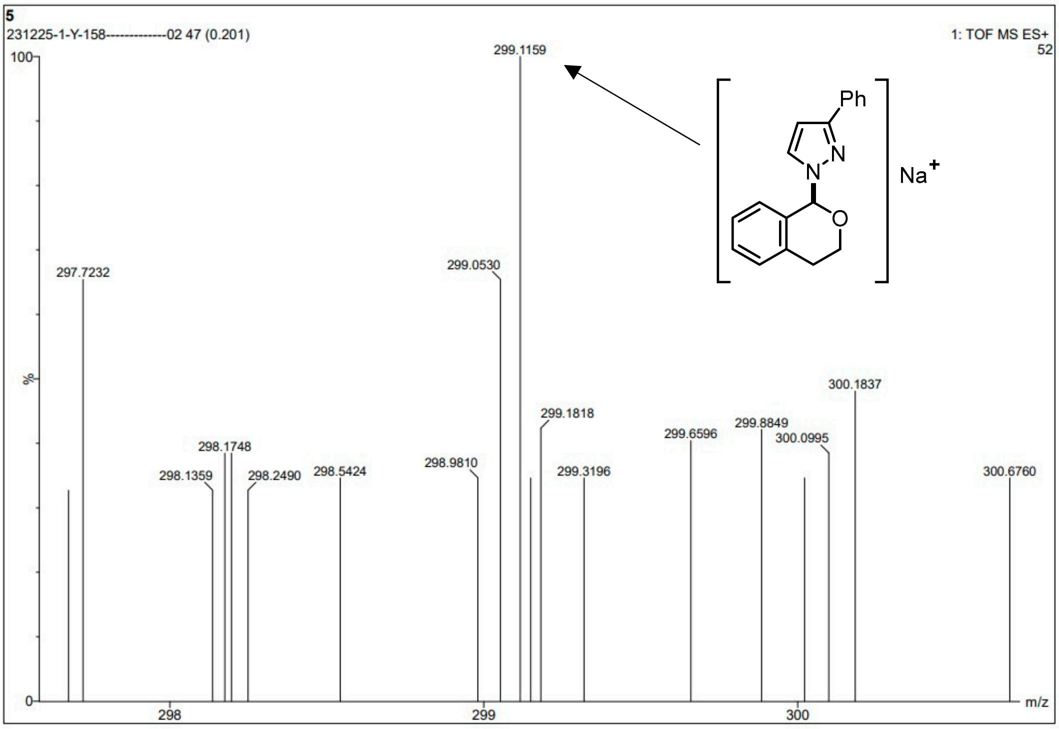

Figure S81. HRMS Spectra for **3u**

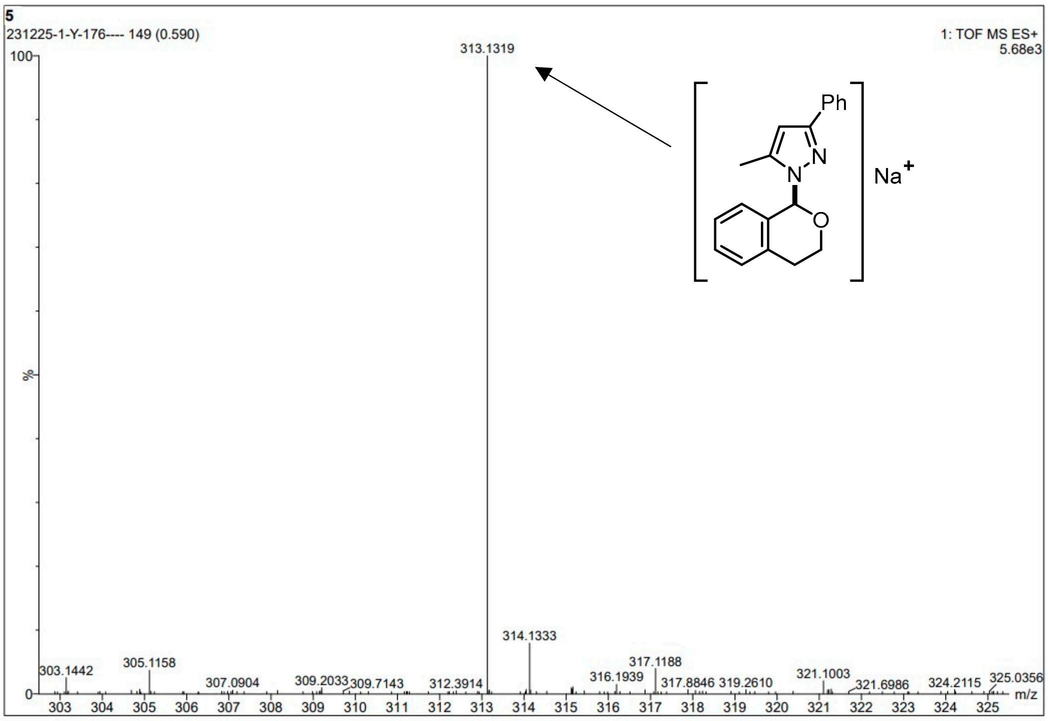

Figure S82. HRMS Spectra for **3v**

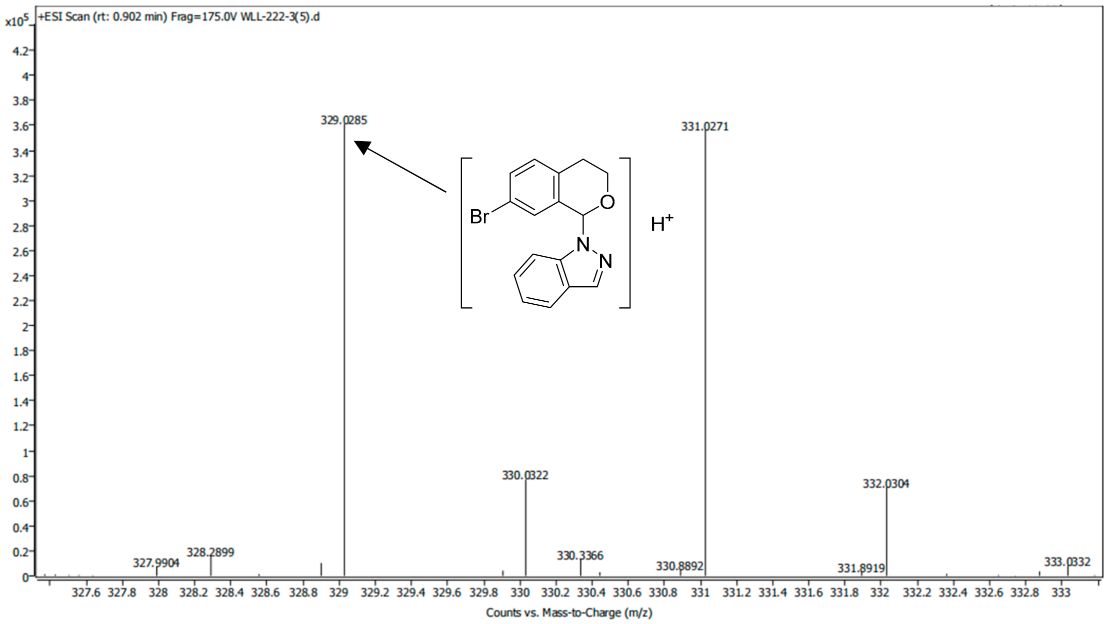

Figure S83. HRMS Spectra for **3w**

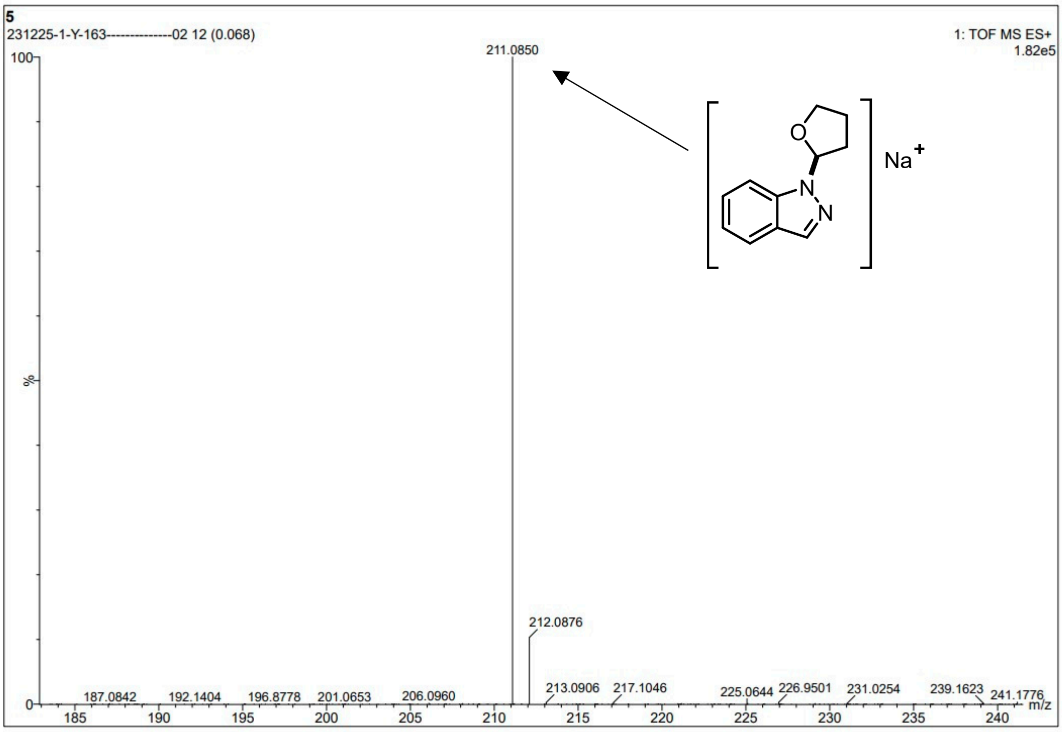

Figure S84. HRMS Spectra for **3x**

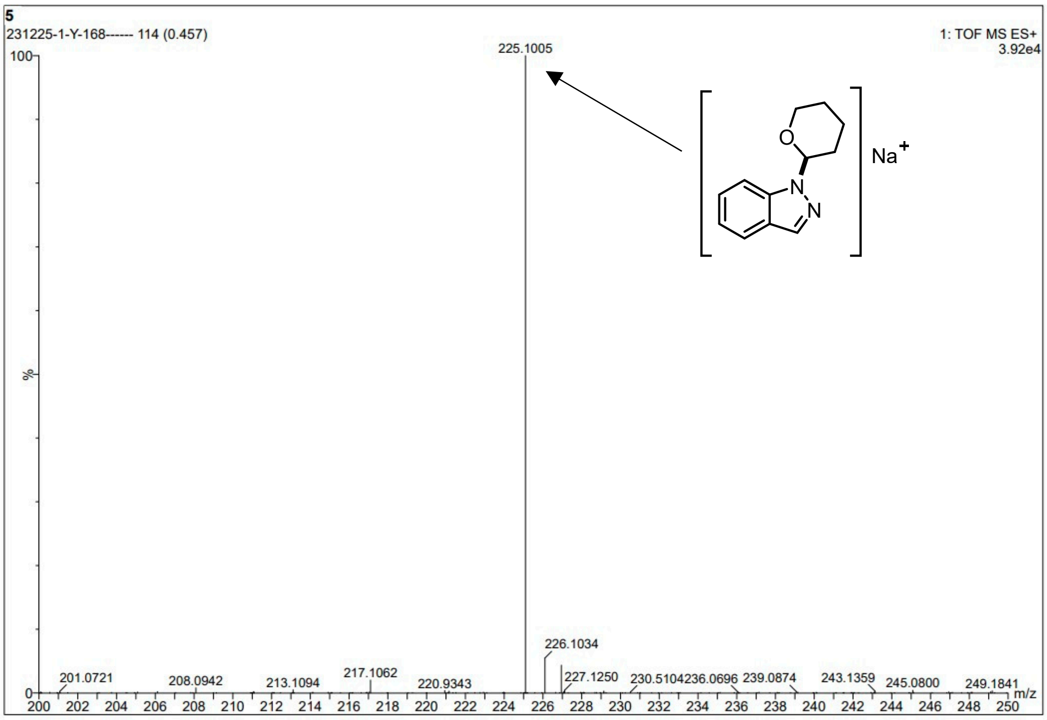

Figure S85. HRMS Spectra for **3y**

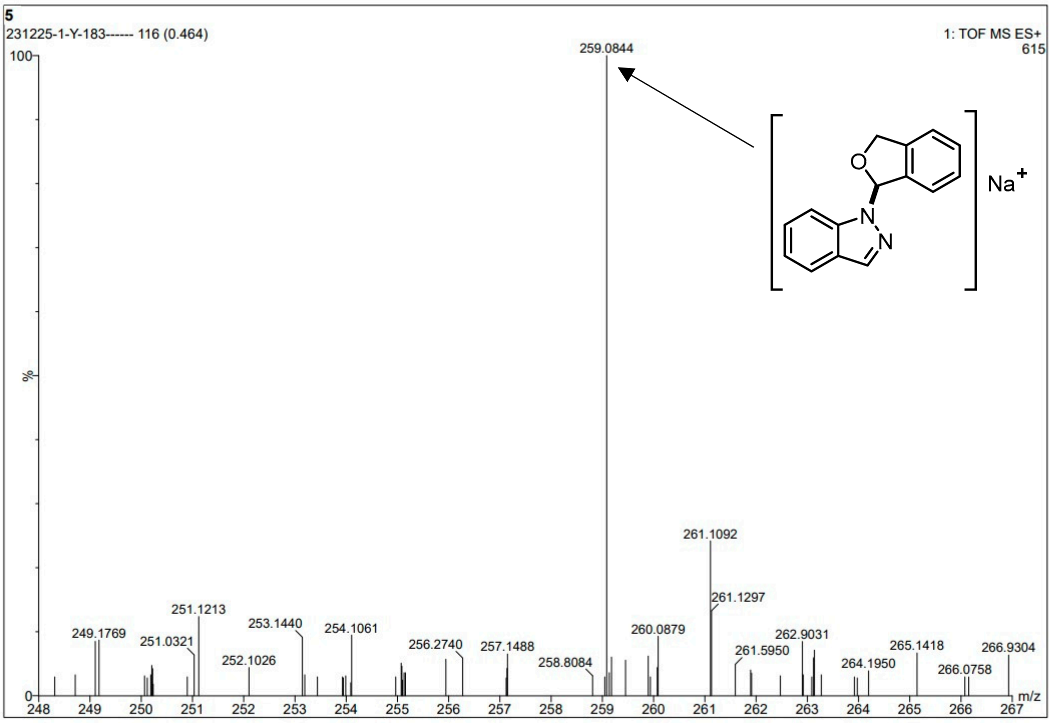

Figure S86. HRMS Spectra for **3z**

7. Determination of Structure of 3a

The structure of **3a** was determined by the X-ray diffraction. Recrystallized from dichloromethane/ethanol. Further information can be found in the CIF file. This crystal was deposited in the Cambridge Crystallographic Data Centre and assigned as CCDC 2308694.

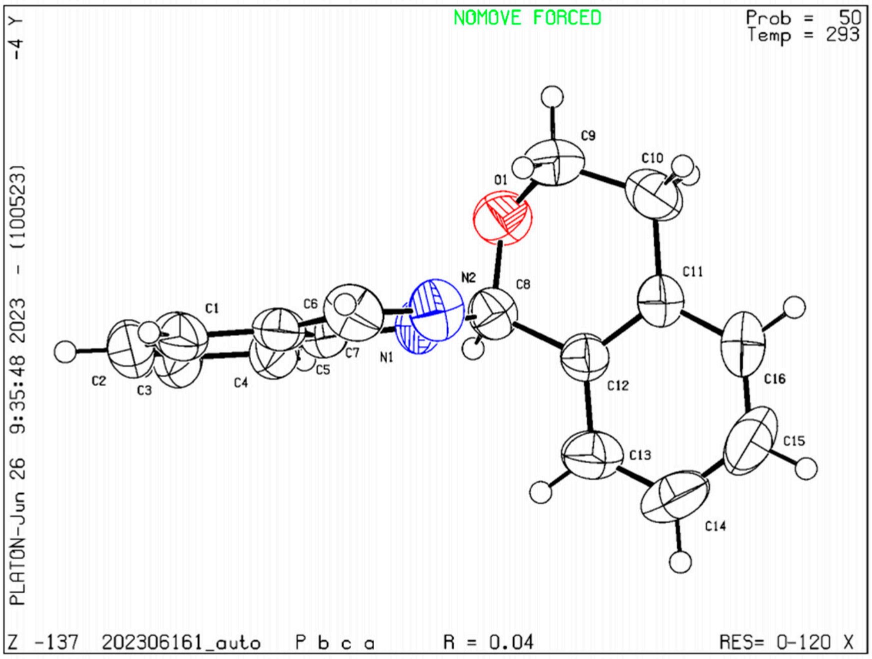

Figure S87. X-ray structure of compound **3a** (CCDC 2308694)

Table S1. Crystal data and structure refinement for **3a**

Identification code	202306161_auto
Empirical formula	$C_{16}H_{14}N_2O$
Formula weight	250.29
Temperature/K	293(2)
Crystal system	orthorhombic
Space group	Pbca
a/Å	8.4948(5)
b/Å	10.3689(6)
c/Å	29.4813(14)
α/°	90
β/°	90
γ/°	90
Volume/Å^3	2596.8(2)
Z	8
ρ_{calc}g/cm^3	1.280

μ/mm^{-1}	0.646
F(000)	1056.0
Crystal size/mm^3	0.16 × 0.11 × 0.1
Radiation	CuKα (λ = 1.54184)
2Θ range for data collection/°	12.008 to 134.142
Index ranges	-10 ≤ h ≤ 10, -11 ≤ k ≤ 12, -35 ≤ l ≤ 19
Reflections collected	5541
Independent reflections	2310 [R_{int} = 0.0220, R_{sigma} = 0.0267]
Data/restraints/parameters	2310/0/173
Goodness-of-fit on F^2	1.035
Final R indexes [I>=2σ (I)]	R_1 = 0.0430, wR_2 = 0.1152
Final R indexes [all data]	R_1 = 0.0560, wR_2 = 0.1279
Largest diff.peak/hole / eÅ^{-3}	0.16/-0.15
